# Antiprotozoal Activity of Azabicyclo-Nonanes Linked to Tetrazole or Sulfonamide Cores

**DOI:** 10.3390/molecules27196217

**Published:** 2022-09-21

**Authors:** Johanna Dolensky, Clemens Hinteregger, Andreas Leitner, Werner Seebacher, Robert Saf, Ferdinand Belaj, Pascal Mäser, Marcel Kaiser, Robert Weis

**Affiliations:** 1 Pharmaceutical Chemistry, Institute of Pharmaceutical Sciences, University of Graz, Schubertstraße 1, A-8010 Graz, Austria; 2 Institute for Chemistry and Technology of Materials (ICTM), Graz University of Technology, Stremayrgasse 9, A-8010 Graz, Austria; 3 Inorganic Chemistry, Institute of Chemistry, University of Graz, Schubertstraße 1, A-8010 Graz, Austria; 4 Swiss Tropical and Public Health Institute, Kreuzstraße 2, CH-4123 Allschwil, Switzerland; 5 Swiss Tropical and Public Health Institute, University of Basel, Petersplatz 1, CH-4003 Basel, Switzerland

**Keywords:** antimalarial, antitrypanosomal, azabicyclo-nonanes, *Plasmodium falciparum*, tetrazoles, *Trypanosoma brucei*

## Abstract

*N*-(Aminoalkyl)azabicyclo[3.2.2]nonanes possess antiplasmodial and antitrypanosomal activity. A series with terminal tetrazole or sulfonamido partial structure was prepared. The structures of all new compounds were confirmed by NMR and IR spectroscopy and by mass spectral data. A single crystal structure analysis enabled the distinction between isomers. The antiprotozoal activities were examined in vitro against strains of *Plasmodium falciparum* and *Trypanosoma brucei rhodesiense* (STIB 900). The most active sulfonamide and tetrazole derivates showed activities in the submicromolar range.

## 1. Introduction

Malaria and Human African Trypanosomiasis (HAT) both are tropical diseases transmitted to people by the bite of infected insects.

Malaria is caused by *Plasmodium* parasites. In 2020, there were about 241 million estimated cases of malaria and about 627 000 reported deaths [1]. There are five types of human malaria parasites, but the majority of infections are caused by *P. falciparum*, the most deadly malaria parasite [2]. Many strains of *P. falciparum* have become resistant to previous generations of medicines [1]. In recent years, resistance even to recommended artemisinin-based therapies has become prevalent across an expanding area of Southeast Asia [3,4,5,6]. Therefore, the development of new drugs for the fight against the most deadly strains of *P. falciparum* is absolutely essential.

HAT, also known as sleeping sickness, is caused by *Trypanosoma* parasites. After continued control efforts, the number of reported cases dropped below 1000 in 2019 [7]. However, transmitted by the tsetse fly, it puts 55 million people at risk and is fatal if untreated [8]. In the case of *T. b. rhodesiense* infections, the disease is acute, lasting from a few weeks to several months, while in case of *T. b. gambiense* infections, the disease is chronic, generally lasting several years without any major signs or symptoms. Sleeping sickness is difficult to treat considering the toxicity and complex administration of the drugs currently in use. Only a few drugs are available for the therapy of HAT: pentamidine, suramin, melarsoprol, nifurtimox, eflornithine and fexinidazole. For the treatment of *T. b. rhodesiense* infections of the central nervous system, melarsoprol is the only effective drug [7]. Unfortunately, melarsoprol causes an encephalopathy that kills 5% of the patients [9]. Therefore, the development of new drugs against Human African Trypanosomiasis is still required.

**Figure 1:**
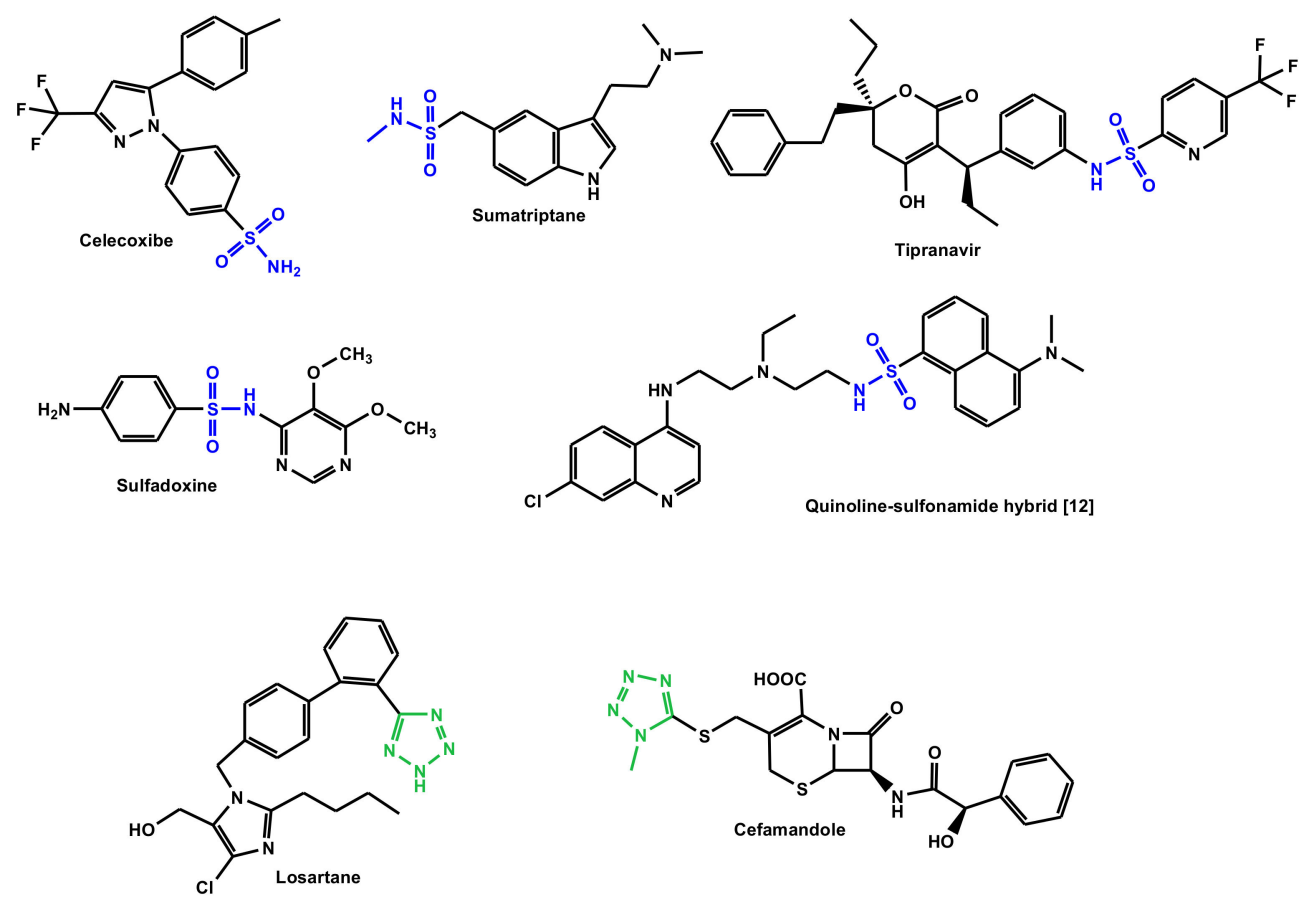
Drugs with sulfonamide or tetrazole partial structure used against inflammation (Celecoxibe), migraine (Sumatriptane), hypertonia (Losartane), in antiretroviral therapy (Tipranavir) and as antimicrobial agents (Sulfadoxine, Cefamandole, Quinoline–sulfonamide hybrid).

Submicromolar concentrations of some azabicyclo [3.2.2]nonanes with a dialkylamino substituent at a bridgehead atom showed activity against the K_1_ strain of *P. falciparum* and *T. b. rhodesiense* [10,11]. This paper examines the preparation of derivates with additional tetrazole or sulfonamide cores. Recently, we reported the synthesis and antiprotozoal activities of a series of 4-aminoquinoline derivates with a tetrazole ring at the terminal side chain [12]. As far as it is known, the tetrazole ring represents a very important structural unit in drug design and development [13], but its combination with an azabicyclo-nonane moiety has not yet been reported. Sulfonamide derivates have been widely used as pharmaceutical agents with a diverse range of biological and pharmaceutical activities. Sulfonamides are used against inflammation, migraine, in antiretroviral therapy and especially as antimicrobial agents. Sulfadoxine, for example, is used as standard medication together with pyrimethamine against *P. falciparum* [1]. Hybrids with quinoline and sulfonamide partial structure featuring antiplasmodial activity are described in literature [14] (Figure 1). A couple of 2-sulfonyl-2-azabicyclo[3.2.2]nonanes showed activity against the K_1_ strain of *P. falciparum* and *T. b. rhodesiense* [15].

This paper reports the synthesis and the antiprotozoal activities of azabicyclo[3.2.2]nonanes with terminal tetrazole or sulfonamido partial structure. The structures of all newly synthesized compounds were elucidated by 1D- and 2D-NMR spectroscopy. Their activities against strains of *P. falciparum* and against *T. b. rhodesiense* were investigated in vitro and the results compared to those of formerly prepared analogues and of drugs in use.

## 2. Results

### 2.1. Chemistry

**Scheme 1:**
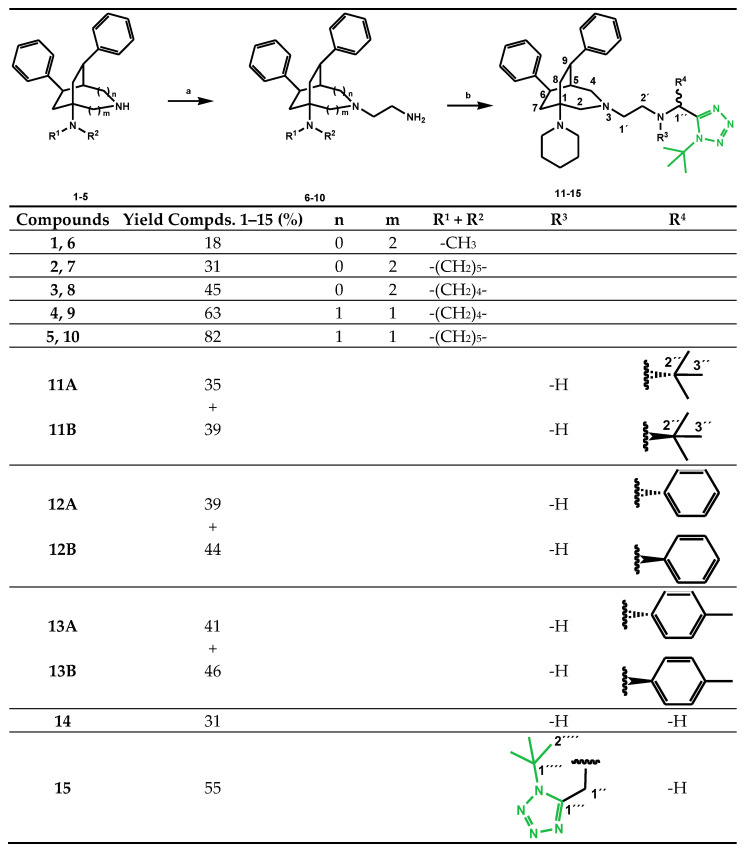
Preparation of compounds **6**–**15**. Reagents and conditions: (a) 1. EtOH abs., 2-chloroacetamide, 100 °C, 48 h; 2. LiAlH_4_, diethyl ether, 55 °C, 20 h; (b) aldehyde, *tert*-butylisocyanide, trimethylsilylazide, MeOH abs., 20 h, 20 °C.

The syntheses of 2-azabicyclo[3.2.2]nonanes **1**–**3** and 3-azabicyclo[3.2.2]nonanes **4**, **5** as starting materials were already described elsewhere [10,11]. They were refluxed with 2-chloroacetamide in EtOH yielding their carbamoylmethyl derivates, which were hydrogenated using LiAlH_4_, yielding the corresponding *N*-(2-aminoalkyl) analogues **6**–**10**. Afterward, **6**–**10** were converted to tetrazoles **11**–**15** in moderate to good yields at mild conditions via the Ugi-azide reaction with diverse aldehydes, *tert*-butylisocyanide and trimethylsilylazide (Scheme 1).

**Scheme 2:**
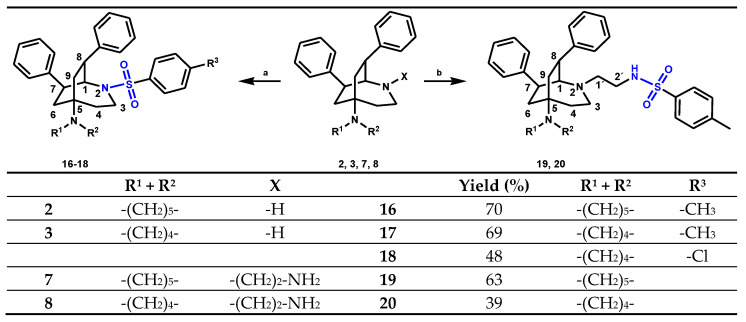
Preparation of compounds **16**–**20**. Reagents and conditions: (a) Arylsulfonyl chloride, 4-DMAP, CH_2_Cl_2_ abs., 20 h, 50 °C; (b) Arylsulfonyl chloride, 4-DMAP or DIPEA, CH_2_Cl_2_ abs., 20 h, 50 °C.

The preparation of sulfonyl derivates **16**–**18** succeeded by reaction of 2-azabicyclo-nonanes **2**, **3** with the corresponding arylsulfonyl chloride in the presence of 4-DMAP. Sulfonamides **19**, **20** were obtained from 2-(2-azabicyclo-nonan-2-yl)ethan-1-amines **7**, **8** (Scheme 2).

**Figure 2:**
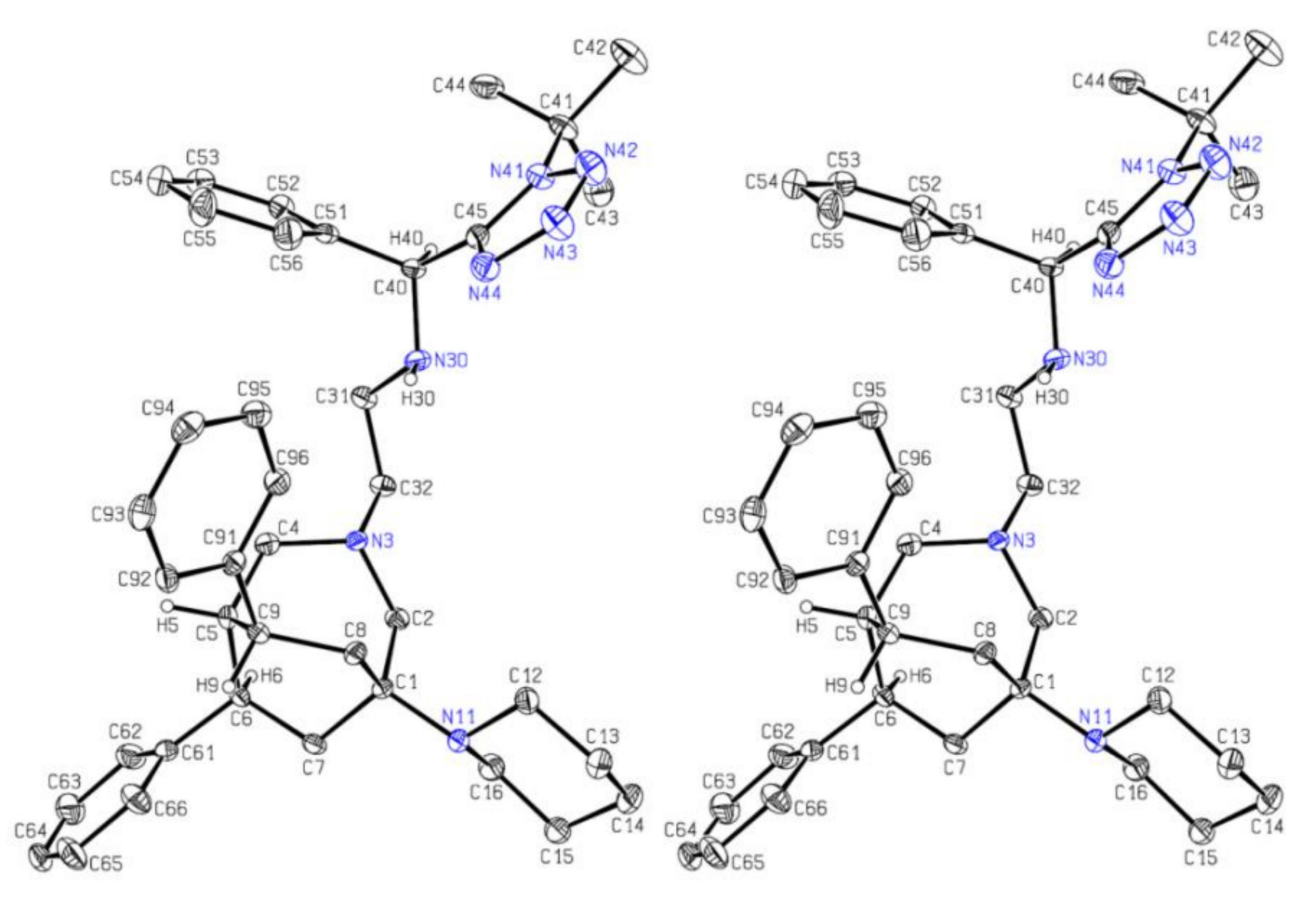
Stereoscopic ORTEP [16] plot of **12B** showing the atomic numbering scheme. The probability ellipsoids are drawn at the 50% probability level.

The structures of all newly synthesized compounds were clarified by one- and two-dimensional NMR spectroscopy. Successful alkylation of the ring nitrogen atom of 2-azabicyclononanes was obvious from 7 ppm downfield shifts of the ^13^C resonances of the adjacent ring atoms C-1 and C-3 of compounds **6**–**8**. The same effect was observed in the 3-azabicyclononane series for the signals of the corresponding C-2 and C-4 atoms of compounds **9**, **10**. The sulfonylation of the ring nitrogen atom of the 2-azabicyclononane **3** led to small downfield shifts < 2 ppm of the resonances of C-1 and C-3 of **18**; however, remarkable 0.5–1.0 ppm downfield shifts were observed for the signals of 1-H and 3-H in its proton nmr spectrum. Similarly, the sulfonylation of the ethanamine nitrogen of compounds **7**, **8** shifted the resonances of the 2’-H and C-2’ slightly to higher frequencies, which were detected in the spectra of sulfonamides **19**, **20**. The formation of the tetrazole derivates **11**–**15** was established by a number of changes in ^1^H and ^13^C nmr spectra. The *N*-alkylation of **9**, **10** caused a downfield shift of 6-7 ppm for the C-2’ signal. Moreover, we observed additional resonances for the newly introduced protons and carbons of the C-1 chain, the tetrazole moiety and their substituents. Evidence of structure was provided by the expected long-range couplings from 1’’-H to C-2’ and C-1’’’, which were detected in the HMBC spectra of compounds **11**–**15**. The distinction between isomers **11**–**13** was enabled by a single crystal structure analysis of compound **12B** (Figure 2). The crystal structure analysis of **12B** confirmed the compound as *N*-[(1-*tert*-butyl-1*H*-tetrazol-5-yl)(phenyl)methyl]-2-[6,9-diphenyl-1-(piperidin-1-yl)-3-azabicyclo[3.2.2]nonan-3-yl]eth-an-1-amine. All atoms lie on general positions. Owing to the absence of heavier elements, the absolute structure of the chiral molecule could not be determined reliably from the diffraction data. Arbitrarily, the structure is described as the *R*,*R*,*S* enantiomer (Figure 1). The tetrazole ring has adopted the orientation where the distance from N44 to the H atom of the NH group is smallest [N30···N44 2.841(4) Å]. Presumably, the intramolecular hydrogen bond is weak because of the small angle N30–H30···N44 of 104(2)°. CCDC 2,194,079 contains the supplementary crystallographic data for this paper. These data can be obtained free of charge via http://www.ccdc.cam.ac.uk/conts/retrieving.html (accessed on 16 August 2022), (or from the CCDC, 12 Union Road, Cambridge CB2 1EZ, UK; Fax: +44-1223-336033; E-mail: deposit@ccdc.cam.ac.uk).

### 2.2. Antiprotozoal Activity

Compounds were tested for their activities against strains of *P. falciparum* and *T. b. rhodesiense* via microplate assays. Their cytotoxicity was determined with rat skeletal myoblasts (L-6 cells). Chloroquine and melarsoprol served as standards (Table 1). 

The formerly synthesized 2-[(4-methylphenyl)sulfonyl]-2-azabicyclo[3.2.2]nonanes **16**, **17** showed antiplasmodial activity against *P.f.* K_1_ (*P.f.* K_1_ IC_50_ = 1.26; 1.30 µM) and antitrypanosomal (IC_50_ = 2.65; 1.26 µM) activity in the low micromolar range [15]. Replacement of the 4-methyl by a 4-chloro substituent and the insertion of an aminomethyl linker between the bridged ring system and the sulfonyl group led to compounds **18**–**20** with slightly improved antitrypanosomal activities (*T. b. r.* IC_50_ = 0.647–1.19 µM). Moreover, compounds **18**–**20** showed antiplasmodial activities against a sensitive strain in submicromolar concentration (*P.f.* NF54 IC_50_ = 0.487–0.606 µM). Their selectivities (SI ≤ 18.7) were only moderate, unfortunately. Similarly, tetrazole derivates **11**–**15** showed activity against *P. falciparum* NF54 (IC_50_ = 0.252–2.98 µM) and *T. brucei rhodesiense* (IC_50_ = 0.329–6.61 µM) in the low micromolar range, but selectivity indices (SI ≤ 20.1) were unfavorable due to their comparably high cytotoxicity. The intermediates **6**–**9** showed similar antiplasmodial activity against the multiresistant strain but distinctly improved selectivity (SI = 14.5–93.3 µM). The most promising compound of this series was **7** exhibiting submicromolar antiplasmodial activity (*P.f.* K_1_ IC_50_ = 0.180 µM) and quite good selectivity (SI = 93.3 µM). 

## 3. Conclusions

The synthesis and the antiprotozoal activities of a series of azabicyclo[3.2.2]nonane derivates with terminal tetrazole or sulfonamido partial structure were reported. The most active tetrazoles (12A: *P.f.* NF54 IC_50_ = 0.252 µM; 13A: *T. b. r.* IC_50_ = 0.329 µM) and sulfonamides (20: *P.f.* NF54 IC_50_ = 0.487 µM; *T. b. r.* IC_50_ = 0.647 µM) showed antiprotozoal activity in the submicromolar range. However, their selectivities were not satisfactory due to their relatively high cytotoxicity. The most promising of the tested compounds was the 2-(2-azabicyclononan-2-yl)ethan-1-amine 7, which had good antiplasmodial activity (*P.f.* K_1_ IC_50_ = 0.180 µM) and selectivity (SI = 93.3). 

## 4. Materials and Methods

### 4.1. Instrumentation and Chemicals

Materials: Solvents and reagents were used without additional purification. Dry solvents were either purchased in sealed bottles or were dried over molecular sieves or with sodium. Column chromatography (CC): silica gel 60 (Merck 70–230 mesh, pore diameter 60 Å), aluminum oxide (pH: 9.5, Fluka). Thin-layer chromatography (TLC): TLC plates silica gel 60 F254 (Merck), aluminum oxide 60 F254 (neutral, Merck). Melting points were obtained on an Electrothermal IA 9200 melting point apparatus. IR spectra: Bruker Alpha Platinum ATR FTIR spectrometer (KBr discs); frequencies are reported in cm^−1^. The structures of all newly synthesized compounds were determined by one- and two-dimensional NMR spectroscopy. NMR spectra: Varian UnityInova 400 (298 K) 5 mm tubes, TMS as internal standard. Shifts in ^1^H NMR (400 MHz) and ^13^C NMR (100 MHz) spectra are reported in ppm; ^1^H- and ^13^C-resonances were assigned using ^1^H,^1^H- and ^1^H,^13^C-correlation spectra and are numbered as given in Scheme 1 for 3-azabicyclo-nonanes and Scheme 2 for 2-azabicyclo-nonanes. Signal multiplicities are abbreviated as follows: br, broad; d, doublet; dd, doublet of doublets; ddd, doublet of doublet of doublets; dt, doublet of triplets; m, multiplet; s, singlet; t, triplet. HRMS: Micromass Tofspec 3E spectrometer (MALDI) and GCT-Premier, Waters (EI, 70eV). ^1^H-NMR and ^13^C-NMR spectra of new compounds are available in the Supplementary Materials (Figures S1–S16).

### 4.2. Syntheses

The syntheses of bridged heterocycles **1**–**5** have already been reported elsewhere [10,11].

#### 4.2.1. General Procedure for the Synthesis of *rac*-(7R,8R)-2-[5-(dialkylamino)-7,8-diphenyl-2-azabicyclo[3.2.2]nonan-2-yl]ethan-1-amines 6–8 and *rac*-(6R,9R)-2-[1-(dialkylamino)-6, 9-diphenyl-3-azabicyclo[3.2.2]nonan-3-yl]ethan-1-amines **9** and **10**

The bicyclononane **1**–**5** was dissolved in 20 mL dry ethanol and cooled on an ice bath with stirring under an atmosphere of argon. A solution of chloroacetamide in 12 mL dry ethanol was added. The mixture was refluxed for 48 h at 100 °C, allowed to cool to room temperature, diluted with water and alkalized with 2*N* NaOH. It was then extracted 4 times with diethyl ether. The combined organic phases were washed with water, dried over anhydrous sodium sulfate, filtered and finally the solvent was removed in vacuo. The obtained crude products were purified, if necessary, by column chromatography giving the corresponding 2-substituted acetamide derivate as colorless resin. It was suspended in 20 mL dry diethyl ether under stirring and cooling in an ice bath. LiAlH_4_ was added in portions and the mixture was refluxed overnight. The reaction was cautiously quenched with ice water; 2*N* NaOH was added and the mixture was extracted 5 times with CH_2_Cl_2_; the combined organic phases were washed twice with water, dried over anhydrous sodium sulfate, filtered and finally the solvent was removed in vacuo giving colorless oils.

##### *rac*-(7R,8R)-2-[5-(Dimethylamino)-7,8-diphenyl-2-azabicyclo[3.2.2]nonan-2-yl]ethan-1-amine (**6**)

The reaction of 0.932 g 2-azabicyclo-nonane **1** (2.91 mmol) and 0.272 g chloroacetamide (2.91 mmol) gave after 48 h a crude product. It was suspended in dry diethyl ether and reacted with 0.270 g LiAlH_4_ (7.115 mmol) to a residue which was purified by multiple column chromatography (first time on neutral aluminum oxide, CH_2_Cl_2_/MeOH = 29 + 1; second time on neutral aluminum oxide, CH_2_Cl_2_/MeOH = 9 + 1; and finally CH_2_Cl_2_/MeOH = 1 + 1) yielding 0.056 g **6** (18%) as colorless oil. IR = 3024, 2930, 2823, 2780, 1600, 1494, 1450, 1154, 1093, 1034, 757, 700; UV (CH_2_Cl_2_, (log ε)): 233.5 (3.848); ^1^H NMR (CDCl_3_, 400 MHz): δ = 1.90–1.95 (m, 2H, 4-H), 2.02 (dd, *J* = 13.5, 7.7 Hz, 1H, 6-H), 2.12–2.26 (m, 2H, 2’-H, 9-H), 2.29–2.39 (m, 3H, 2’-H, 6-H, 9-H), 2.37 (s, 6H, N(CH_3_)_2_), 2.44–2.50 (m, 2H, 1’-H), 2.72 (d, *J* = 3.5 Hz, 1H, 1-H), 2.75–2.82 (m, 1H, 3-H), 2.96 (dt, *J* = 13.0, 5.6 Hz, 1H, 3-H), 3.26 (ddd, *J* = 11.0, 7.4, 3.5 Hz, 1H, 8-H), 3.45 (dd, *J* = 10.3, 7.7 Hz, 1H, 7-H), 7.14–7.36 (m, 10H, aromatic H); ^13^C NMR (CDCl_3_, 100 MHz): δ = 31.30 (C-4), 34.67 (C-6), 36.33 (C-9), 37.78 (C-8), 37.95 (N(CH_3_)_2_), 39.59 (C-2’), 39.75 (C-7), 48.58 (C-3), 58.06 (C-5), 59.18 (C-1’), 69.00 (C-1), 126.19, 126.23, 127.55, 128.10, 128.70, 128.72 (aromatic C), 144.56, 145.70 (aromatic C_q_). HRMS (EI+) calcd for C_24_H_33_N_3_: 363.2675; found: 363.2685.

##### *rac*-(7R,8R)-2-[7,8-Diphenyl-5-(piperidin-1-yl)-2-azabicyclo[3.2.2]nonan-2-yl]ethan-1-amine (**7**)

The reaction of 0.200 g 2-azabicyclo-nonane **2** (0.556 mmol) and 0.052 g chloroacetamide (0.556 mmol) gave after 48 h a crude product. It was suspended in dry diethyl ether and reacted with 0.073 g LiAlH_4_ (1.912 mmol) to a residue, which was purified by multiple column chromatography (first time on neutral aluminum oxide, CH_2_Cl_2_/MeOH = 49 + 1; and finally on neutral aluminum oxide CH_2_Cl_2_/MeOH = 1 + 1) yielding 0.038 g **7** (31%) as colorless oil. IR = 3024, 2927, 2852, 1600, 1494, 1451, 1153, 1098, 1031, 757, 700; UV (CH_2_Cl_2_, (log ε)): 232.5 (3.881); ^1^H NMR (CDCl_3_, 400 MHz): δ = 1.44–1.52 (m, 2H, CH_2_), 1.62–1.71 (m, 4H, 2CH_2_), 1.93–2.07 (m, 3H, 4-H, 6-H), 2.16–2.48 (m, 7H, 1’-H, 2’-H, 6-H, 9-H), 2.63–2.73 (m, 4H, N(CH_2_)_2_), 2.77 (d, *J* = 2.9 Hz, 1H, 1-H), 2.77–2.82 (m, 1H, 3-H), 2.97 (dt, *J* = 13.2, 5.7 Hz, 1H, 3-H), 3.25 (ddd, *J* = 10.9, 7.7, 3.2 Hz, 1H, 8-H), 3.43 (br t, *J* = 9.1 Hz, 1H, 7-H), 7.15–7.38 (m, 10H, aromatic H); ^13^C NMR (CDCl_3_, 100 MHz): δ = 24.78 (CH_2_), 26.42 (2CH_2_), 32.62 (C-4), 34.48 (C-6), 35.99 (C-9), 38.17 (C-8), 39.63 (C-2’), 40.37 (C-7), 46.28 (N(CH_2_)_2_), 48.50 (C-3), 58.95 (C-5), 59.34 (C-1’), 68.86 (C-1), 126.08, 126.14, 127.49, 128.03, 128.54, 128.64 (aromatic C), 144.51, 145.72 (aromatic C_q_). HRMS (EI+) calcd for C_27_H_37_N_3_: 403.2987; found: 403.2996.

##### *rac*-(7R,8R)-2-[7,8-Diphenyl-5-(pyrrolidin-1-yl)-2-azabicyclo[3.2.2]nonan-2-yl]ethan-1-amine (**8**)

The reaction of 0.270 g 2-azabicyclo-nonane **3** (0.750 mmol) and 0.070 g chloroacetamide (0.750 mmol) gave after 48 h a crude product. It was suspended in dry diethyl ether and reacted with 0.119 g LiAlH_4_ (3.135 mmol), yielding 0.118 g **8** (45%) as colorless oil. IR = 3024, 2926, 2854, 1600, 1494, 1450, 1116, 1031, 755, 700; UV (CH_2_Cl_2_, (log ε)): 232.5 (3.838); ^1^H NMR (CDCl_3_, 400 MHz): δ = 1.74–1.81 (m, 4H, (CH_2_)_2_), 1.95–2.01 (m, 2H, 4-H), 2.08 (dd, *J* = 13.8, 8.0 Hz, 1H, 6-H), 2.16–2.38 (m, 5H, 2’-H, 6-H, 9-H), 2.42 (t, *J* = 5.4 Hz, 2H, 1’-H), 2.74–2.83 (m, 6H, 1-H, 3-H, N(CH_2_)_2_), 2.95 (dt, *J* = 12.8, 5.5 Hz, 1H, 3-H), 3.25 (ddd, *J* = 10.9, 7.6, 3.3 Hz, 1H, 8-H), 3.45 (dd, *J* = 9.7, 8.3 Hz, 1H, 7-H), 7.14–7.39 (m, 10H, aromatic H); ^13^C NMR (CDCl_3_, 100 MHz): δ = 23.58 ((CH_2_)_2_), 33.20 (C-4), 35.52 (C-6), 36.64 (C-9), 37.83 (C-8), 39.59 (C-2’), 39.79 (C-7), 45.10 (N(CH_2_)_2_), 48.63 (C-3), 56.71 (C-5), 60.06 (C-1’), 69.01 (C-1), 125.83, 125.97, 127.50, 127.79, 128.52, 128.68 (aromatic C), 144.64, 146.00 (aromatic C_q_). HRMS (EI+) calcd for C_26_H_35_N_3_: 389.2831; found: 389.2837.

##### *rac*-(6R,9R)-2-[6,9-Diphenyl-1-(pyrrolidin-1-yl)-3-azabicyclo[3.2.2]nonan-3-yl]ethan-1-amine (**9**)

The reaction of 0.874 g 3-azabicyclo-nonane **4** (2.5 mmol) and 0.236 g chloroacetamide (2.5 mmol) gave after 48 h a crude product. It was suspended in dry diethyl ether and reacted with 0.300 g LiAlH_4_ (7.9 mmol), yielding 0.625 g **9** (63%) as colorless oil. IR = 3420, 2953, 2624, 2484, 1600, 1496, 1452, 1031, 753, 702; ^1^H NMR (CDCl_3_, 400 MHz): δ = 1.76 (br s, 4H, (CH_2_)_2_), 1.87–2.05 (m, 3H, 5-H, 7-H, 8-H), 2.29–2.35 (m, 2H, 4-H, 7-H), 2.49–2.61 (m, 2H, 1’-H), 2.63–2.90 (m, 8H, 2-H, 8-H, N(CH_2_)_2_, 2’-H), 2.97–3.03 (m, 2H, 2-H, 4-H), 3.30–3.36 (m, 2H, 6-H, 9-H), 7.16–7.42 (m, 10H, aromatic H); ^13^C NMR (CDCl_3_, 100 MHz): δ = 23.52 ((CH_2_)_2_), 34.84 (C-8), 38.04 (C-7), 38.81 (C-9), 39.39 (C-2’), 44.56 (C-5), 44.91 (C-6), 45.32 (N(CH_2_)_2_), 58.03 (C-1), 58.78 (C-2), 60.72 (C-4), 61.12 (C-1’), 126.01, 126.11, 126.87, 128.17, 128.27, 128.54 (aromatic C), 144.92, 146.64 (aromatic C_q_); HRMS (EI+): calcd for C_26_H_35_N_3_: 389.2831; found: 389.2834. 

##### *rac*-(6R,9R)-2-[6,9-Diphenyl-1-(piperidin-1-yl)-3-azabicyclo[3.2.2]nonan-3-yl]ethan-1-amine (**10**)

The reaction of 1.14 g 3-azabicyclo-nonane **5** (3.16 mmol) and 0.295 g chloroacetamide (3.16 mmol) gave after 48 h a crude product. It was suspended in dry diethyl ether and reacted with 0.413 g LiAlH_4_ (10.88 mmol), yielding 0.80 g **10** (82%) as colorless oil. ^1^H NMR (CDCl_3_) δ = 1.39–1.48 (m, 2H, CH_2_), 1.54–1.64 (m, 4H, 2CH_2_), 1.84–1.94 (m, 2H, 7-H, 8-H), 1.96–2.01 (m, 1H, 5-H), 2.24–2.29 (m, 1H, 7-H), 2.31 (d, *J* = 11.5 Hz, 1H, 4-H), 2.49–2.65 (m, 8H, 1’-H, 2-H, 8-H, N(CH_2_)_2_), 2.77–2.91 (m, 2H, 2’-H), 2.95 (d, *J* = 11.8 Hz, 1H, 2-H), 3.01 (dd, *J* = 11.4, 5.2 Hz, 1H, 4-H), 3.26–3.31 (m, 2H, 6-H, 9-H), 7.15–7.42 (m, 10H, aromatic H); ^13^C NMR (CDCl_3_) δ = 25.03 (CH_2_), 26.74 (2CH_2_), 34.04 (C-8), 37.39 (C-7), 39.24 (C-9), 39.48 (C-2’), 44.18 (C-5), 45.01 (C-6), 46.52 (N(CH_2_)_2_), 58.60 (C-2), 59.81 (C-1), 60.80 (C-4), 61.22 (C-1’), 126.05, 126.13, 126.80, 128.20, 128.30, 128.56 (aromatic C), 145.04, 146.70 (aromatic C_q_); HRMS (EI+) calcd for C_27_H_37_N_3_: 403.2987; found: 403.2979.

#### 4.2.2. General Procedure for the Synthesis of N-[(1-tert-butyl-1H-tetrazol-5-yl)methyl]-2-[6,9-diphenyl-1-(piperidin-1-yl)-3-azabicyclo[3.2.2]nonan-3-yl]ethan-1-amines **11**–**15**

2-(3-Azabicyclononan-3-yl)ethan-1-amine **10** was dissolved in dry MeOH in an atmosphere of Ar. The corresponding aldehyde was added and the reaction batch was stirred for 1 h at ambient temperature. Subsequently, *tert*-butylisocyanide and trimethylsilylazide were added and the reaction was stirred for an additional 20 h at ambient temperature in an atmosphere of Ar. Subsequently, the solvent was evaporated in vacuo yielding crude products **11**–**15,** which were separated by column chromatography into their isomers (silica, diethyl ether/dioxane/MeOH = 25 + 1 + 1).

##### *rac*-(1R)-1-(1-tert-Butyl-1H-tetrazol-5-yl)-2,2-dimethyl-N-{2-[(6R,9R)-6,9-diphenyl-1-(piperidin-1-yl)-3-azabicyclo[3.2.2]nonan-3-yl]ethyl}propan-1-amine (**11A**) and *rac*-(1R)-1-(1-tert-Butyl-1H-tetrazol-5-yl)-2,2-dimethyl-N-{2-[(6S,9S)-6,9-diphenyl-1-(piperidin-1-yl)-3-azabicyclo[3.2.2]nonan-3-yl]ethyl}propan-1-amine (**11B**)

The reaction batch of 0.330 g 2-(3-azabicyclononan-3-yl)ethan-1-amine **10** (0.81 mmol), 0.079 g trimethylacetaldehyde (0.930 mmol), 0.072 g *tert*-butylisocyanide (0.830 mmol) and 0.104 g trimethylsilylazide (0.870 mmol) in 4.5 mL dry MeOH was stirred for 20 h. Subsequently, the solvent was evaporated in vacuo. Solvent chromatography gave 0.171 g (35%) of **11A** and 0.191 g (39%) of **11B**.

**11A**: IR = 2933, 1601, 1495, 1451, 1392, 1297, 1241, 1103, 1031, 909, 814, 744, 699; ^1^H NMR (CDCl_3_, 400 MHz): δ = 1.09 (s, 9H, 3’’-H), 1.39–1.47 (m, 2H, CH_2_), 1.51–1.62 (m, 4H, 2CH_2_), 1.80 (s, 9H, 2’’’’-H), 1.80–1.94 (m, 3H, 5-H, 7-H, 8-H), 2.16–2.24 (m, 1H, 7-H), 2.30 (d, *J* = 11.4 Hz, 1H, 4-H), 2.40–2.65 (m, 10H, 1’-H, 2-H, 2’-H, N(CH_2_)_2_), 2.84 (d, *J* = 12.8 Hz, 1H, 2-H), 2.89 (dd, *J* = 11.4, 4.8 Hz, 1H, 4-H), 3.21–3.30 (m, 2H, 6-H, 9-H), 4.02 (s, 1H, 1’’-H), 7.17 (t, *J* = 7.3 Hz, 1H, aromatic H), 7.19–7.44 (m, 9H, aromatic H); ^13^C NMR (CDCl_3_, 100 MHz): δ = 25.04 (CH_2_), 26.70 (2CH_2_), 27.21 (C-3’’), 31.41 (C-2’’’’), 33.71 (C-8), 36.29 (C-2’’), 37.66 (C-7), 39.34 (C-9), 44.42 (C-5), 44.83 (C-6), 46.39 (C-2’), 46.50 (N(CH_2_)_2_), 57.83 (C-2), 57.97 (C-1’), 59.50 (C-1), 61.47 (C-4), 62.01 (C-1’’’’), 62.45 (C-1’’), 125.90, 126.05, 126.84, 128.17, 128.51, 128.54 (aromatic C), 145.13, 146.79 (aromatic C_q_), 157.25 (C-1’’’); HRMS (EI+) calcd for C_37_H_55_N_7_: 597.4519; found: 597.4533. 

**11B**: IR = 2932, 2793, 1601, 1495, 1452, 1393, 1374, 1304, 1239, 1103, 1032, 911, 866, 811, 743, 699; ^1^H NMR (CDCl_3_, 400 MHz): δ = 1.07 (s, 9H, 3’’-H), 1.40–1.48 (m, 2H, CH_2_), 1.52–1.61 (m, 4H, 2CH_2_), 1.79 (s, 9H, 2’’’’-H), 1.80–1.89 (m, 2H, 7-H, 8-H), 1.91 (br s, 1H, 5-H), 2.20–2.31 (m, 2H, 4-H, 7-H), 2.36–2.42 (m, 1H, 2’-H), 2.47–2.63 (m, 9-H, 1’-H, 2-H, 2’-H, 8-H, N(CH_2_)_2_), 2.90 (d, *J* = 12.1 Hz, 1H, 2-H), 2.88–2.94 (m, 1H, 4-H), 3.19–3.30 (m, 2H, 6-H, 9-H), 4.00 (s, 1H, 1’’-H), 7.17 (d, *J* = 7.2 Hz, 1H, aromatic H), 7.19–7.40 (m, 9H, aromatic H); ^13^C NMR (CDCl_3_, 100 MHz): δ = 25.06 (CH_2_), 26.75 (2CH_2_), 27.24 (C-3’’), 31.38 (C-2’’’’), 34.18 (C-8), 36.18 (C-2’’), 37.58 (C-7), 39.48 (C-9), 44.14 (C-5), 44.79 (C-6), 46.58 (N(CH_2_)_2_), 46.67 (C-2’), 58.14 (C-1’), 58.33 (C-2), 59.67 (C-1), 60.74 (C-4), 62.15 (C-1’’’’), 62.60 (C-1’’), 126.01, 126.07, 126.79, 128.17, 128.52 (aromatic C), 145.18, 146.74 (aromatic C_q_), 157.32 (C-1’’’); HRMS (EI+) C_37_H_55_N_7_: 597.4519; found: 597.4554. 

##### *rac*-N-[(1R)-(1-tert-Butyl-1H-tetrazol-5-yl])(phenyl)methyl]-2-[(6R,9R)-6,9-diphenyl-1-(piperidin-1-yl)-3-azabicyclo[3.2.2]nonan-3-yl]ethan-1-amine (**12A**) and *rac*-N-[(1R)-1-tert-Butyl-1H-tetrazol-5-yl])(phenyl)methyl]-2-[(6S,9S)-6,9-diphenyl-1-(piperidin-1-yl)-3-azabicyclo[3.2.2]nonan-3-yl]ethan-1-amine (**12B**)

The reaction batch of 0.371 g 2-(3-azabicyclononan-3-yl)ethan-1-amine **10** (0.920 mmol), 0.079 g benzaldehyde (0.920 mmol), 0.099 g *tert*-butylisocyanide (1.21 mmol) and 0.143 g trimethylsilylazide (1.21 mmol) in 4.5 mL dry MeOH was stirred for 20 h. Subsequently, the solvent was evaporated in vacuo. Solvent chromatography gave 0.221 g (39%) of **12A** and 0.250 g of **12B** (44%). 

**12A**: IR = 2931, 1600, 1494, 1452, 1374, 1236, 1105, 1030, 851, 744, 700; ^1^H NMR (CDCl_3_, 400 MHz): δ = 1.38–1.44 (m, 2H, CH_2_), 1.50–1.57 (m, 4H, 2CH_2_), 1.63 (s, 9H, 2’’’’-H), 1.79–1.89 (m, 2H, 7-H, 8-H), 1.93 (br s, 1H, 5-H), 2.21–2.29 (m, 1H, 7-H), 2.31 (d, *J* = 11.4 Hz, 1H, 4-H), 2.44–2.78 (m, 10H, 1’-H, 2-H, 2’-H, 8-H, N(CH_2_)_2_), 2.87 (d, *J* = 12.6 Hz, 1H, 2-H), 2.93 (dd, *J* = 11.4, 5.1 Hz, 1H, 4-H), 3.21–3.29 (m, 2H, 6-H, 9-H), 5.31 (s, 1H, 1’’-H), 7.15 (t, *J* = 7.3 Hz, 1H, aromatic H), 7.18–7.41 (m, 14H, aromatic H); ^13^C NMR (CDCl_3_, 100 MHz): δ = 24.98 (CH_2_), 26.69 (2CH_2_), 29.99 (C-2’’’’), 34.05 (C-8), 37.43 (C-7), 39.24 (C-9), 44.33 (C-5), 44.82 (C-6), 45.28 (C-2’), 46.45 (N(CH_2_)_2_), 57.74 (C-1’), 58.16 (C-2), 59.31 (C-1’’), 59.53 (C-1), 61.02 (C-4), 61.26 (C-1’’’’), 126.03, 126.07, 126.79, 128.08, 128.26, 128.33, 128.41, 128.52, 128.96 (aromatic C), 138.58, 144.74, 146.68 (aromatic C_q_), 155.56 (C-1’’’); HRMS (EI+) calcd for C_39_H_51_N_7_: 617.4206; found: 617.4250.

**12B**: IR = 2930, 1672, 1601, 1494, 1452, 1374, 1237, 1105, 1031, 746, 700; ^1^H NMR (CDCl_3_, 400 MHz): δ = 1.41–1.49 (m, 2H, CH_2_), 154–1.62 (m, 4H, 2CH_2_), 1.65 (s, 9H, 2’’’’-H), 1.82–1.92 (m, 2H, 7-H, 8-H), 1.94 (br s, 1H, 5-H), 2.20–2.28 (m, 1H, 7-H), 2.31 (d, *J* = 11.4 Hz, 1H, 4-H), 2.54–2.67 (m, 9H, 1’-H, 2-H, 2’-H, 8-H, N(CH_2_)_2_), 2.70–2.79 (m, 1H, 2’-H), 2.87 (dd, *J* = 11.4, 5.0 Hz, 1H, 4-H), 2.93 (d, *J* = 12.5 Hz, 1H, 2-H), 3.22–3.28 (m, 1H, 9-H), 3.30 (d, *J* = 9.7 Hz, 1H, 6-H), 5.33 (s, 1H, 1’’-H), 7.11–7.40 (m, 15H, aromatic H); ^13^C NMR (CDCl_3_, 100 MHz): δ = 25.01 (CH_2_), 26.72 (2CH_2_), 30.00 (C-2’’’’), 33.87 (C-8), 37.39 (C-7), 39.09 (C-9), 44.28 (C-5), 44.75 (C-6), 45.57 (C-2’), 46.57 (N(CH_2_)_2_), 57.97 (C-1’), 58.98 (C-2), 59.26 (C-1’’), 59.60 (C-1), 60.56 (C-4), 61.17 (C-1’’’’), 125.88, 126.05, 126.83, 128.04, 128.14, 128.26, 128.35, 128.51, 128.95 (aromatic C), 138.81, 144.93, 146.76 (aromatic C_q_), 155.61 (C-1’’’); HRMS (EI+) calcd for C_39_H_51_N_7_: 617.4206; found: 617.4246. 

##### Crystal Structure Determination of **12B**

Crystals of **12B** (m.p.: 161.5–162.5 °C) for crystal structure analysis were obtained from a solution in ethyl acetate via slow evaporation of solvent. All the measurements were performed using monochromatized Mo K_a_ radiation at 100 K: C_39_H_51_N_7_, *M*_r_ 617.86, monoclinic, space group C 2, a = 28.755(2) Å, b = 6.5924(5) Å, c = 22.8112(16) Å, b = 128.182(8)°, V = 3399.0(5) Å^3^, Z = 4, d_calc_ = 1.207 g cm^−3^, m = 0.073 mm^−1^. A total of 26,894 reflections were collected (Q_max_ = 27.0°), from which 7256 were unique (R_int_ = 0.0772), with 5244 having I > 2s(I). The structure was solved by direct methods (SHELXS-97) [17] and refined by full-matrix least-squares techniques against *F*^2^ (SHELXL-2014/6) [18]. The nonhydrogen atoms were refined with anisotropic displacement parameters without any constraints. Owing to the absence of heavier elements, the absolute structure of the chiral molecule could not be determined reliably from the diffraction data and was chosen arbitrarily. The H atom bonded to N30 was taken from a difference Fourier map and refined without any positional constraints with an individual isotropic displacement parameter. The H atoms of the tertiary C–H groups were refined with individual isotropic displacement parameter and all X–C–H angles equal at a C–H distance of 1.00 Å. The H atoms of the CH_2_ groups were refined with common isotropic displacement parameters for the H atoms of the same group and idealized geometry with approximately tetrahedral angles and C–H distances of 0.99 Å. The H atoms of the phenyl rings were put at the external bisectors of the C–C–C angles at C–H distances of 0.95 Å, and common isotropic displacement parameters were refined for the H atoms of the same ring. The H atoms of the methyl groups were refined with common isotropic displacement parameters for the H atoms of the same group and idealized geometries with tetrahedral angles, enabling rotations around the C–C bonds, and C–H distances of 0.98 Å. For 443 parameters, final *R* indices of R^1^ = 0.0513 and wR^2^ = 0.1010 (GOF = 1.007) were obtained. The largest peak in a difference Fourier map was 0.215 e/Å^−3^

##### *rac*-N-[(1R)-(1-tert-Butyl-1H-tetrazol-5-yl)(p-tolyl)methyl]-2-[(6R,9R)-6,9-diphenyl-1-(piperidin-1-yl)-3-azabicyclo[3.2.2]nonan-3-yl]ethan-1-amine (**13A**) and *rac*-N-[(1R)-(1-tert-butyl-1H-tetrazol-5-yl)(p-tolyl)methyl]-2-[(6S,9S)-6,9-diphenyl-1-(piperidin-1-yl)-3-azabicyclo[3.2.2]nonan-3-yl]ethan-1-amine (**13B**)

The reaction batch of 0.398g 2-(3-azabicyclononan-3-yl)ethan-1-amine **10** (0.986 mmol), 0.118 g p-tolylaldehyde (0.982 mmol), 0.110 g *tert*-butylisocyanide (1.31 mmol) and 0.151 g trimethylsilylazide (1.31 mmol) in 4.5 mL dry MeOH was stirred for 20 h. Subsequently, the solvent was evaporated in vacuo. Solvent chromatography gave 0.255 g (41%) of **13A** and 0.286 g (46%) of **13B**. 

**13A**: IR = 2928, 1653, 1601, 1494, 1452, 1375, 1237, 1105, 1031, 862, 793, 745, 700; ^1^H NMR (CDCl_3_, 400 MHz): δ = 1.37–1.44 (m, 2H, CH_2_), 1.49–1.57 (m, 4H, 2CH_2_), 1.63 (s, 9H, 2’’’’-H), 1.80 (br t, *J* = 12.6 Hz, 1H, 8-H), 1.86 (br t, *J* = 12.8 Hz, 1H, 7-H), 1.92 (br s, 1H, 5-H), 2.20–2.31 (m, 2H, 4-H, 7-H), 2.31 (s, 3H, CH_3_), 2.44–2.54 (m, 5H, 2-H, N(CH_2_)_2_), 2.55–2.78 (m, 5H, 1’-H, 2’-H, 8-H), 2.86 (d, *J* = 12.6 Hz, 1H, 2-H), 2.93 (dd, *J* = 11.4, 4.8 Hz, 1H, 4-H), 3.21–3.29 (m, 2H, 6-H, 9-H), 5.27 (s, 1H, 1’’-H), 7.12–7.40 (m, 14H, aromatic H); ^13^C NMR (CDCl_3_, 100 MHz): δ = 21.06 (CH_3_), 25.01 (CH_2_), 26.72 (2CH_2_), 29.99 (C-2’’’’), 34.09 (C-8), 37.46 (C-7), 39.27 (C-9), 44.35 (C-5), 44.84 (C-6), 45.19 (C-2’), 46.44 (N(CH_2_)_2_), 57.74 (C-1’), 58.11 (C-2), 59.01 (C-1’’), 59.49 (C-1), 61.04 (C-4), 61.22 (C-1’’’’), 126.01, 126.06, 126.78, 127.96, 128.24, 128.42, 128.50, 129.61 (aromatic C), 135.57, 138.07, 144.75, 146.70 (aromatic C_q_), 155.69 (C-1’’’); HRMS (EI+) calcd for C_40_H_53_N_7_: 631.4362; found: 631.4405.

**13B**: IR = 2930, 1636, 1601, 1495, 1451, 1374, 1237, 1105, 1031, 803, 746, 699; ^1^H NMR (CDCl_3_, 400 MHz): δ = 1.40-1.49 (m, 2H, CH_2_), 1.52–1.63 (m, 4H, 2CH_2_), 1.64 (s, 9H, 2’’’’-H), 1.82–1.92 (m, 2H, 7-H, 8-H), 1.94 (br s, 1H, 5-H), 2.20–2.32 (m, 2H, 4-H, 7-H), 2.33 (s, 3H, CH_3_), 2.52–2.76 (m, 10H, 1’-H, 2-H, 2’-H, 8-H, N(CH_2_)_2_), 2.87 (dd, *J* = 11.4, 4.9 Hz, 1H, 4-H), 2.92 (d, *J* = 12.7 Hz, 1H, 2-H), 3.24 (br td, *J* = 9.8, 2.7 Hz, 1H, 9-H), 3.29 (t, *J* = 9.4 Hz, 1H, 6-H), 5.29 (s, 1H, 1’’-H), 7.12–7.40 (m, 14H, aromatic H); ^13^C NMR (CDCl_3_, 100 MHz): δ = 21.08 (CH_3_), 25.04 (CH_2_), 26.76 (2CH_2_), 30.03 (C-2’’’’), 33.96 (C-8), 37.38 (C-7), 39.14 (C-9), 44.29 (C-5), 44.74 (C-6), 45.53 (C-2’), 46.58 (N(CH_2_)_2_), 58.00 (C-1’), 58.98 (C-1’’), 59.04 (C-2), 59.63 (C-1), 60.50 (C-4), 61.15 (C-1’’’’), 125.89, 126.06, 126.85, 127.39, 127.94, 128.14, 128.39, 128.51, 129.60 (aromatic C), 135.82, 138.06, 144.97, 146.79 (aromatic C_q_), 155.76 (C-1’’’); HRMS (EI+) calcd for C_40_H_53_N_7_: 631.4362; found: 631.4387.

##### *rac*-(6R,9R)-N-[(1-tert-Butyl-1H-tetrazol-5-yl)methyl]-2-[6,9-diphenyl-1-(piperidin-1-yl)-3-azabicyclo[3.2.2]nonan-3-yl]ethan-1-amine (**14**) and *rac*-(6R,9R)-N,N-bis[(1-tert-Butyl-1H-tetrazol-5-yl)methyl]-2-[6,9-diphenyl-1-(piperidin-1-yl)-3-azabicyclo[3.2.2]nonan-3-yl]ethan-1-amine (**15**)

The reaction batch of 0.255g 2-(3-azabicyclononan-3-yl)ethan-1-amine **10** (0.632 mmol), 0.021 g paraformaldehyde (0.699 mmol), 0.071 g *tert*-butylisocyanide (0.844 mmol) and 0.097 g trimethylsilylazide (0.842 mmol) in 4.5 mL dry MeOH was stirred for 20 h. Subsequently, the solvent was evaporated in vacuo. Solvent chromatography gave 0.106 g of **14** (31%) and 0.130 mg of **15** (55%). 

**14**: IR = 2931, 1636, 1495, 1456, 1373, 1237, 1110, 1031, 911, 731, 700; ^1^H NMR (CDCl_3_, 400 MHz): δ = 1.42–1.50 (m, 2H, CH_2_), 1.55–1.65 (m, 4H, 2CH_2_), 1.77 (s, 9H, 2’’’’-H), 1.85–1.96 (m, 2H, 7-H, 8-H), 2.05 (br s, 1H, 5-H), 2.26–2.32 (m, 1H, 7-H), 2.32 (d, *J* = 11.3 Hz, 1H, 4-H), 2.55–2.75 (m, 9H, 1’-H, 2-H, 2’-H, 8-H, N(CH_2_)_2_), 2.78–2.86 (m, 1H, 2’-H), 2.97 (d, *J* = 12.6 Hz, 1H, 2-H), 3.01 (dd, *J* = 11.3, 5.1 Hz, 1H, 4-H), 3.26–3.33 (m, 2H, 6-H, 9-H), 4.13 (s, 2H, 1’’-H), 7.15 (t, *J* = 7.2 Hz, 1H, aromatic H), 7.20–7.40 (m, 9H, aromatic H); ^13^C NMR (CDCl_3_, 100 MHz): δ = 24.95 (CH_2_), 26.67 (2CH_2_), 29.55 (C-2’’’’), 33.95 (C-8), 37.14 (C-7), 38.74 (C-9), 43.86 (C-5), 44.44 (C-1’’), 44.95 (C-6), 46.53 (C-2’), 46.59 (N(CH_2_)_2_), 57.38 (C-1’), 58.76 (C-2), 59.73(C-1), 60.50 (C-4), 61.30 (C-1’’’’), 126.03, 126.19. 126.81, 128.08, 128.23, 128.60 (aromatic C), 144.79, 146.58 (aromatic C_q_), 152.92 (C-1’’’); HRMS (EI+) calcd for C_33_H_47_N_7_: 541.3893; found: 541.3937.

**15**: IR = 2933, 1635, 1453, 1374, 1234, 1106, 731, 700; ^1^H NMR (CDCl_3_, 400 MHz): δ = 1.41–1.49 (m, 2H, CH_2_), 1.52–1.64 (m, 4H, 2CH_2_), 1.66 (s, 18H, 2’’’’-H), 1.82–1.93 (m, 2H, 7-H, 8-H), 1.97 (br s, 1H, 5-H), 2.21–2.29 (m, 1H, 7-H), 2.33 (d, *J* = 11.4 Hz, 1H, 4-H), 2.47–2.64 (m, 5H, 8-H, N(CH_2_)_2_), 2.65–2.80 (m, 3H, 1’-H, 2-H), 2.93 (d, *J* = 12.8 Hz, 1H, 2-H), 2.97 (dd, *J* = 11.4, 4.9 Hz, 1H, 4-H), 3.14–3.21 (m, 2H, 2’-H), 3.21–3.30 (m, 2H, 6-H, 9-H), 4.39 (s, 4H, 1’’-H), 7.12–7.37 (m, 10H, aromatic H); ^13^C NMR (CDCl_3_, 100 MHz): δ = 24.87 (CH_2_), 26.63 (2CH_2_), 29.59 (C-2’’’’), 34.08 (C-8), 37.04 (C-7), 38.93 (C-9), 43.91 (C-5), 44.75 (C-6), 46.53 (N(CH_2_)_2_), 47.58 (C-1’’), 52.13 (C-2’), 55.71 (C-1’), 59.29 (C-2), 59.68 (C-1), 60.36 (C-4), 61.25 (C-1’’’’), 126.06, 126.16, 126.74, 128.12, 128.23, 128.55 (aromatic C), 144.77, 146.44 (aromatic C_q_), 151.38 (C-1’’’); HRMS (EI+) calcd for C_39_H_57_N_11_: 679.4799; found: 679.4833.

#### 4.2.3. General Procedure for the Synthesis of Benzenesulfonamides **18**–**20**

Azabicyclo-nonanes **3**, **7**, **8** were dissolved in dry CH_2_Cl_2_. Then, 4-DMAP or DIPEA and the respective aromatic sulfonyl chloride were added under stirring. The mixture was refluxed for 20 h at 50 °C. Subsequently, the reaction batch was shaken with 2 *N* aq NaOH, washed with water until the aqueous phase reacted neutral, dried over anhydrous sodium sulfate, filtered and finally the solvent was removed in vacuo giving crude products, which were purified by column chromatography yielding compounds **18**–**20**.

##### *rac*-(7R,8R)-2-(4-Chlorobenzenesulfonyl)-7,8-diphenyl-5-(pyrrolidin-1-yl)-2-azabicyclo[3.2.2]nonane (**18**)

The reaction of 0.333 g 2-azabicyclo-nonane **3** (0.96 mmol), 0.361 g 4-chlorobenzenesulfonyl chloride (1.71 mmol) and 1.92 g 4-DMAP (1.57 mmol) in 14 mL CH_2_Cl_2_ abs. gave a crude product, which was purified by column chromatography (aluminum oxide neutral, CH/EtAc = 3 + 1 and in addition 1% diethylamine) yielding 0.240 g **18** (48%) as colorless oil. IR = 2961, 2360, 1585, 1496, 1475, 1449, 1394, 1339, 1278, 1160, 1091, 1038, 1012, 974, 925, 864, 827, 760, 699; ^1^H NMR (CDCl_3_) δ = 1.74–1.82 (m, 4H, (CH_2_)_2_), 2.03–2.21 (m, 4H, 4-H, 6-H, 9-H), 2.24–2.36 (m, 2H, 6-H, 9-H), 2.66–2.79 (m, 4H, N(CH_2_)_2_), 3.26–3.44 (m, 3H, 3-H, 7-H, 8-H), 3.93 (dt, *J* = 13.7, 4.2 Hz, 1H, 3-H), 4.20 (d, *J* = 3.3 Hz, 1H, 1-H), 7.02 (d, *J* = 7.1 Hz, 2H, aromatic H), 7.07-7.41 (m, 10H, aromatic H), 7.47 (d, *J* = 7.5 Hz, 2H, aromatic H); ^13^C NMR (CDCl_3_) δ = 23.56 ((CH_2_)_2_), 31.97 (C-4), 33.85 (C-9), 36.67 (C-6), 38.30 (C-8), 42.41 (C-3), 45.23 (N(CH_2_)_2_), 46.73 (C-7), 56.43 (C-5), 63.62 (C-1), 126.54, 126.88, 127.53, 127.66, 127.97, 128.48, 128.81, 128.96 (aromatic C), 138.15, 139.03, 141.43, 143.46 (aromatic C_q_); HRMS (EI+) calcd for C_30_H_33_ClN_2_O_2_S: 520.1951; found: 520.1979.

##### *rac*-(7R,8R)-4-Methyl-N-[2-(7,8-diphenyl-5-(piperidin-1-yl)-2-azabicyclo[3.2.2]nonan -2-yl)ethyl]benzenesulfonamide (**19**)

The reaction of 0.143 g 2-azabicyclo-nonane **7** (0.35 mmol), 0.137 g toluene-4-sulfonyl chloride (0.72 mmol) and 0.118 g DIPEA (0.91 mmol) in 5 mL CH_2_Cl_2_ abs. gave a crude product, which was purified by column chromatography (aluminum oxide neutral, CH/EtAc = 5 + 1 → 1 + 1 finished by CH/EtAc/MeOH 1 + 1 + 0.1) yielding 0.120 g **19** (63%) as colorless oil. IR = 2929, 1635, 1600, 1495, 1450, 1330, 1160, 1093, 815, 756, 701, 665; ^1^H NMR (CDCl_3_) δ = 1.39–1.49 (m, 2H, CH_2_), 1.55–1.64 (m, 4H, 2CH_2_), 1.78–1.92 (m, 3H, 4-H, 6-H), 2.08 (br t, *J* = 12.1 Hz, 1H, 9-H), 2.19–2.32 (m, 3H, 1’-H, 6-H, 9-H), 2.36 (s, 3H, CH_3_), 2.37–2.44 (m, 1H, 1’-H), 2.48–2.63 (m, 6H, 2’-H, 3-H, N(CH_2_)_2_), 2.55 (d, *J* = 3.6 Hz, 1H, 1-H), 2.71-2.84 (m, 2H, 2’-H, 3-H), 3.23 (ddd, *J* = 11.4, 8.1, 3.4 Hz, 1H, 8-H), 3.27 (t, *J* = 9.1 Hz, 1H, 7-H), 4.40 (br, 1H, NH), 7.14 (d, *J* = 8.0 Hz, 2H, aromatic H), 7.19–7.36 (m, 10H, aromatic H), 7.41 (d, *J* = 8.0 Hz, 2H, aromatic H); ^13^C NMR (CDCl_3_) δ = 21.44 (CH_3_), 25.01 (CH_2_), 26.81 (2CH_2_), 32.01 (C-4), 35.23 (C-6), 35.35 (C-9), 38.65 (C-8), 40.56 (C-2’), 41.08 (C-7), 46.22 (N(CH_2_)_2_), 47.79 (C-3), 55.54 (C-1’), 57.94 (C-5), 68.81 (C-1), 126.25, 126.55, 126.88, 127.30, 128.25, 128.40, 128.69, 128.35 (aromatic C), 137.13, 142.86, 144.21, 145.61 (aromatic C_q_); HRMS (EI+) calcd for C_34_H_43_N_3_O_2_S: 557.3076; found: 557.3071.

##### *rac*-(7R,8R)-4-Methyl-N-{2-[7,8-diphenyl-5-(pyrrolidin-1-yl)-2-azabicyclo[3.2.2]no nan-2-yl]ethyl}benzenesulfonamide (**20**)

The reaction of 0.390 g 2-azabicyclo-nonane **8** (1.00 mmol), 0.366 g toluene-4-sulfonyl chloride (1.92 mmol) and 0.243 g 4-DMAP (1.99 mmol) in 15 mL CH_2_Cl_2_ abs. gave a crude product, which was purified by multiple column chromatography (first time on neutral aluminum oxide, CH/EtAc = 1 + 1 and in addition 1% diethylamine; second time on neutral aluminum oxide, CH/EtAc = 3+1+1% diethylamine) yielding 0.210 g **20** (39%) as colorless oil. IR = 2931, 2871, 1626, 1599, 1494, 1450, 1340, 1331, 1161, 1093, 1031, 939, 814, 755, 701; ^1^H NMR (CDCl_3_) δ = 1.78 (br s, 4H, (CH_2_)_2_), 1.91 (br t, *J* = 6.0 Hz, 2H, 4-H), 2.02 (dd, *J* = 13.6, 8.0 Hz, 1H, 6-H), 2.16–2.29 (m, 4H, 1’-H, 6-H, 9-H), 2.37 (s, 3H, CH_3_), 2.39–2.44 (m, 1H, 1’-H), 2.45–2.54 (m, 1H, 2’-H), 2.52 (d, *J* = 3.3 Hz, 1H, 1-H), 2.54–2.61 (m, 1H, 3-H), 2.70-2.81 (m, 6H, 2’-H, 3-H, N(CH_2_)_2_), 3.22–3.29 (m, 1H, 8-H), 3.32 (br t, *J* = 9.0 Hz, 1H, 7-H), 4.26 (br, 1H, NH), 7.15–7.44 (m, 14H, aromatic H); ^13^C NMR (CDCl_3_) δ = 21.46 (CH_3_), 23.63 ((CH_2_)_2_), 32.65 (C-4), 35.84 (C-6), 36.33 (C-9), 38.06 (C-8), 40.07 (C-7), 40.59 (C-2’), 45.15 (N(CH_2_)_2_), 47.83 (C-3), 55.86 (C-1’), 56.52 (C-5), 69.22 (C-1), 126.25, 126.57, 126.91, 127.47, 128.38, 128.48, 128.71, 129.35 (aromatic C), 137.26, 142.87, 144.33, 145.70 (aromatic C_q_); HRMS (EI+) calcd for C_33_H_41_N_3_O_2_S: 543.2919; found: 543.2964.

### 4.3. Biological Tests

#### 4.3.1. In Vitro Microplate Assay against *P. Falciparum*

In vitro activity against erythrocytic stages of *P. falciparum* was determined using a ^3^H-hypoxanthine incorporation assay [19,20], using the drug-sensitive NF54 strain [21] or the chloroquine- and pyrimethamine-resistant K_1_ strain [22]. Compounds were dissolved in DMSO at 10 mg/mL and added to parasite cultures incubated in RPMI 1640 medium without hypoxanthine, supplemented with HEPES (5.94 g/L), NaHCO_3_ (2.1 g/L), neomycin (100 U/mL), Albumax (5 g/L) and washed human red cells A+ at 2.5% hematocrit (0.3% parasitemia). Serial drug dilutions of 11 three-fold dilution steps covering a range from 100 to 0.002 µg/mL were prepared. The 96-well plates were incubated in a humidified atmosphere at 37 °C; 4% CO_2_, 3% O_2_, 93% N_2_. After 48 h, 0.05 mL of ^3^H-hypoxanthine (=0.5 µCi) was added to each well of the plate. The plates were incubated for an additional 24 h under the same conditions. The plates were then harvested with a Betaplate cell harvester (Wallac, Zurich, Switzerland). The red blood cells were transferred onto a glass fiber filter and washed with distilled water. The dried filters were inserted into a plastic foil with 10 mL of scintillation fluid and counted in a Betaplate liquid scintillation counter (Wallac, Zurich, Switzerland). IC_50_ values were calculated from sigmoidal inhibition curves by linear regression [23] using Microsoft Excel. Chloroquine (Sigma C6628) was used as control. 

#### 4.3.2. In Vitro Microplate Assay against *T. Brucei Rhodesiense*

Minimum essential medium (50 µL) supplemented with 25 mM HEPES, 1 g/L additional glucose, 1% MEM nonessential amino acids (100×), 0.2 mM 2-mercaptoethanol, 1 mM Na-pyruvate and 15% heat inactivated horse serum was added to each well of a 96-well microtiter plate. Serial drug dilutions of 11 three-fold dilution steps covering a range from 100 to 0.002 μg/mL were prepared. Then, 4 × 10^3^ bloodstream forms of *T. b. rhodesiense* STIB 900 in 50 µL was added to each well and the plate incubated at 37 °C under a 5% CO_2_ atmosphere for 70 h. Ten microliters of resazurin solution (resazurin, 12.5 mg in 100 mL double-distilled water) was then added to each well and incubation continued for an additional 2–4 h [24]. The plates were then read with a Spectramax Gemini XS microplate fluorometer (Molecular Devices Cooperation, Sunnyvale, CA, USA) using an excitation wavelength of 536 nm and an emission wavelength of 588 nm. Data were analyzed with the graphic program Softmax Pro (Molecular Devices Cooperation, Sunnyvale, CA, USA), which calculated IC_50_ values by linear regression [23] and 4-parameter logistic regression from the sigmoidal dose inhibition curves. Melarsoprol (Arsobal Sanofi-Aventis, received from WHO) was used as control.

#### 4.3.3. In Vitro Cytotoxicity with L-6 Cells

Assays were performed in 96-well microtiter plates, each well containing 0.1 mL of RPMI 1640 medium supplemented with 1% L-glutamine (200 mM) and 10% fetal bovine serum and 4000 L-6 cells (a primary cell line derived from rat skeletal myoblasts, ATCC CRL-1458™) [25,26]. Serial drug dilutions of 11 three-fold dilution steps covering a range from 100 to 0.002 μg/mL were prepared. After 70 h of incubation, the plates were inspected under an inverted microscope to assure the growth of the controls and sterile conditions. Then, 0.01 mL resazurin solution (resazurin, 12.5 mg in 100 ml double-distilled water) was added to each well and the plates incubated for another 2 h. The plates were read with a Spectramax Gemini XS microplate fluorometer (Molecular Devices Cooperation, Sunnyvale, CA, USA) using an excitation wavelength of 536 nm and an emission wavelength of 588 nm. The IC_50_ values were calculated by linear regression [23] from the sigmoidal dose inhibition curves using SoftmaxPro software (Molecular Devices Cooperation, Sunnyvale, CA, USA). Podophyllotoxin (Sigma P4405) was used as control.

## Figures and Tables

**Table 1:** Activities of compounds **6**–**20** against *P. falciparum* NF54, *P. falciparum* K_1_, *T. brucei rhodesiense* and L-6 cells,. expressed as IC_50_ (µM) ^a^.

Compd.	*P. Falciparum* NF54 ^b^	S.I. = IC_50_ (Cyt.)/IC_50_ (*P.f.* NF54)	*P. Falciparum* K_1_^c^	S.I. = IC_50_ (Cyt.)/IC_50_ (*P.f.* K_1_)	*T. Brucei* *Rhodesiense*	S.I. = IC_50_ (Cyt.)/IC_50_ (*T.b.r.*)	Cytotox. L-6 Cells
**6**			0.360	59.7	2.92	7.36	21.5
**7**			0.180	93.3	1.94	8.66	16.8
**8**			0.540	47.8	1.13	22.8	25.8
**9**			0.630	14.5	0.420	21.7	9.12
**11A**	2.98	6.75			6.61	3.04	20.1
**11B**	1.32	4.99			4.92	1.34	6.59
**12A**	0.252	11.2			2.20	1.29	2.83
**12B**	1.33	1.92			1.52	1.68	2.56
**13A**	0.446	5.72			0.329	7.75	2.55
**13B**	0.680	3.03			1.00	2.06	2.06
**14**	1.94	8.76			1.20	14.17	17.0
**15**	0.742	5.62			1.30	3.21	4.17
**16 ^d^**			1.26	8.33	2.65	3.96	10.5
**17 ^d^**			1.30	7.25	1.26	7.48	9.42
**18**	0.606	10.4			1.19	5.28	6.29
**19**	0.577	18.7			1.15	9.39	10.8
**20**	0.487	14.7			0.647	11.0	7.15
POD							0.012
CQ	0.0039	23308	0.15	606			90.9
MEL					0.009	864	7.78

POD = podophyllotoxin; CQ = chloroquine; MEL = melarsoprol; ^a^ Values represent the average of four determinations (two determinations of two independent experiments); ^b^ sensitive to chloroquine and pyrimethamine; ^c^ resistant to chloroquine and pyrimethamine; ^d^ reference [15].

## Data Availability

The data presented in this study are available in this article.

## Supplementary Materials

The following are available online at https://www.mdpi.com/article/10.3390/molecules27196217/s1, Figures S1–S16: ^1^H-, ^13^C-NMR and MS-spectra. Tables S1–S6: Crystal data.

## Sample Availability

Samples of selected compounds are available from the authors.
