# Supplementary material for: Antiprotozoal Activity of Azabicyclo-Nonanes Linked to Tetrazole or Sulfonamide Cores
Source: Molecules. 2022 Sep 21;27(19):6217. doi: 10.3390/molecules27196217 (PMC9572860; doi:10.3390/molecules27196217)
Supplement: Supplementary file 1 [file molecules-27-06217-s001.zip › molecules-1895565-supplementary.pdf]

Supplementary Information

Antiprotozoal Activity of Azabicyclononanes Linked to Tetrazole or Sulfonamide Cores

Johanna Dolensky [1], Clemens Hinteregger [1], Andreas Leitner [1], Werner Seebacher [1], Robert Saf [2], Ferdinand Belaj [3], Pascal Mäser [4], Marcel Kaiser [4] and Robert Weis [1,*]

[1] Pharmaceutical Chemistry, Institute of Pharmaceutical Sciences, University of Graz, Schubertstraße 1, A-8010 Graz, Austria
[2] Institute for Chemistry and Technology of Materials (ICTM), Graz University of Technology, Stremayrgasse 9, A-8010 Graz, Austria
[3] Inorganic Chemistry, Institute of Chemistry, University of Graz, Schubertstraße 1, A-8010 Graz, Austria
[4] Swiss Tropical and Public Health Institute, Socinstraße 57, CH-4002 Basel, Switzerland
* Correspondence: robert.weis@uni-graz.at; Tel.: +43-316-380-5379

Abstract: *N*-(Aminoalkyl)**a**zabicyclo[3.2.2]nonanes possess antiplasmodial and antitrypanosomal activity. A series with terminal tetrazole or sulfonamido partial structure was prepared. The structures of all new compounds were confirmed by NMR and IR spectroscopy as well as by mass spectral data. A single crystal structure analysis enabled the distinction between isomers. The antitrypanosomal activities were examined in vitro against strains of *Plasmodium falciparum* and *Trypanosoma brucei rhodesiense* (STIB 900). The most active sulfonamide and tetrazole derivatives showed activities in the submicromolar range.

---

1. NMR and MS spectra of compounds **6–15** and **18–20**.........Figures S1–S16

2. Crystal data of **12B**..Tables S1–S6

Figure S1. ^{1}H NMR at 400 MHz, ^{13}C NMR at 100 MHz spectra, and MS spectra for compound **6**

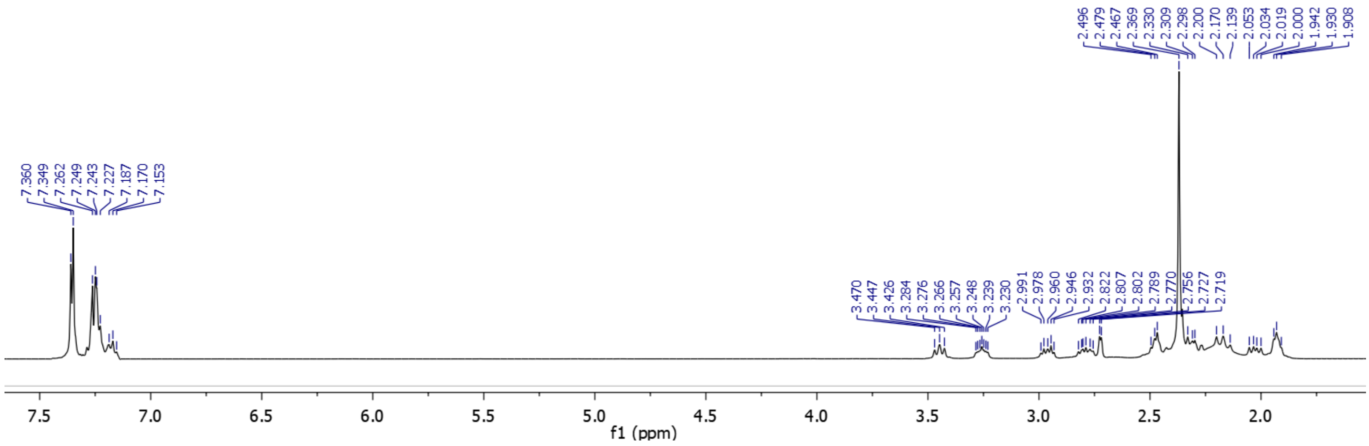

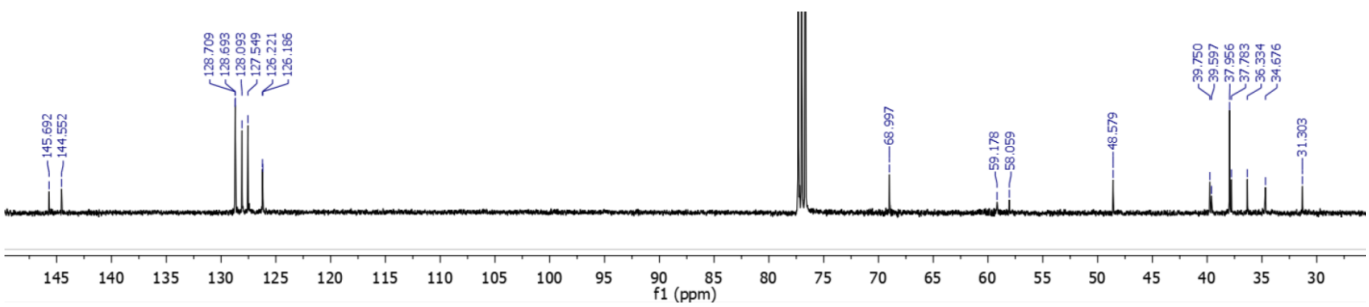

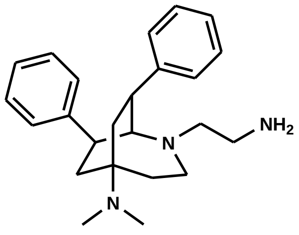

6

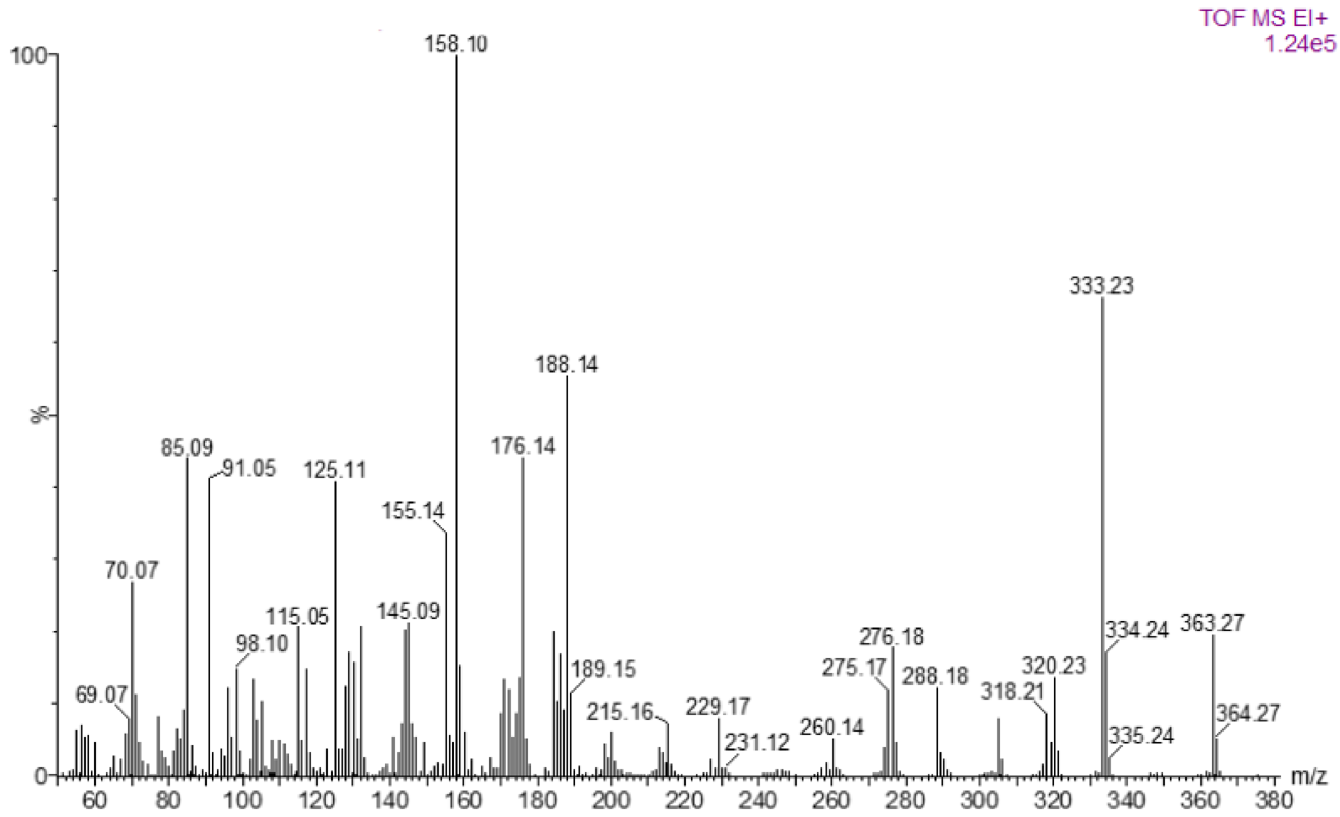
TOF MS EI+
1.24e5
158.10
333.23
188.14
176.14
85.09
91.05
125.11
155.14
70.07
145.09
115.05
98.10
189.15
69.07
215.16
229.17
231.12
260.14
276.18
275.17
288.18
320.23
318.21
334.24
363.27
364.27
335.24
m/z

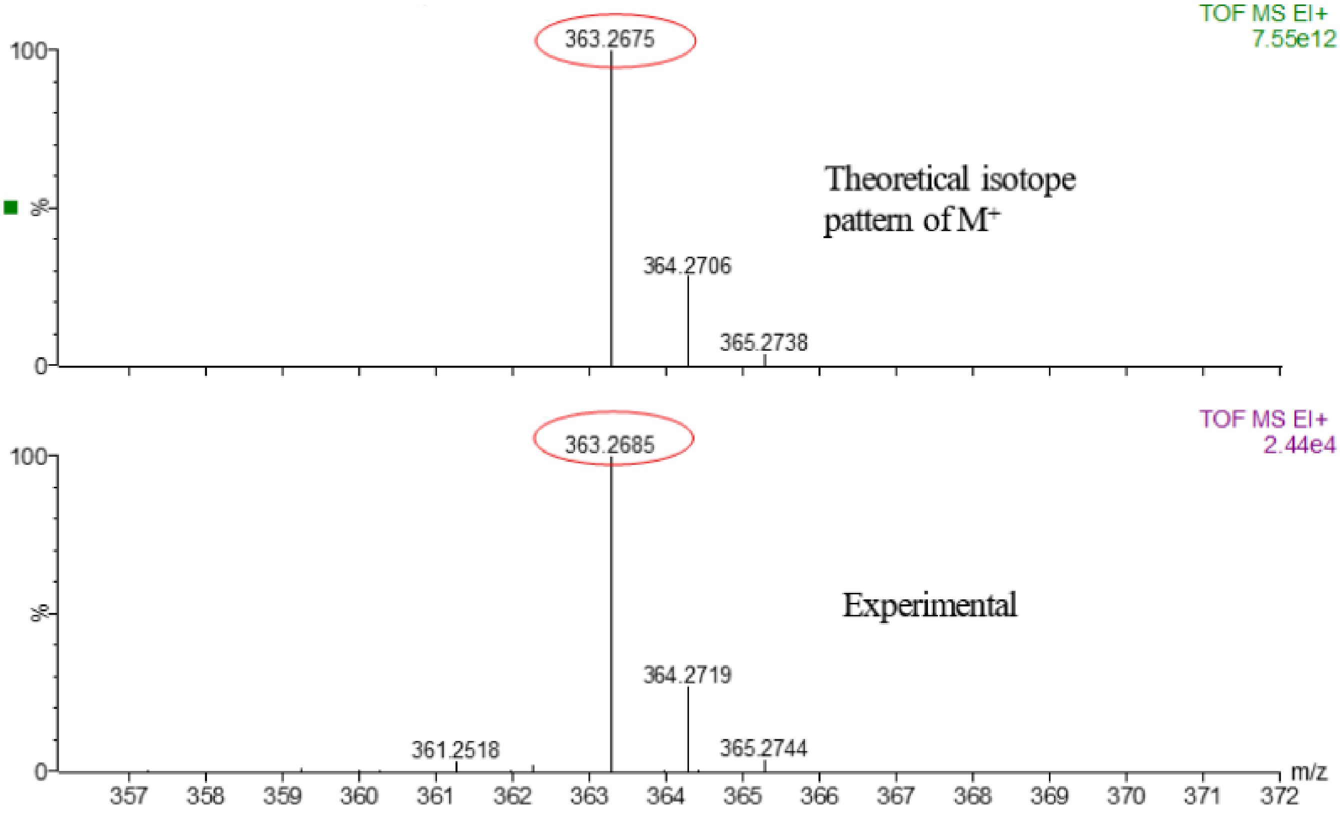
TOF MS EI+
7.55e12
363.2675
Theoretical isotope pattern of M+
364.2706
365.2738
TOF MS EI+
2.44e4
363.2685
Experimental
364.2719
361.2518
365.2744
m/z

Figure S2. ^{1}H NMR at 400 MHz, ^{13}C NMR at 100 MHz spectra, and MS spectra for compound **7**

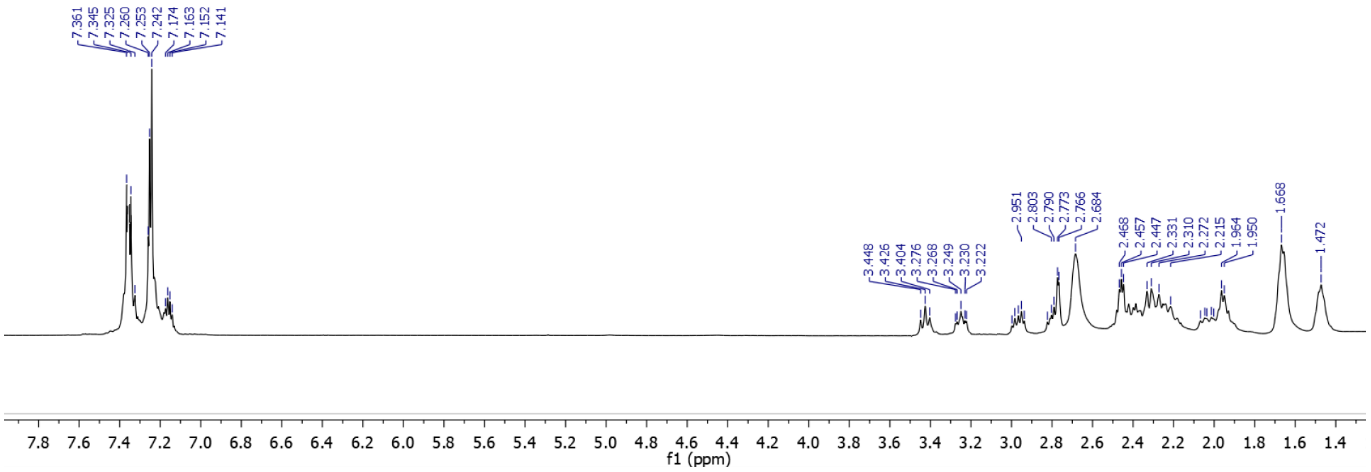

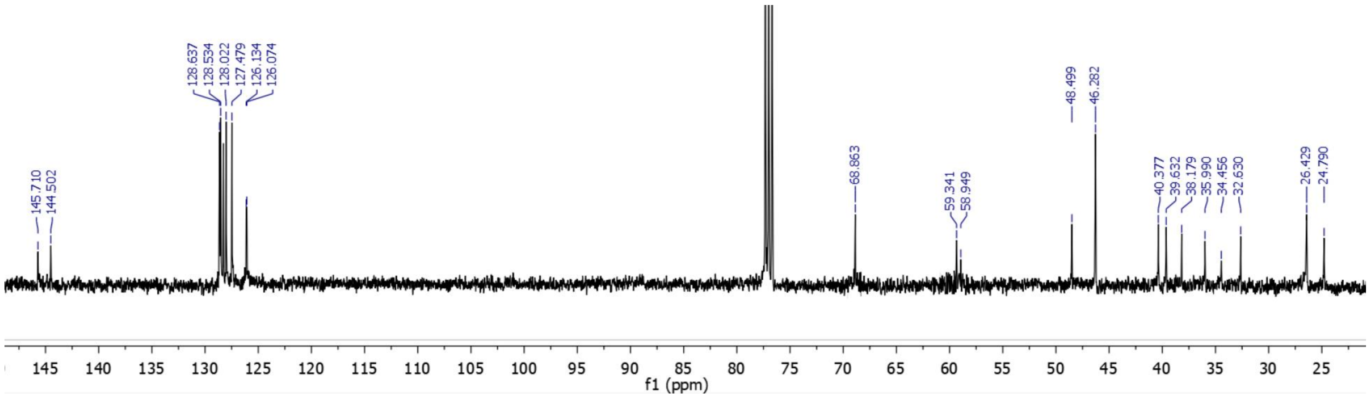

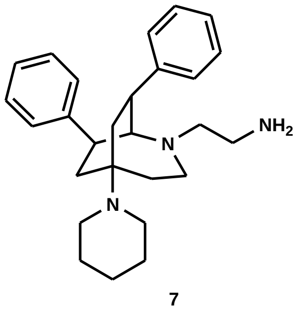

7

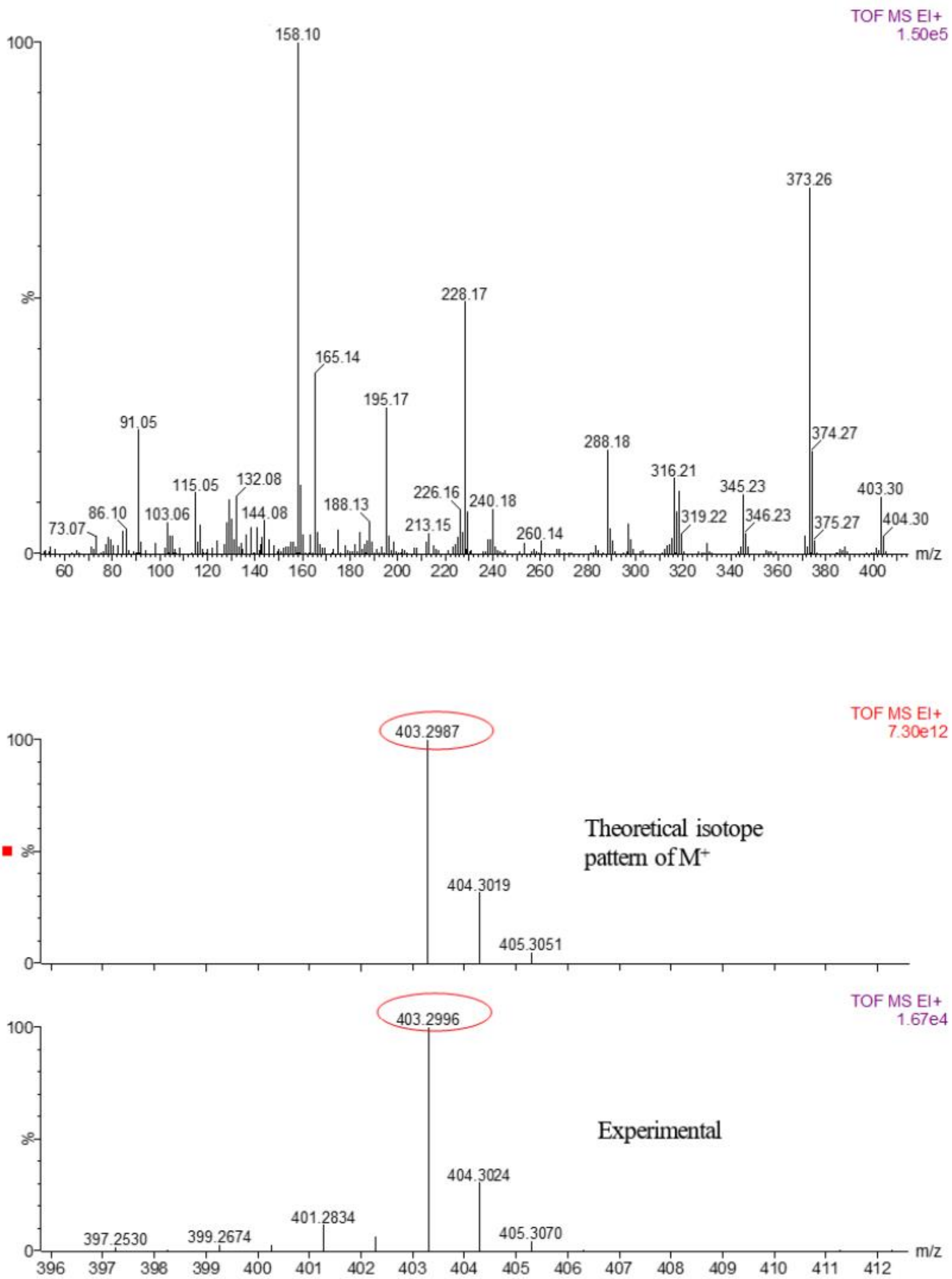
TOF MS EI+
1.50e5
100
%
0
158.10
373.26
228.17
165.14
195.17
91.05
374.27
288.18
316.21
115.05
132.08
345.23
403.30
226.16
240.18
188.13
86.10
103.06
144.08
319.22
346.23
213.15
73.07
375.27
404.30
260.14
60 80 100 120 140 160 180 200 220 240 260 280 300 320 340 360 380 400
m/z
TOF MS EI+
7.30e12
403.2987
Theoretical isotope pattern of M+
404.3019
405.3051
TOF MS EI+
1.67e4
403.2996
Experimental
404.3024
401.2834
397.2530
399.2674
405.3070
396 397 398 399 400 401 402 403 404 405 406 407 408 409 410 411 412
m/z

Figure S3. ^{1}H NMR at 400 MHz, ^{13}C NMR at 100 MHz spectra, and MS spectra for compound **8**

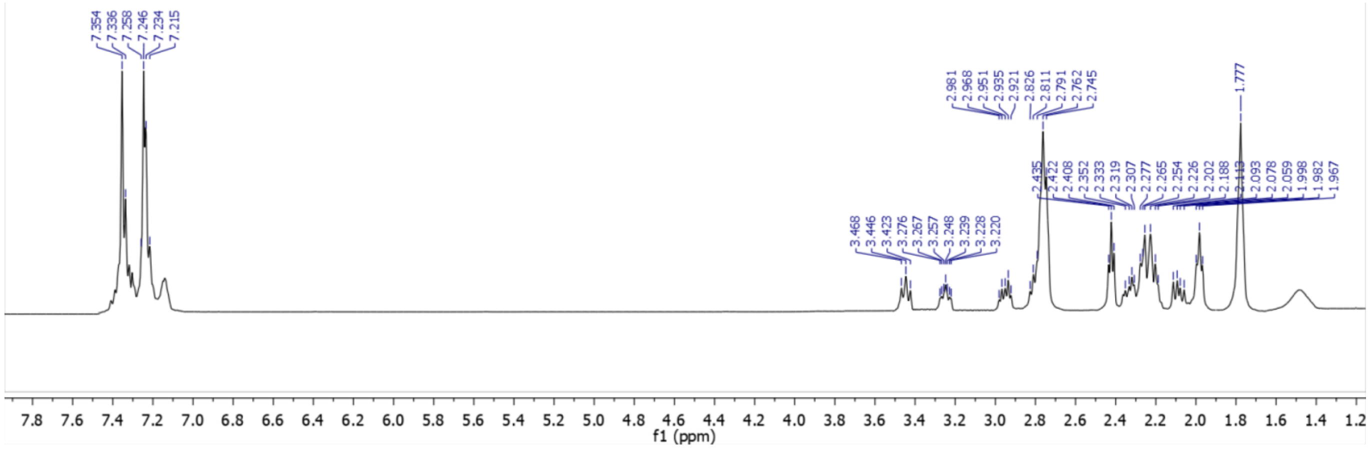

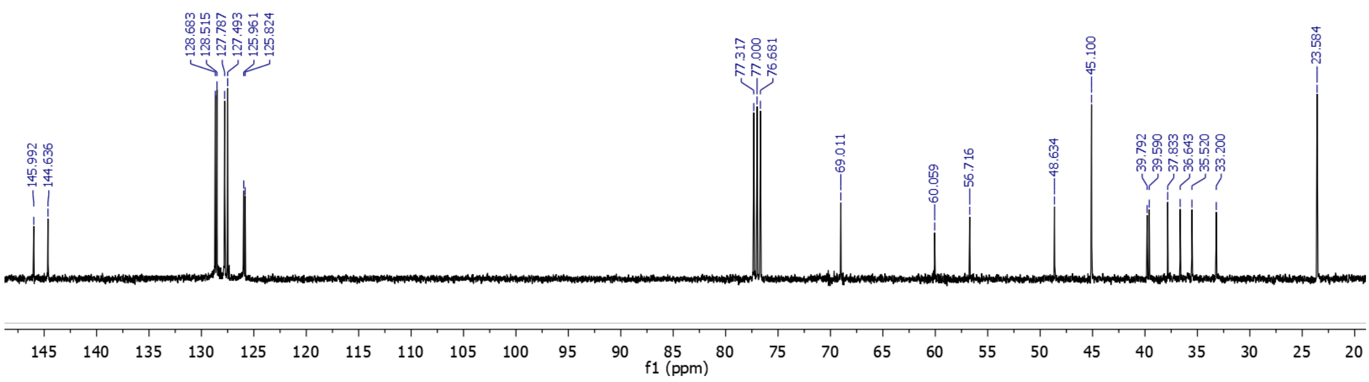

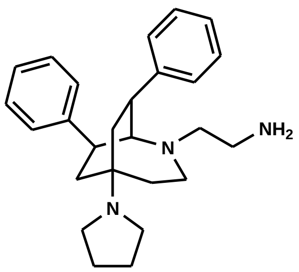

8

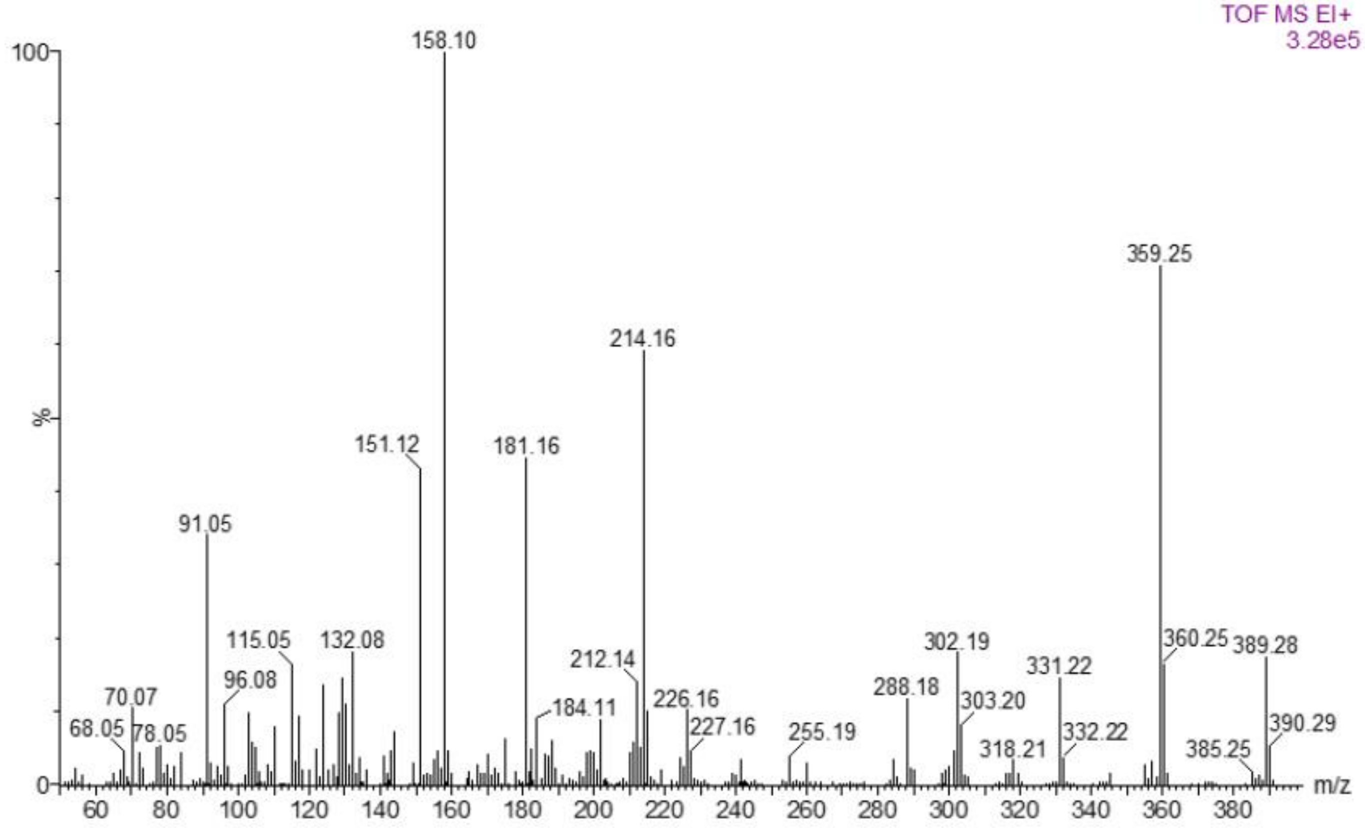
TOF MS EI+
3.28e5
158.10
359.25
214.16
151.12
181.16
91.05
115.05
132.08
302.19
360.25
389.28
212.14
331.22
70.07
96.08
288.18
226.16
303.20
184.11
68.05
78.05
227.16
255.19
318.21
332.22
390.29
385.25
100
%
0
m/z

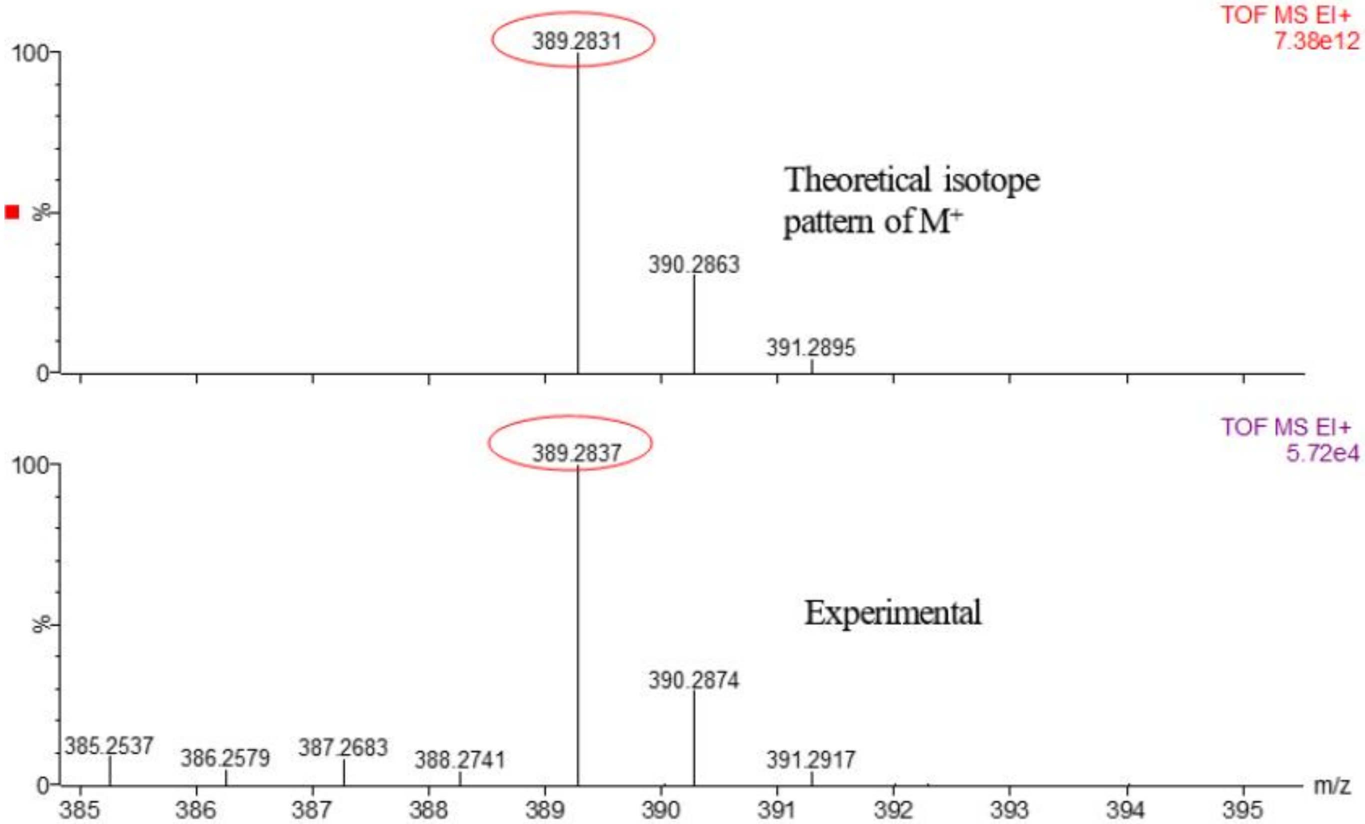
TOF MS EI+
7.38e12
389.2831
Theoretical isotope pattern of M+
390.2863
391.2895
TOF MS EI+
5.72e4
389.2837
Experimental
390.2874
385.2537
386.2579
387.2683
388.2741
391.2917
100
%
0
m/z

Figure S4. ^{1}H NMR at 400 MHz, ^{13}C NMR at 100 MHz spectra, and MS spectra for compound **9**

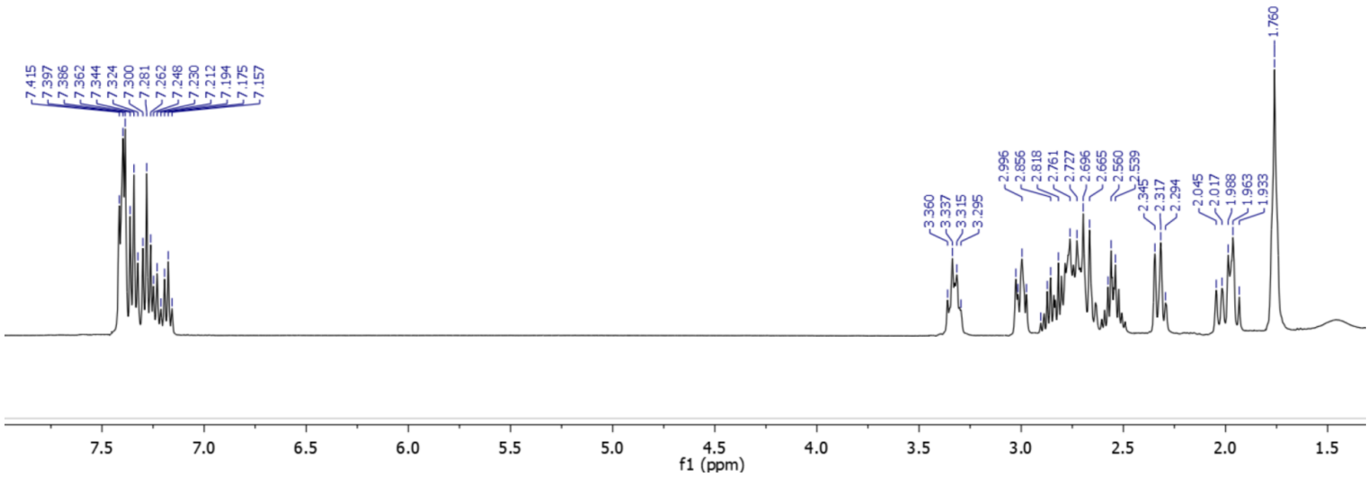

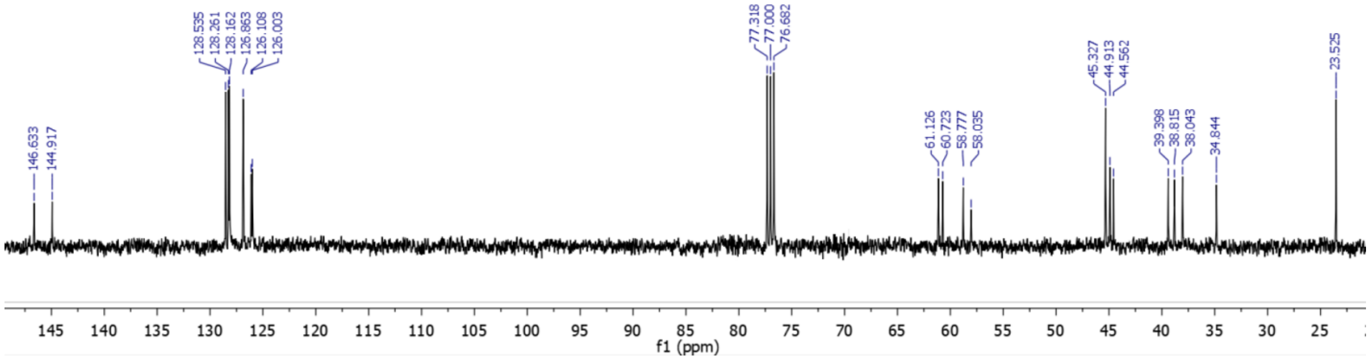

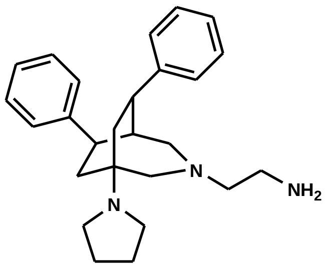

9

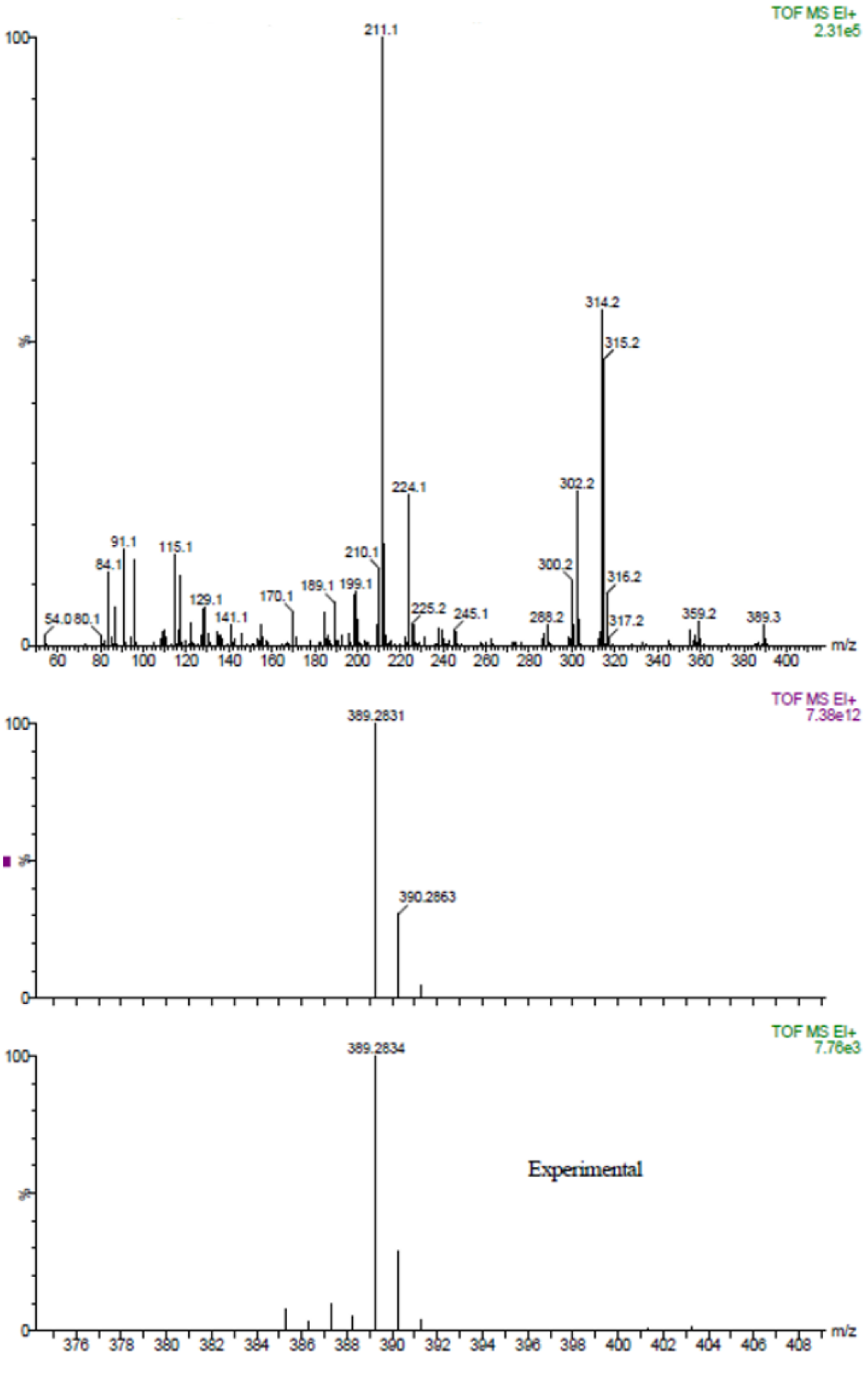
TOF MS EI+
2.31e5
211.1
314.2
315.2
224.1
302.2
91.1
115.1
84.1
210.1
300.2
316.2
189.1
199.1
129.1
170.1
225.2
245.1
141.1
54.0
80.1
288.2
317.2
359.2
389.3
m/z
TOF MS EI+
7.38e12
389.2831
390.2863
TOF MS EI+
7.76e3
389.2834
Experimental
m/z

Figure S5. ^{1}H NMR at 400 MHz, ^{13}C NMR at 100 MHz spectra, and MS spectra for compound **10**

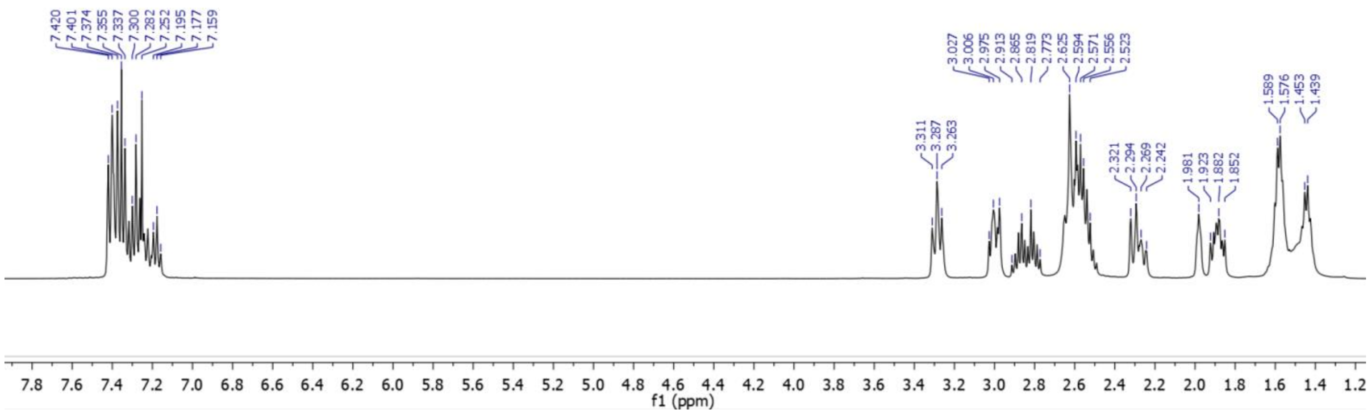

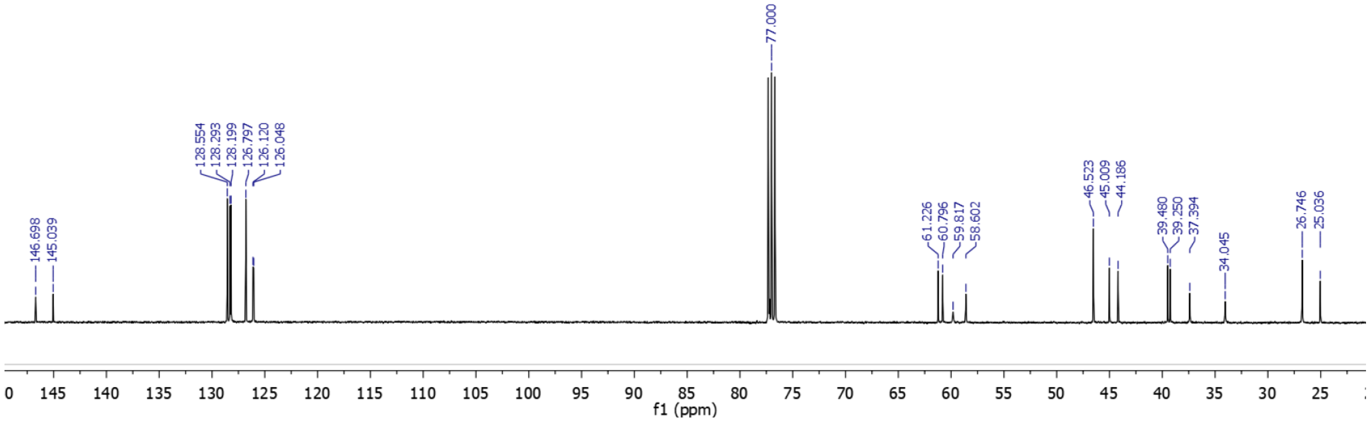

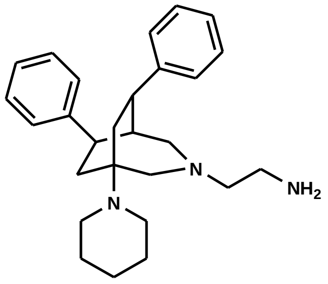

10

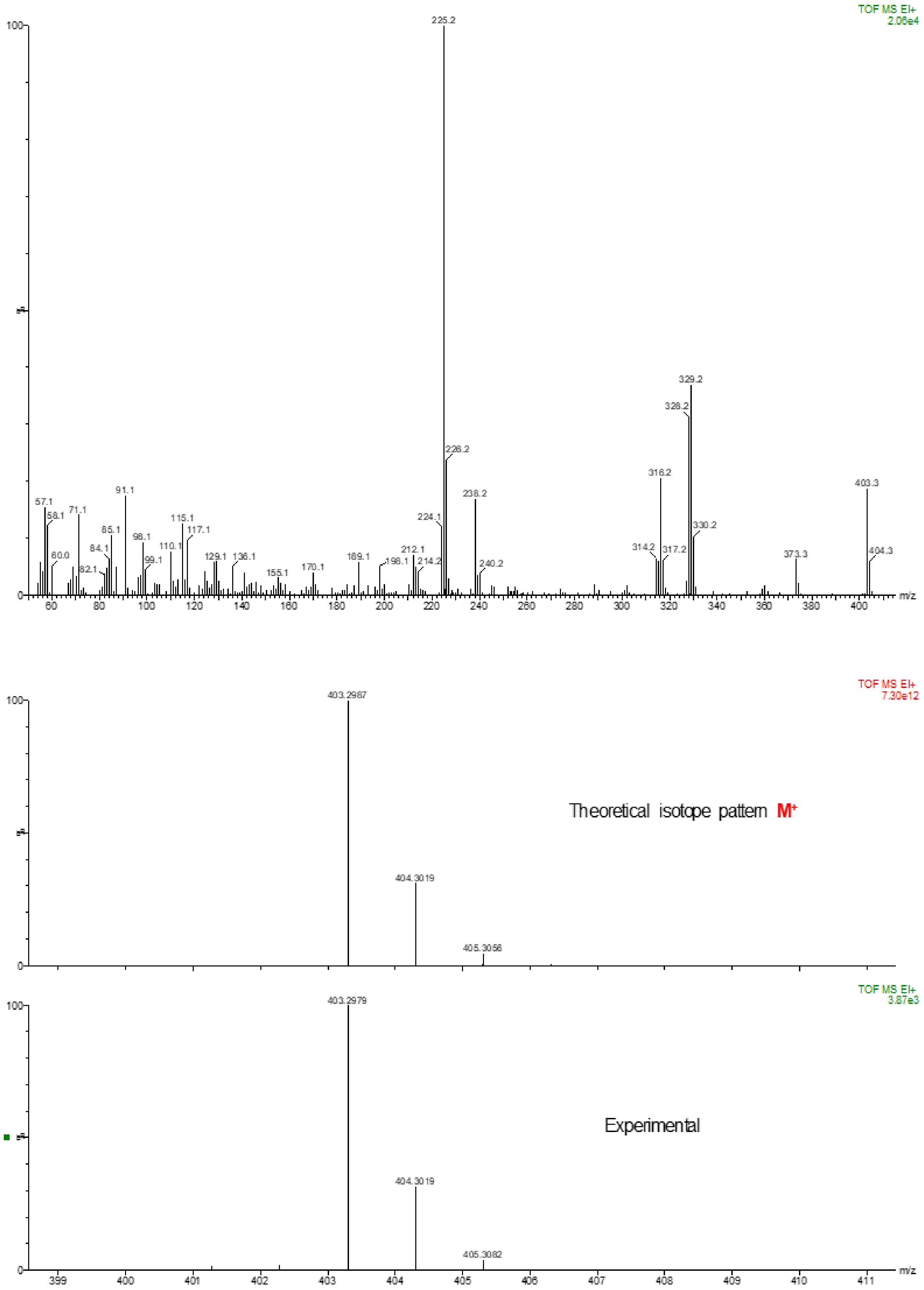
TOF MS EI+
2.06e4
225.2
329.2
328.2
226.2
316.2
403.3
238.2
57.1
91.1
71.1
58.1
115.1
117.1
85.1
98.1
84.1
110.1
99.1
60.0
82.1
129.1
136.1
155.1
170.1
189.1
198.1
212.1
214.2
224.1
240.2
314.2
317.2
330.2
373.3
404.3
m/z
TOF MS EI+
7.30e12
403.2987
404.3019
405.3056
Theoretical isotope pattern M+
TOF MS EI+
3.87e3
403.2979
404.3019
405.3082
Experimental
m/z

Figure S6. ^{1}H NMR at 400 MHz, ^{13}C NMR at 100 MHz spectra, and MS spectra for compound **11A**

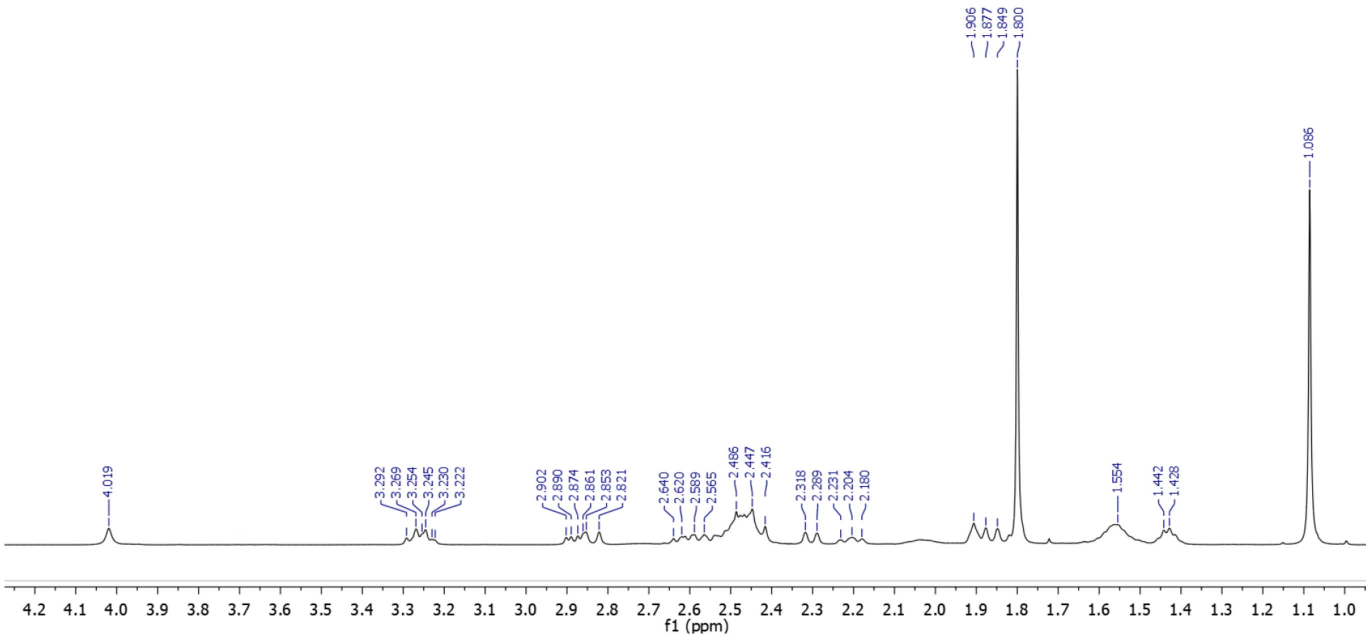

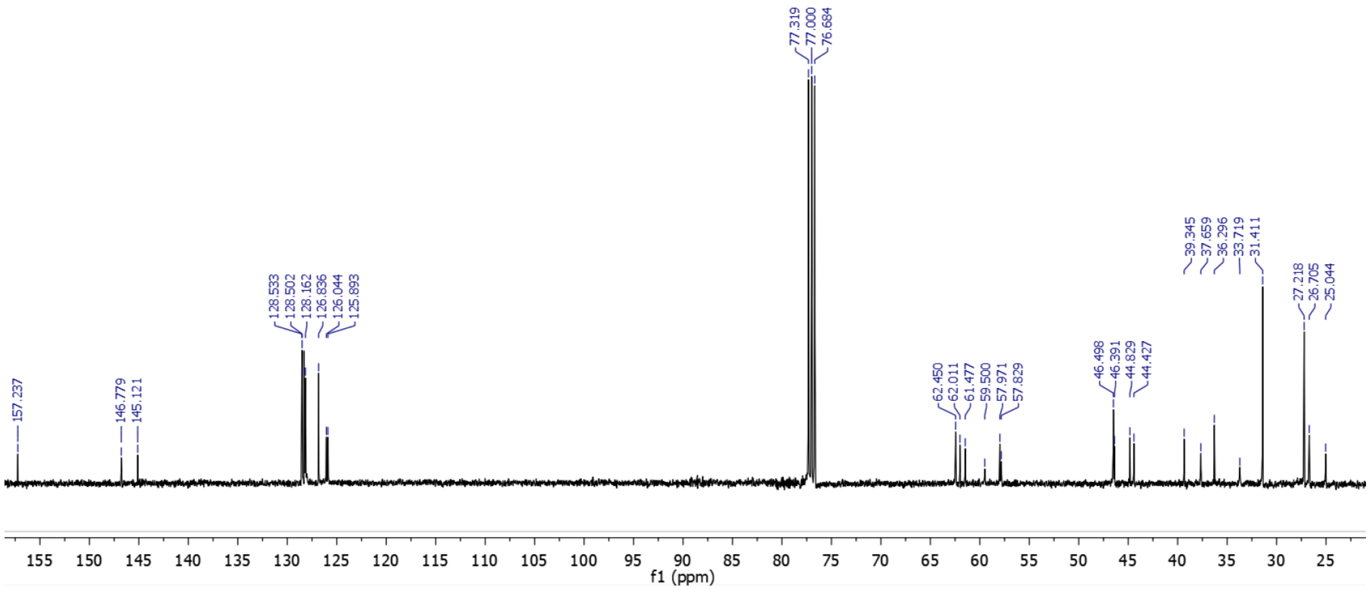

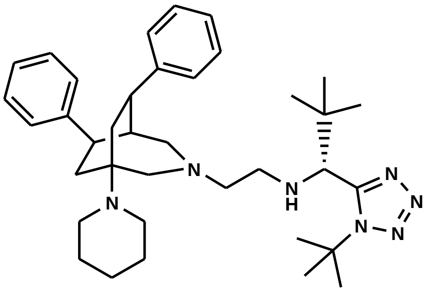

11A

Ionisation: **DI-EI**

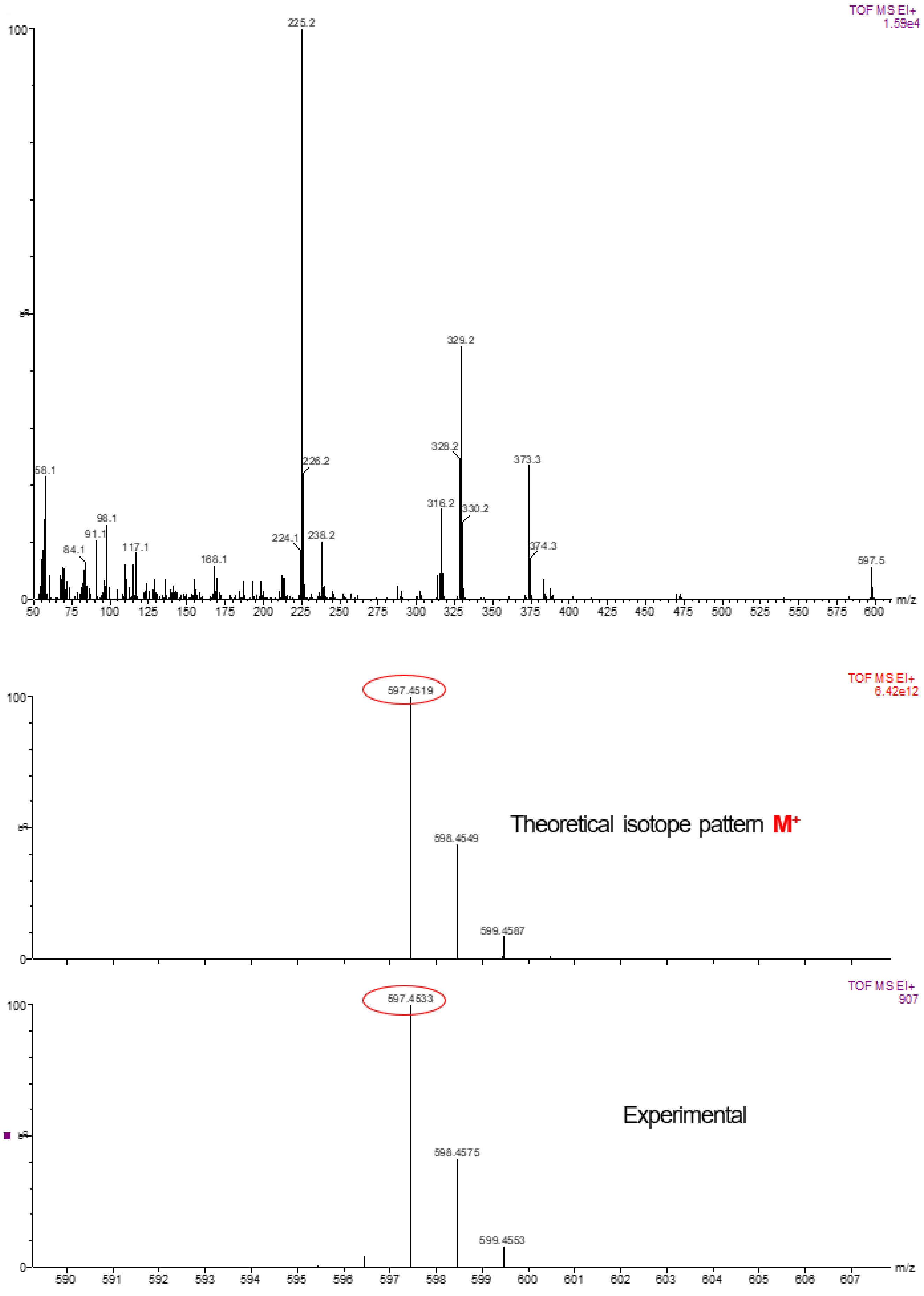

Figure S7. ^{1}H NMR at 400 MHz, ^{13}C NMR at 100 MHz spectra, and MS spectra for compound **11B**

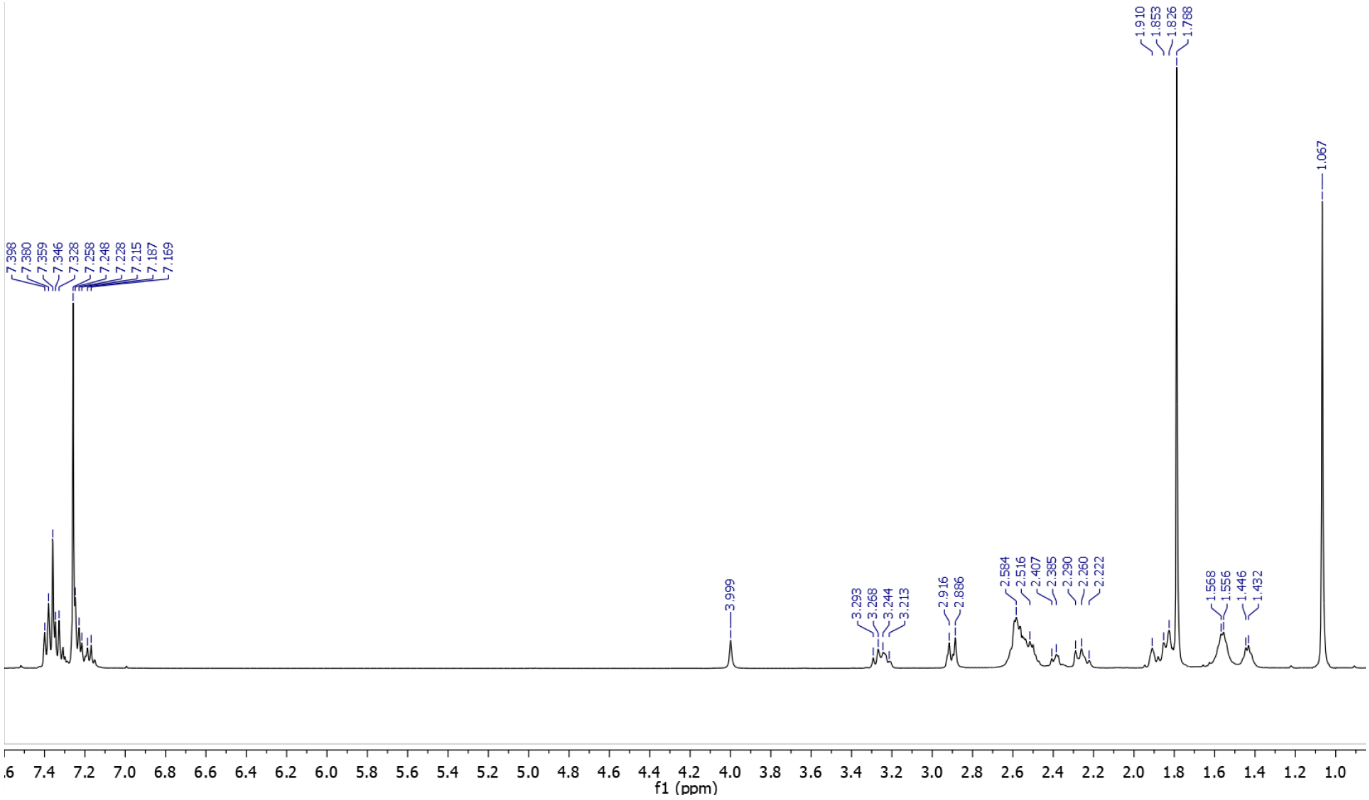

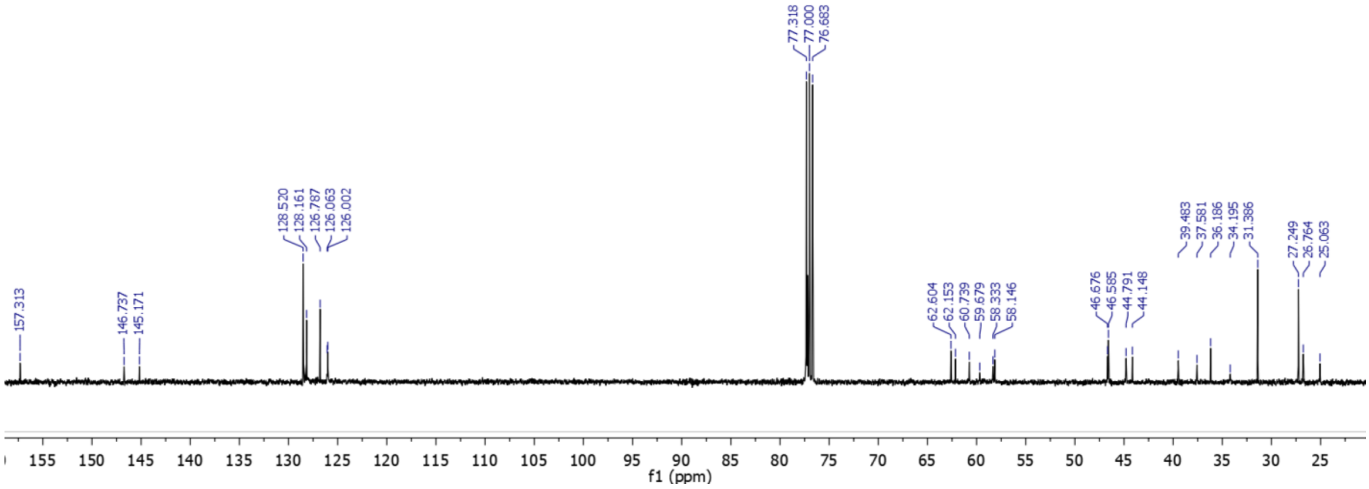

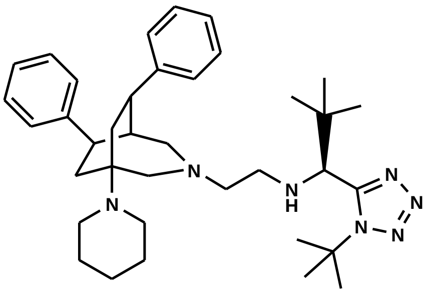

11B

Ionisation: **DI-EI**

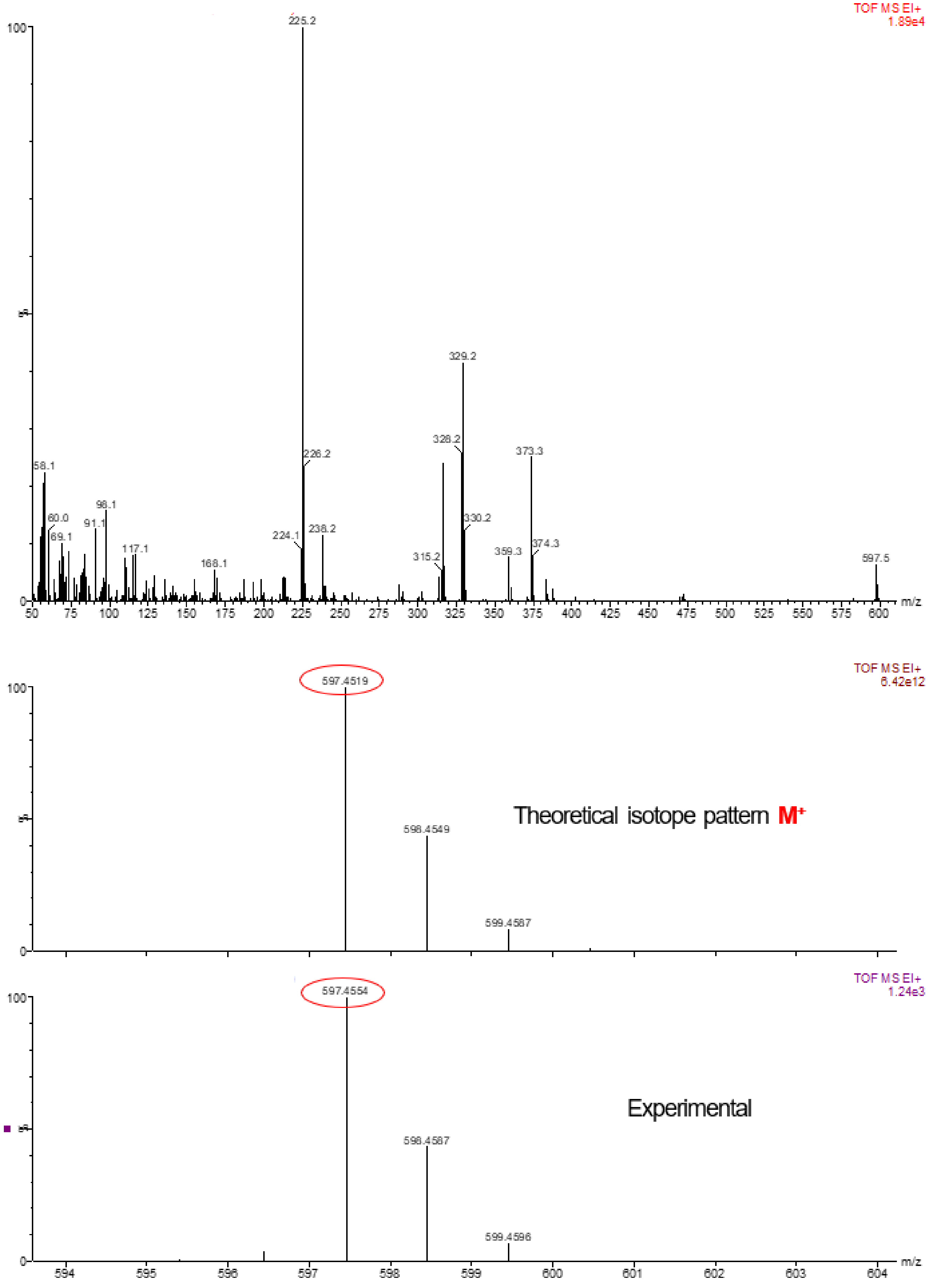

Figure S8. ^{1}H NMR at 400 MHz, ^{13}C NMR at 100 MHz spectra, and MS spectra for compound **12A**

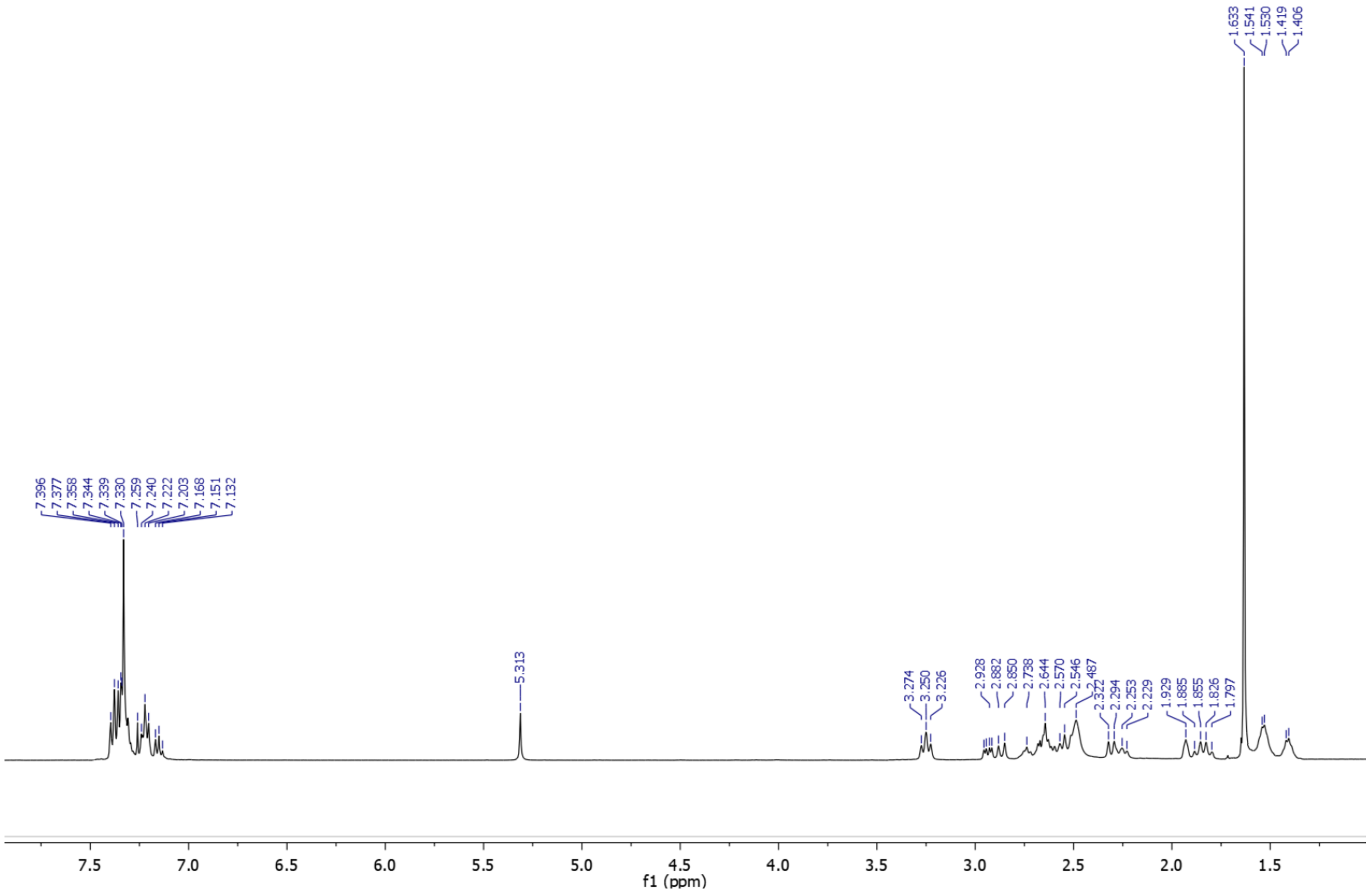

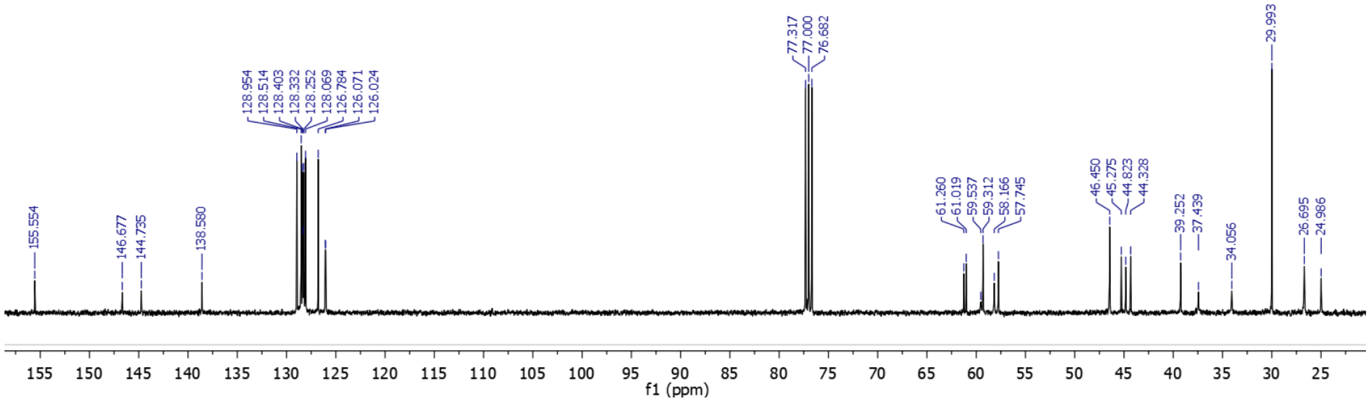

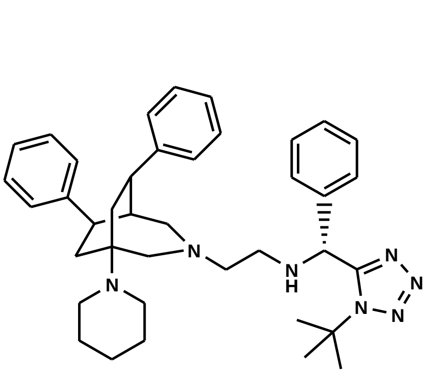

12A

Ionisation: **DI-EI**

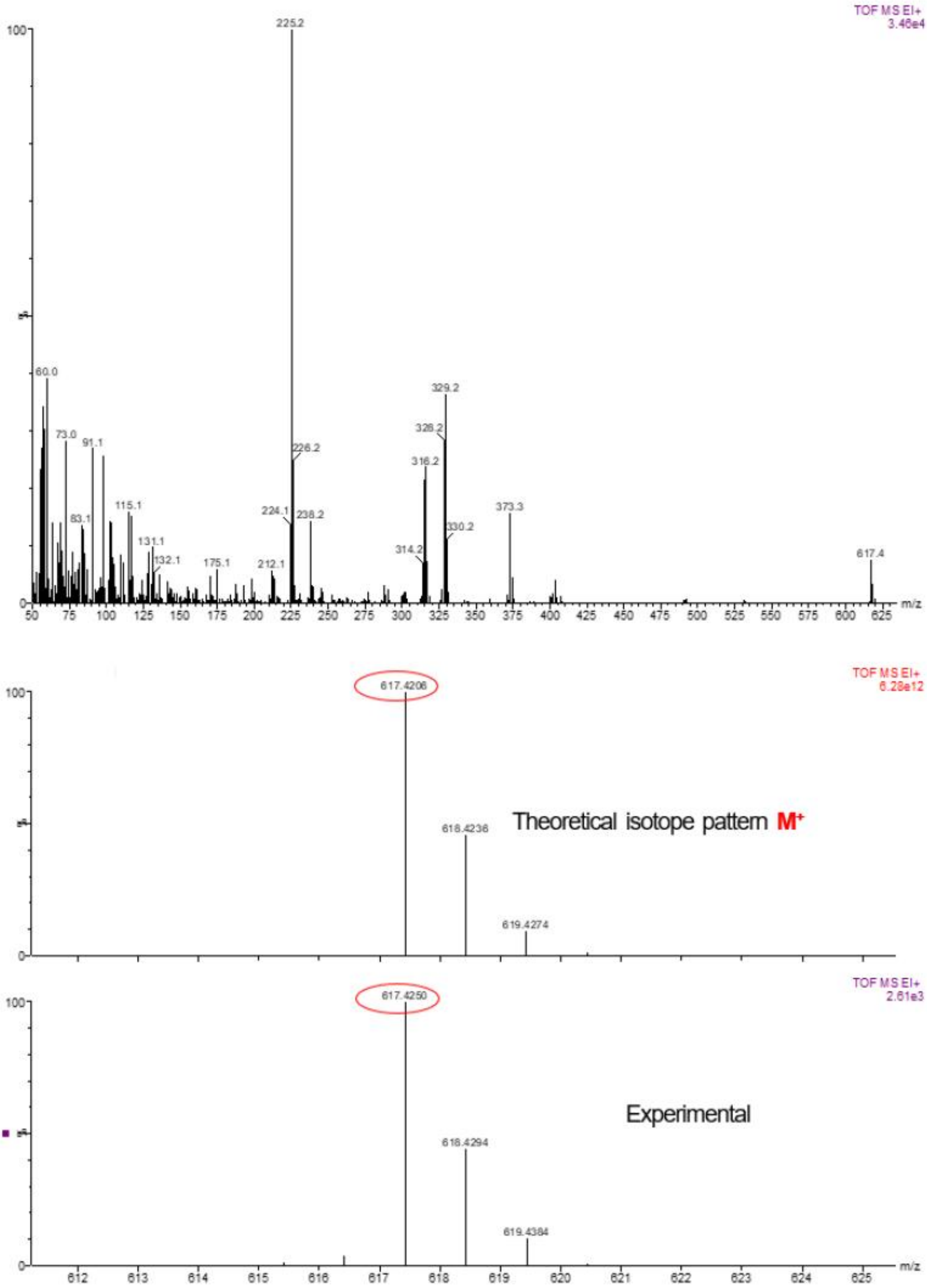

Figure S9. ^{1}H NMR at 400 MHz, ^{13}C NMR at 100 MHz spectra, and MS spectra for compound **12B**

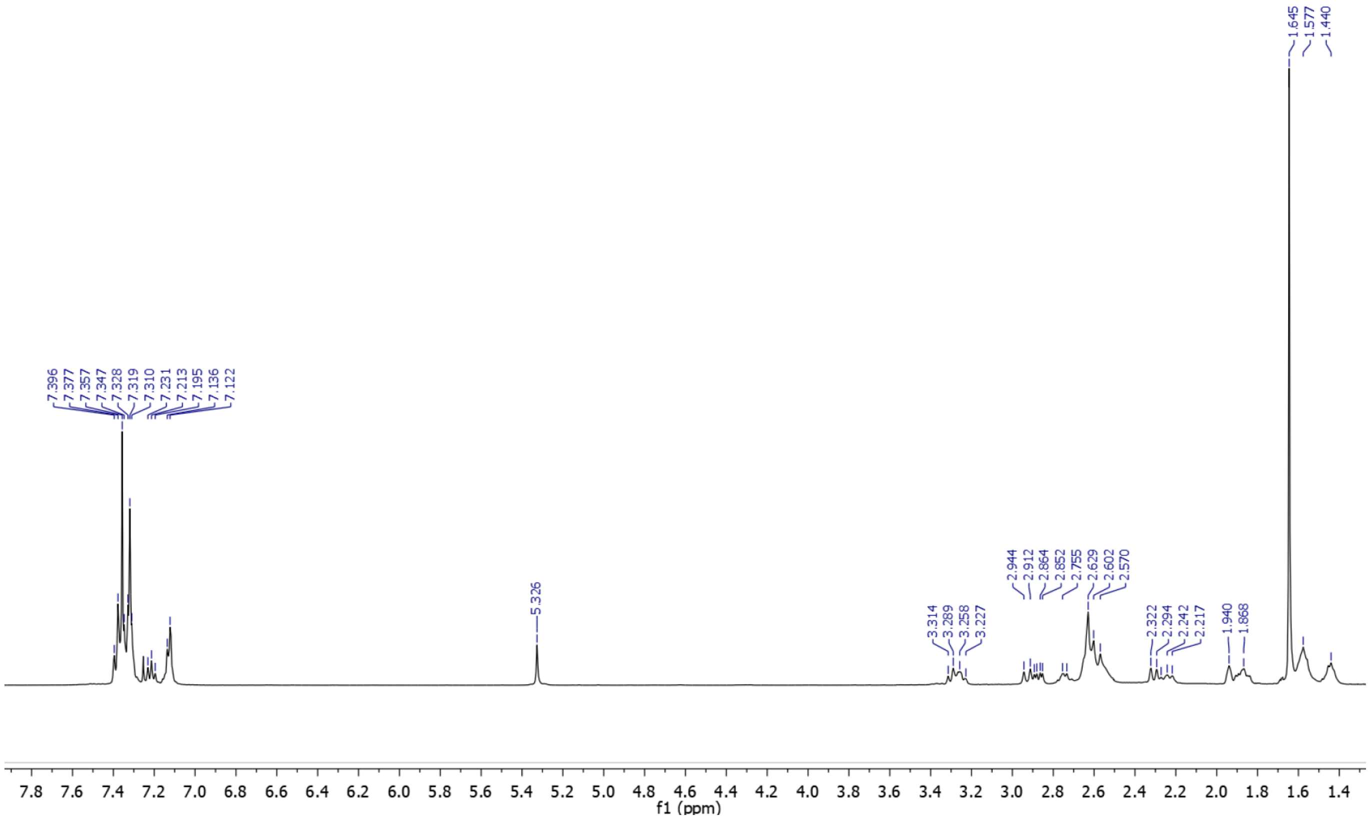

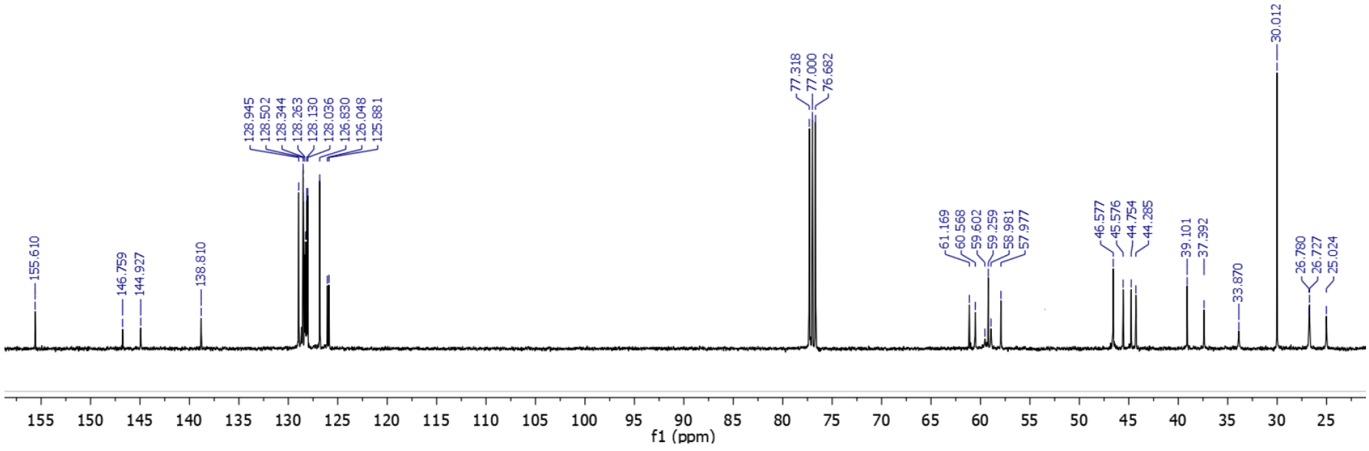

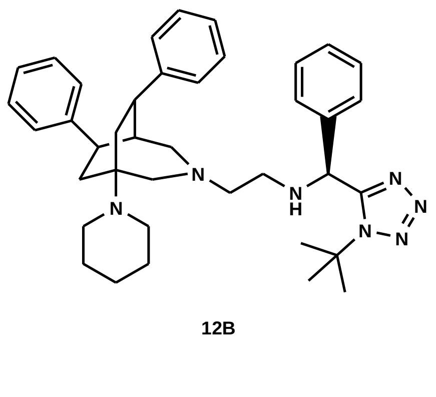

12B

Ionisation: **DI-EI**

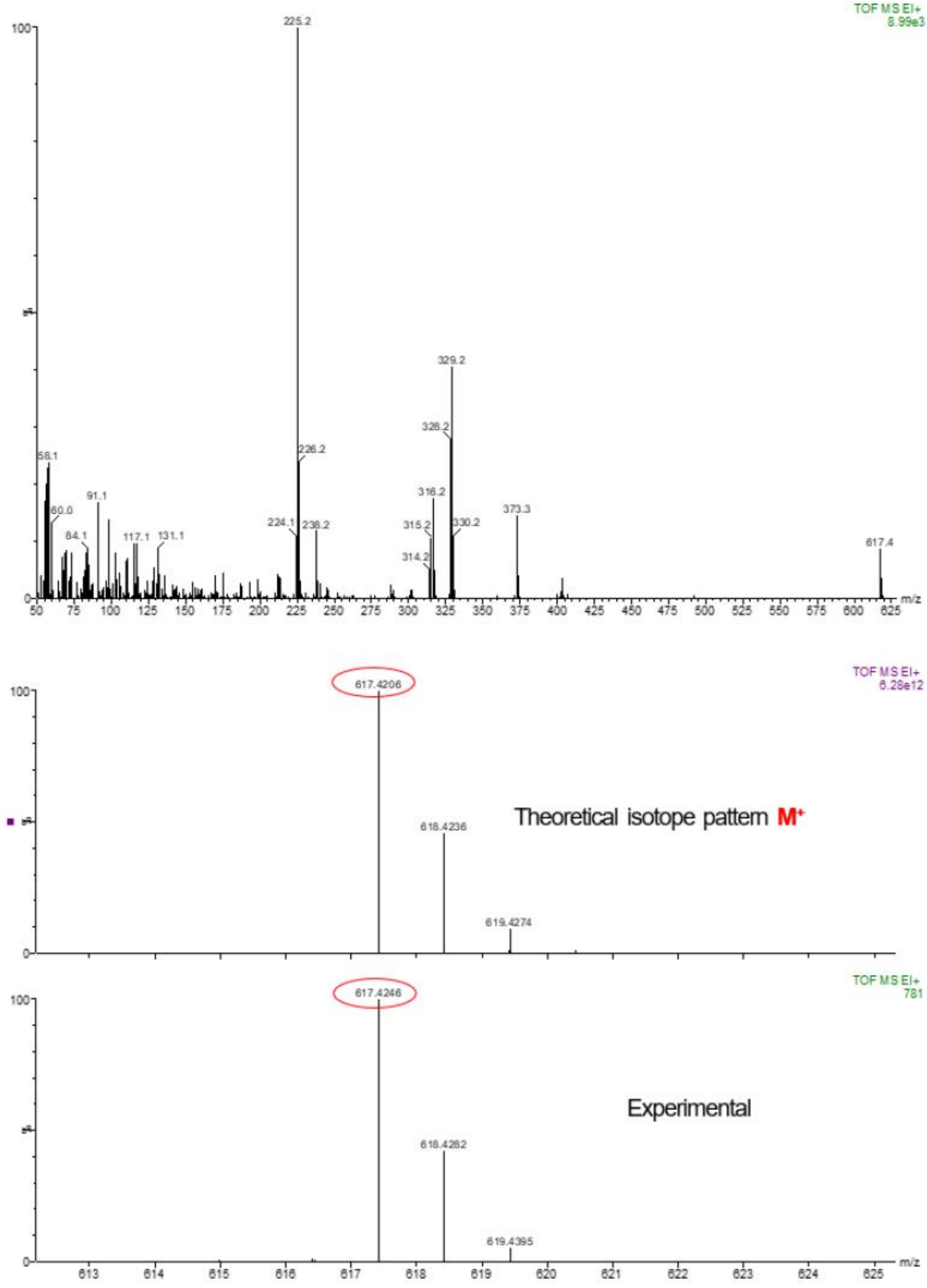

Figure S10. ^{1}H NMR at 400 MHz, ^{13}C NMR at 100 MHz spectra, and MS spectra for compound **13A**

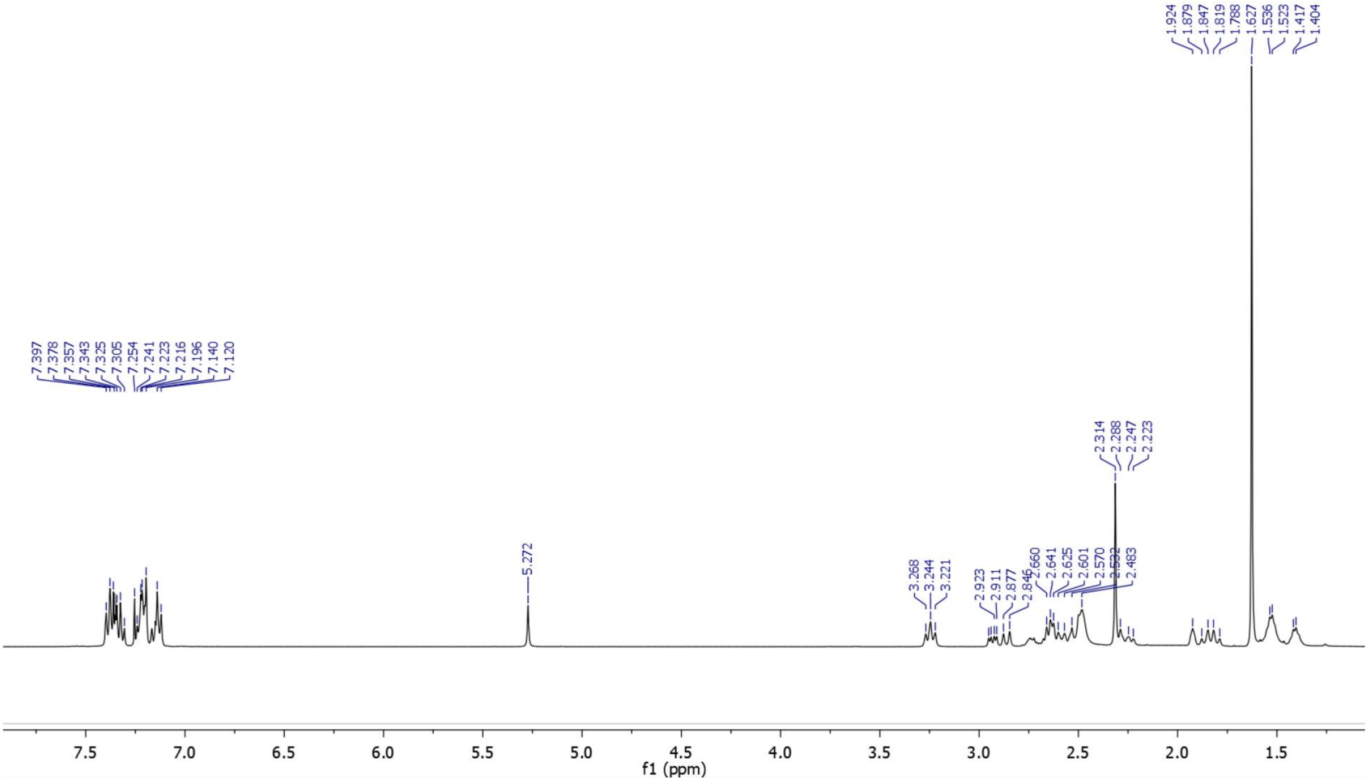

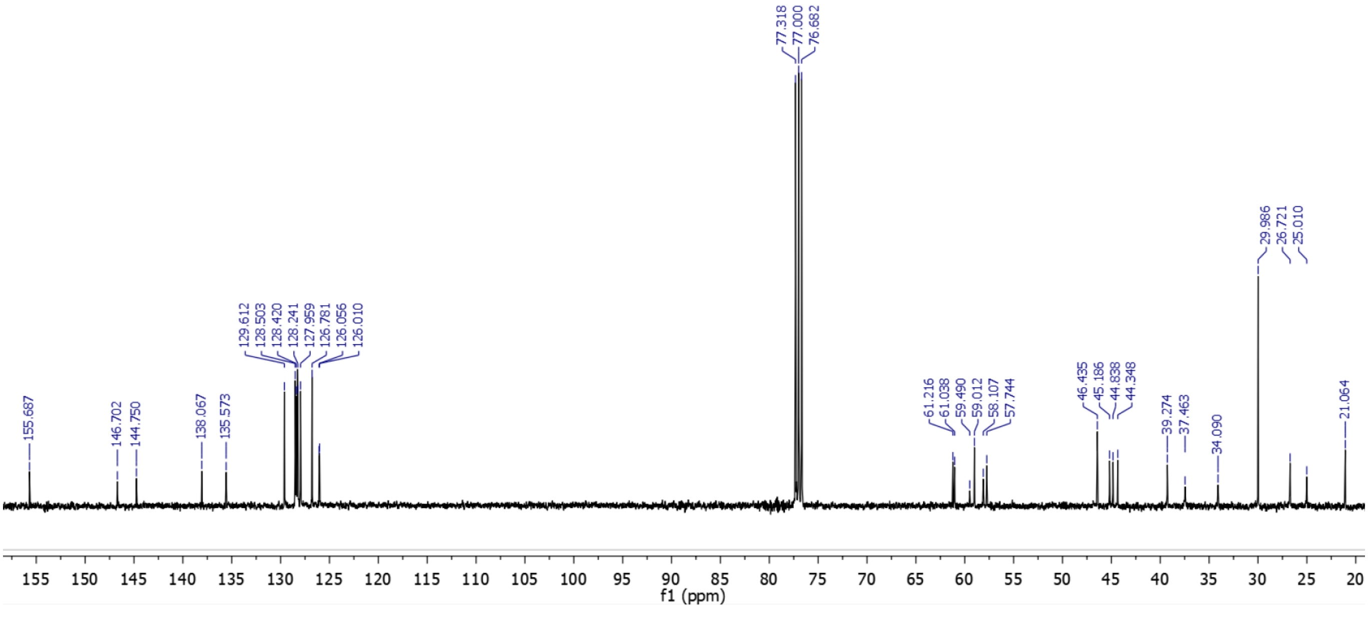

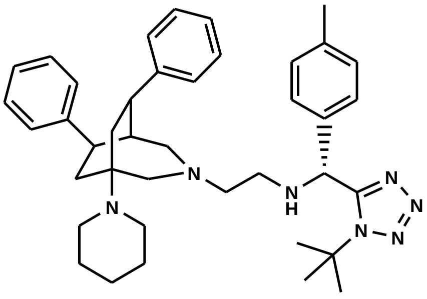

13A

Ionisation: **DI-EI**

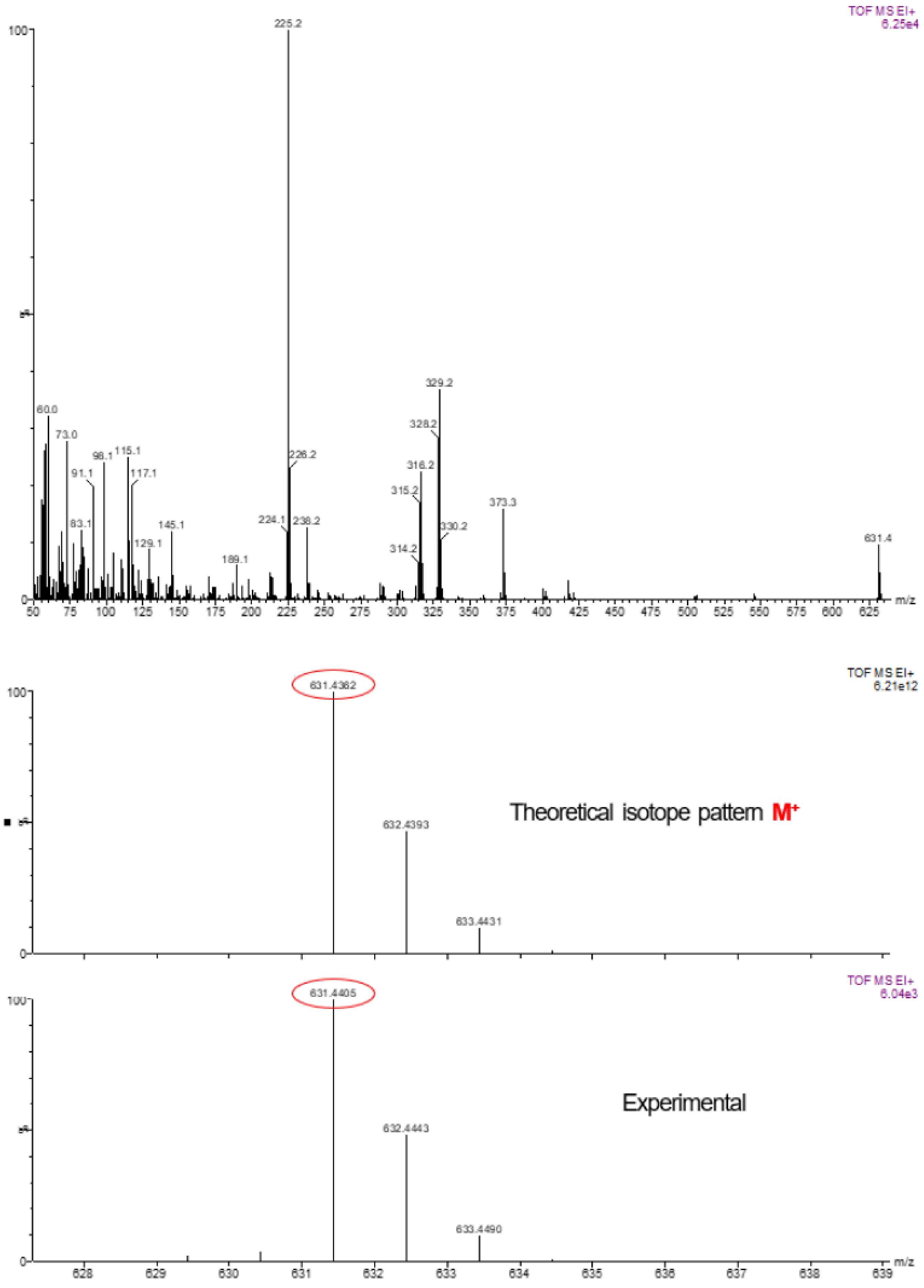

Figure S11. ^{1}H NMR at 400 MHz, ^{13}C NMR at 100 MHz spectra, and MS spectra for compound **13B**

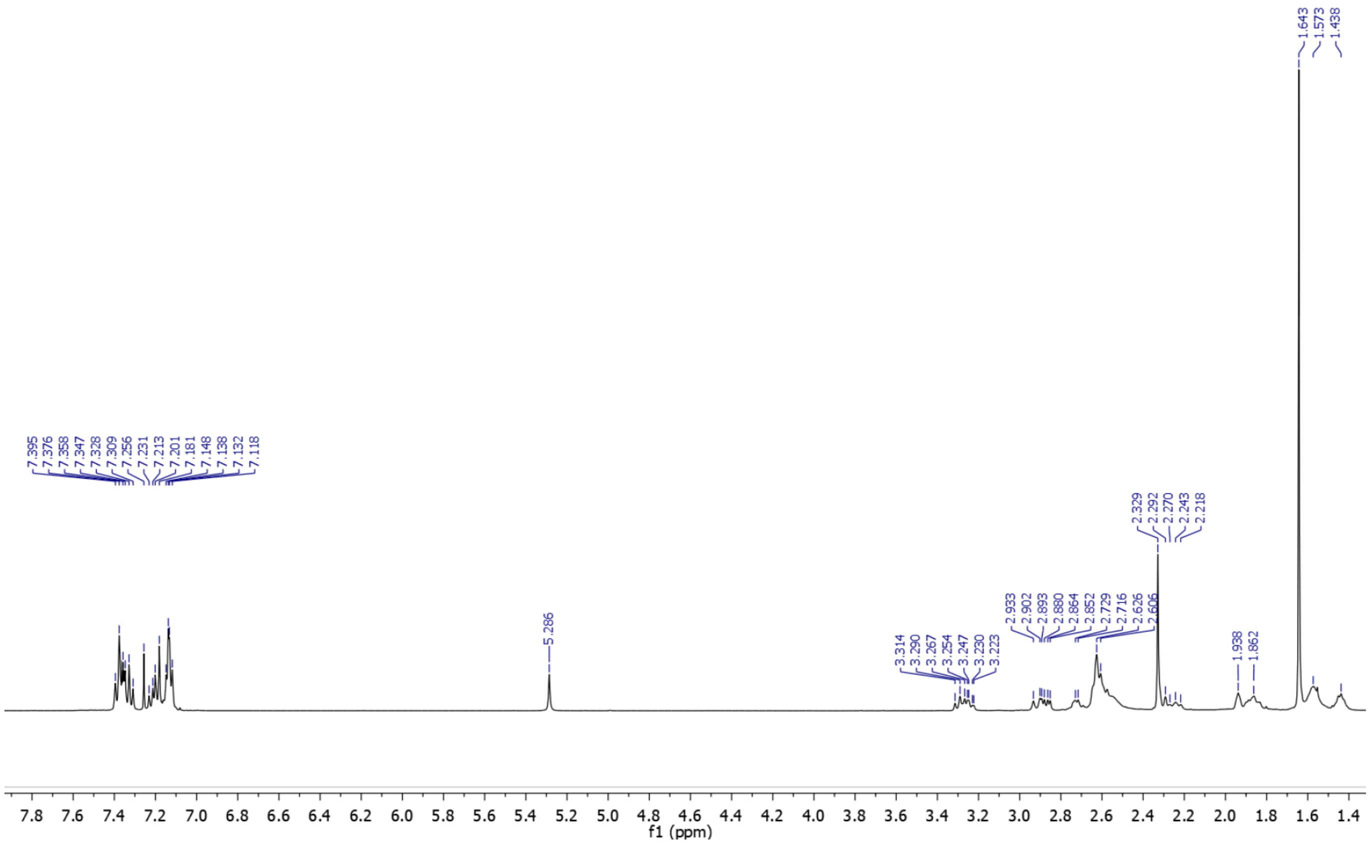

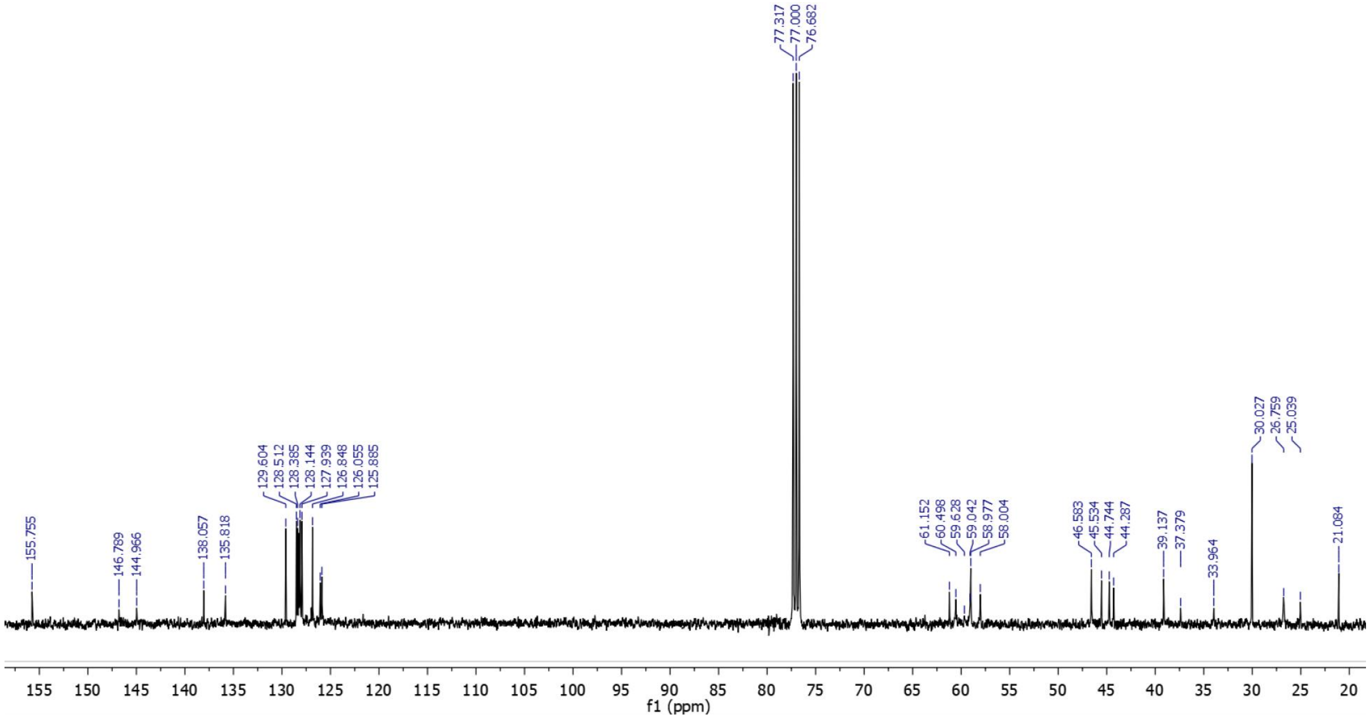

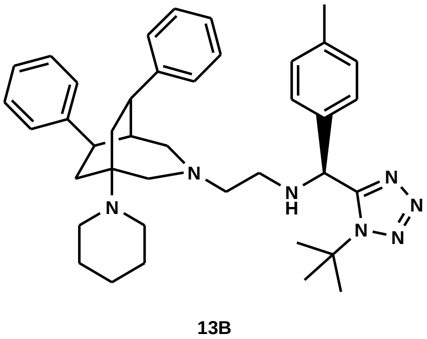

13B

Ionisation: **DI-EI**

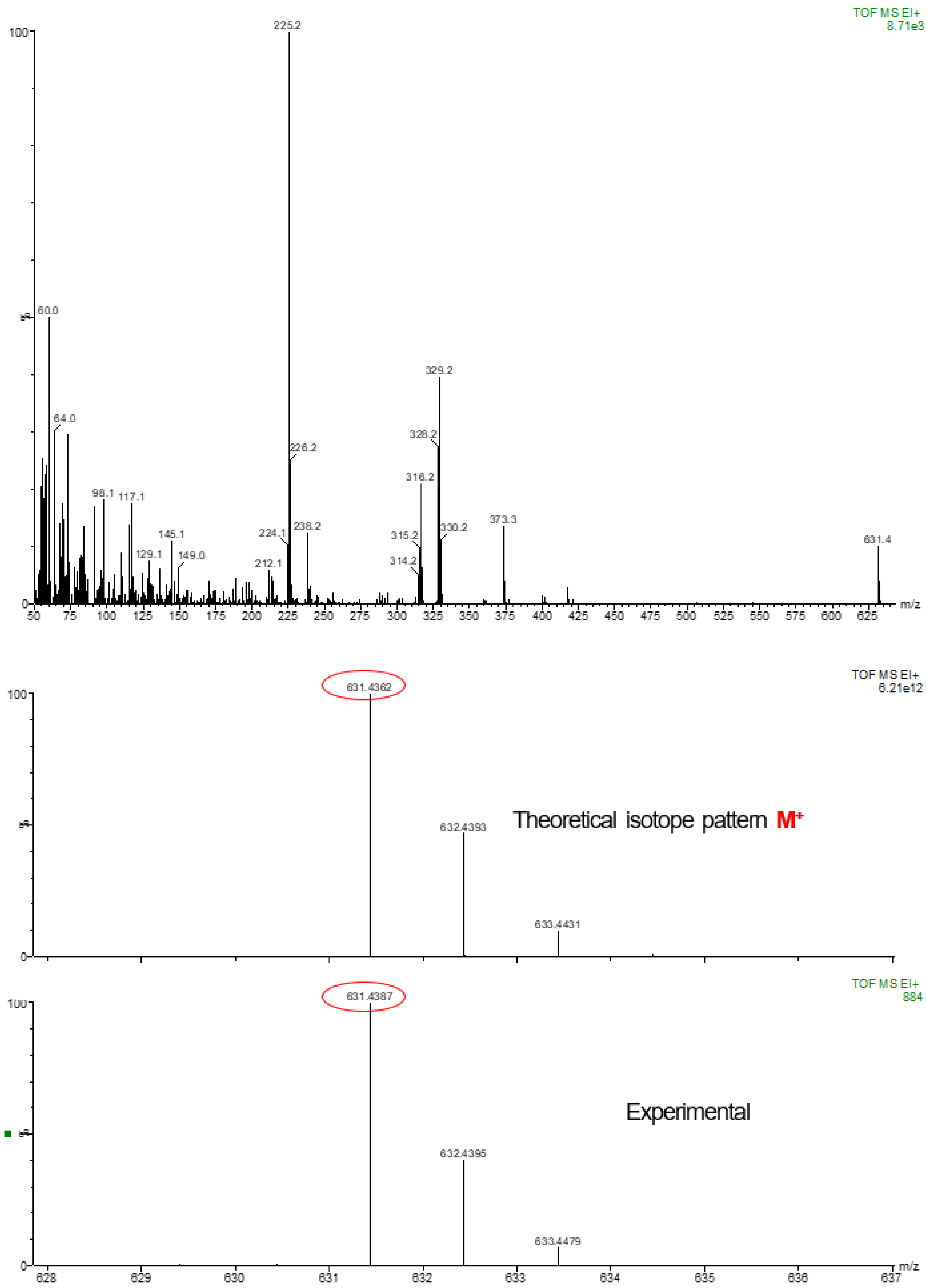

Figure S12. ^{1}H NMR at 400 MHz, ^{13}C NMR at 100 MHz spectra, and MS spectra for compound **14**

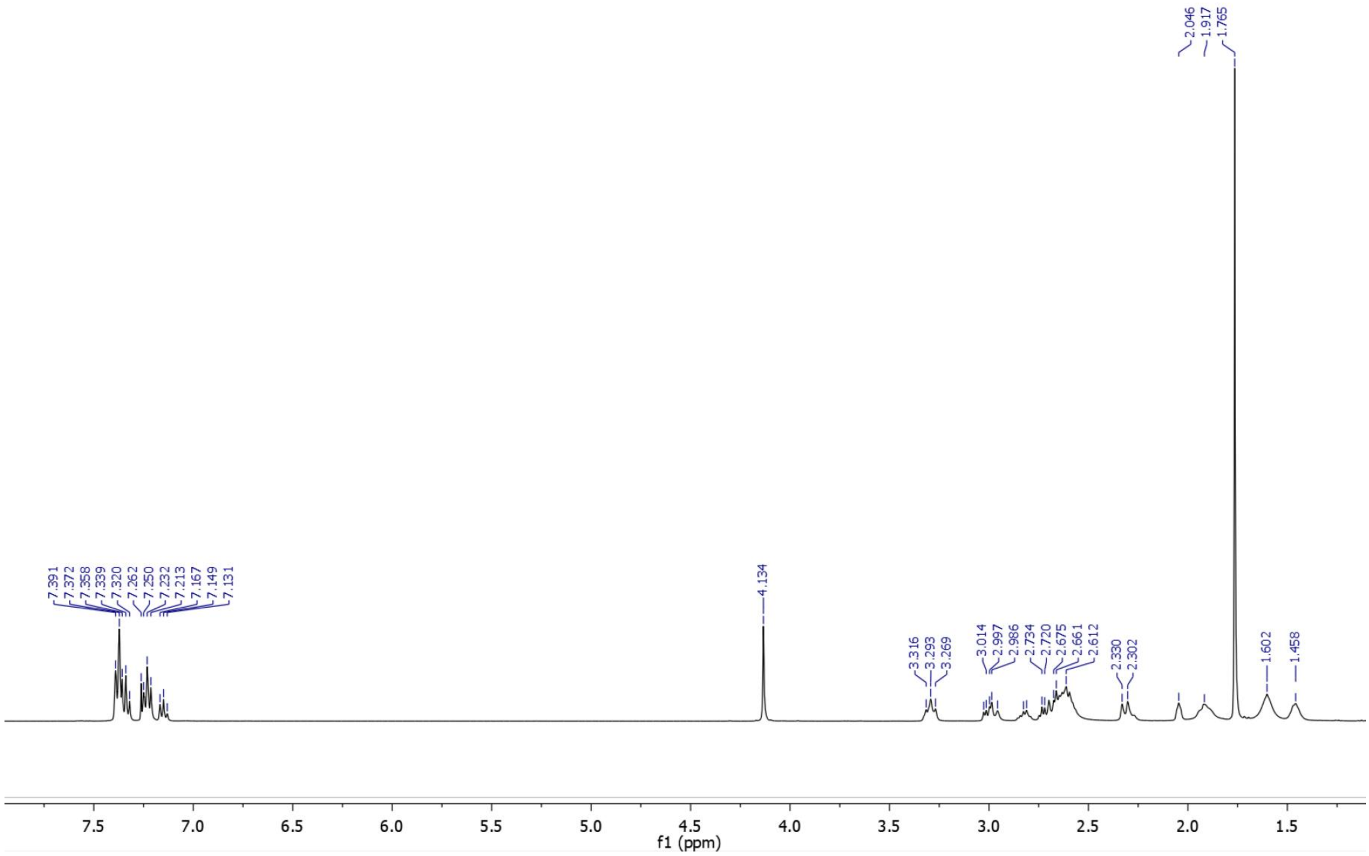

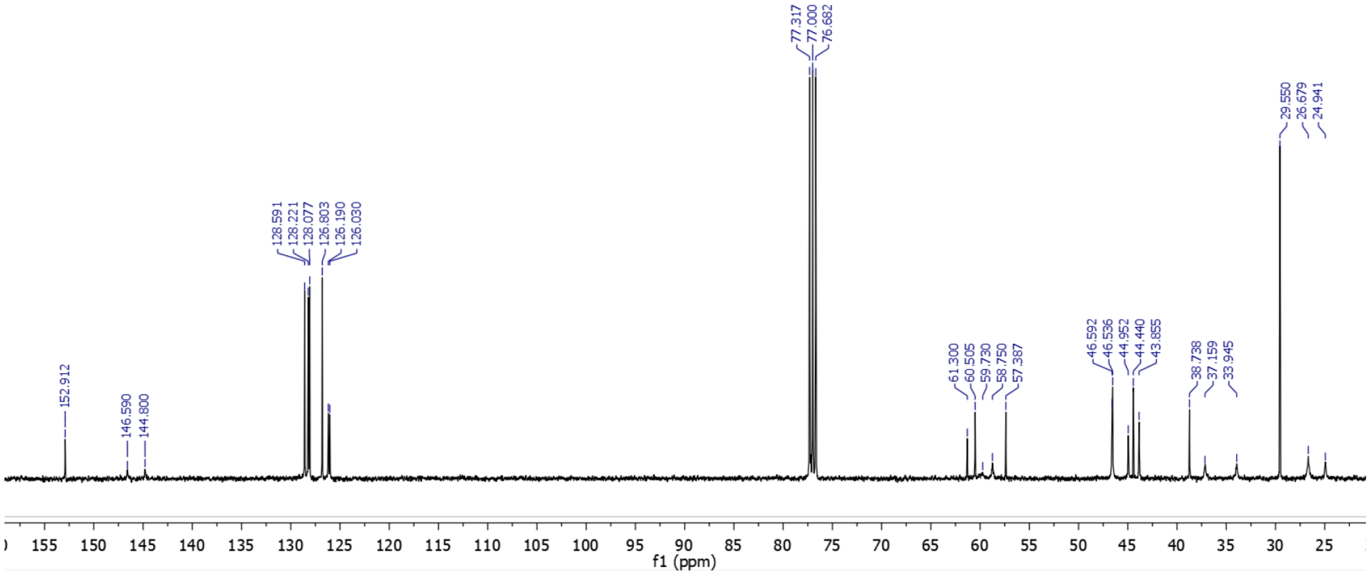

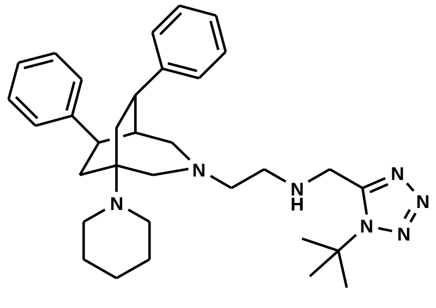

14

Ionisation: **DI-EI**

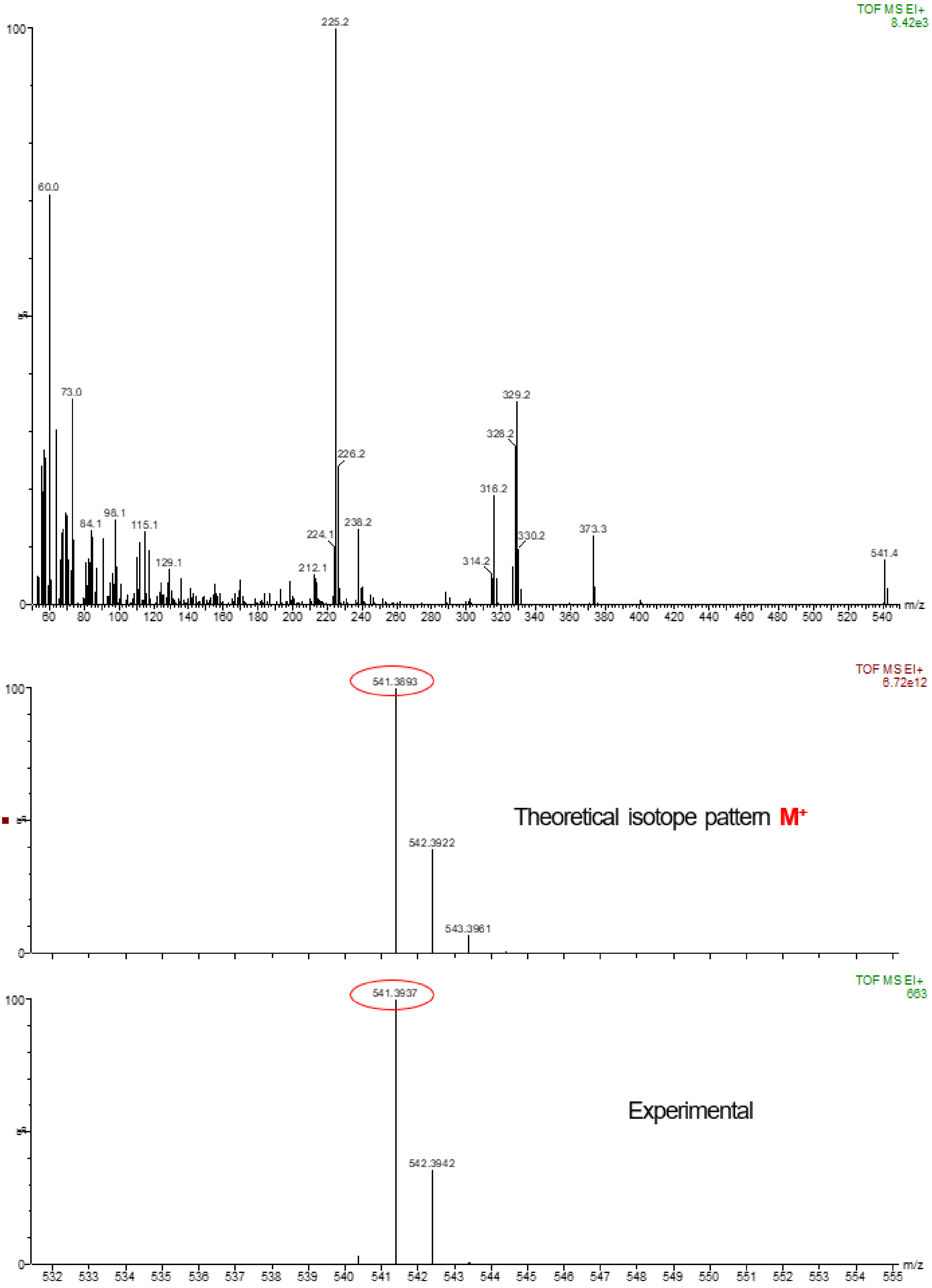

Figure S13. ^{1}H NMR at 400 MHz, ^{13}C NMR at 100 MHz spectra, and MS spectra for compound **15**

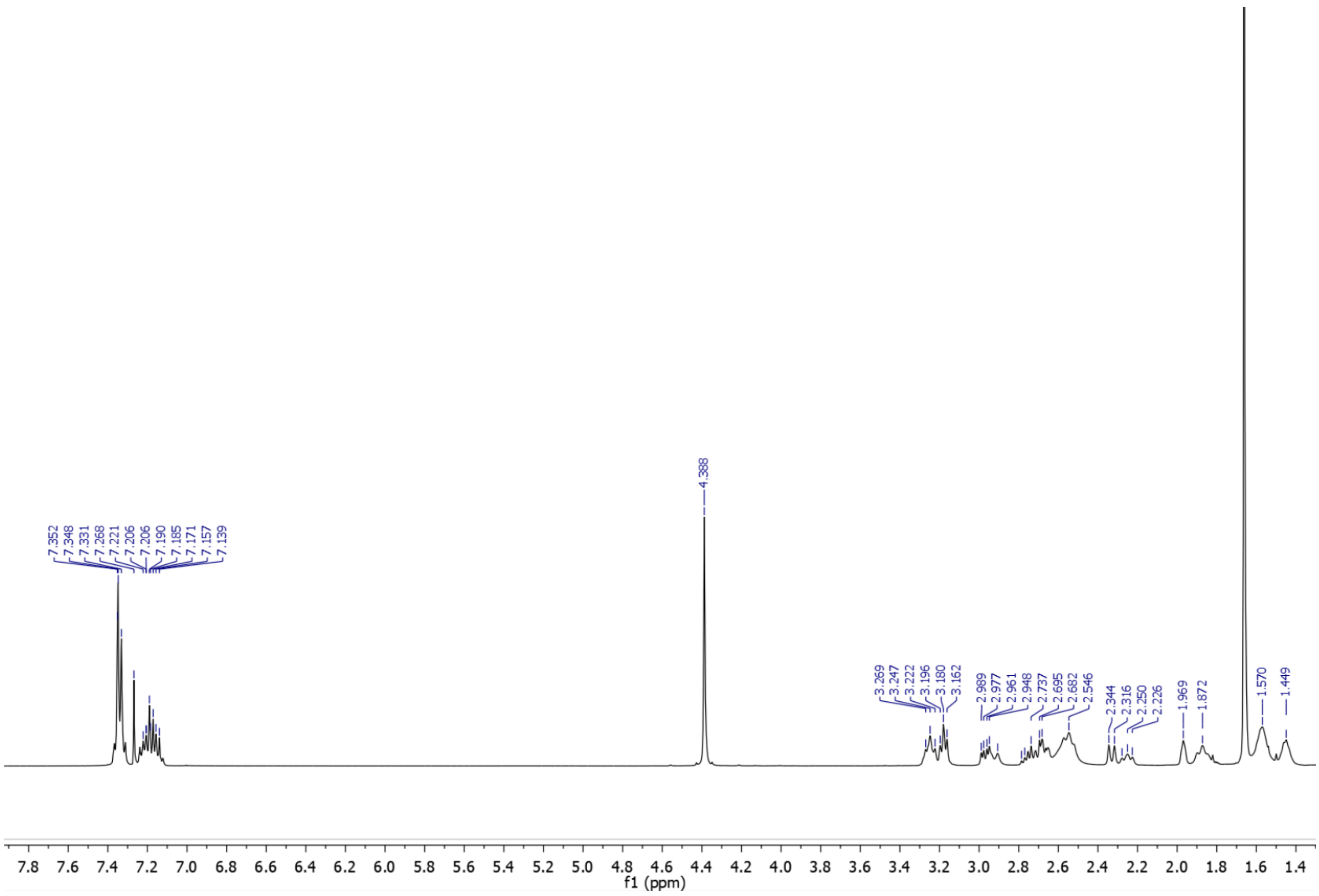

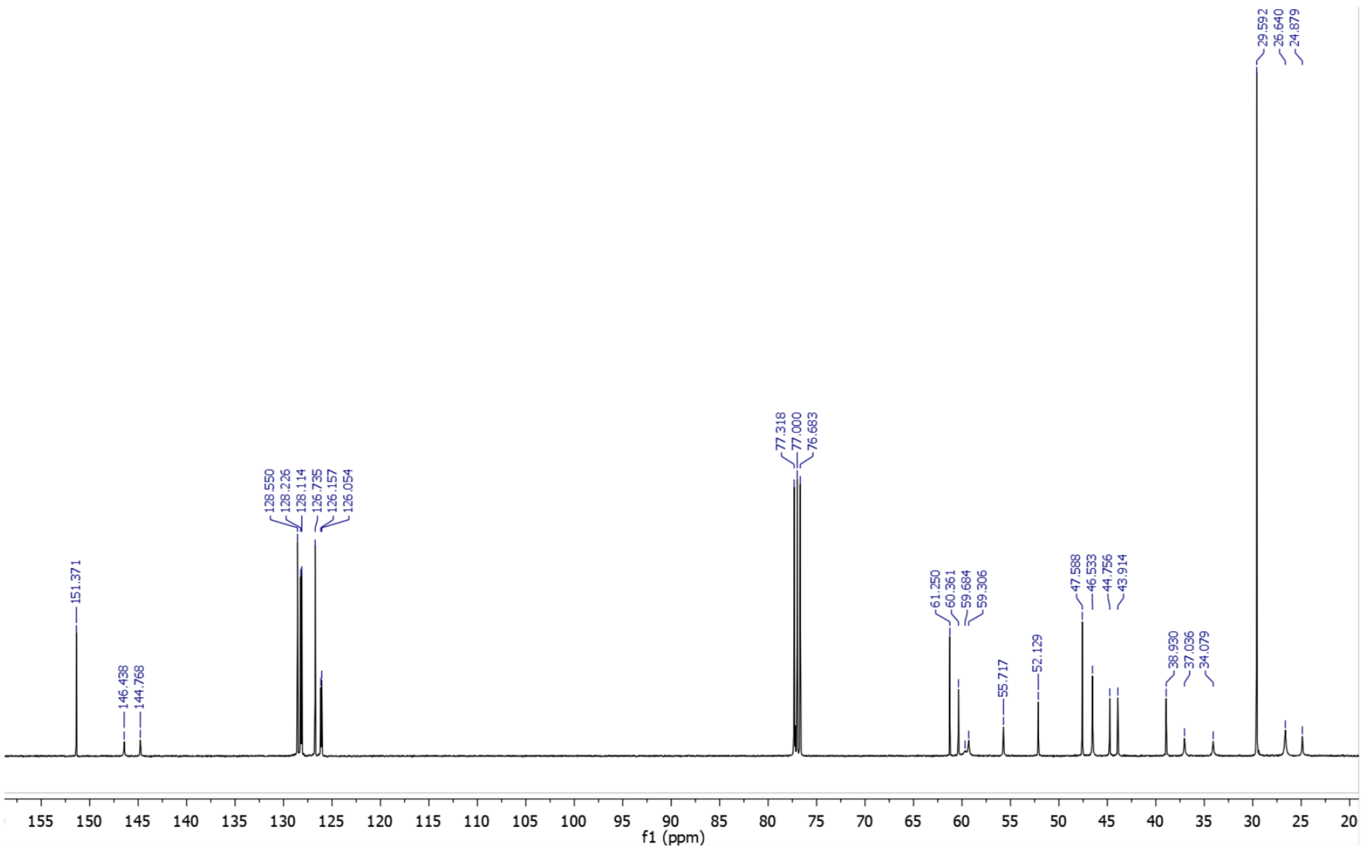

Ionisation: **DI-EI**

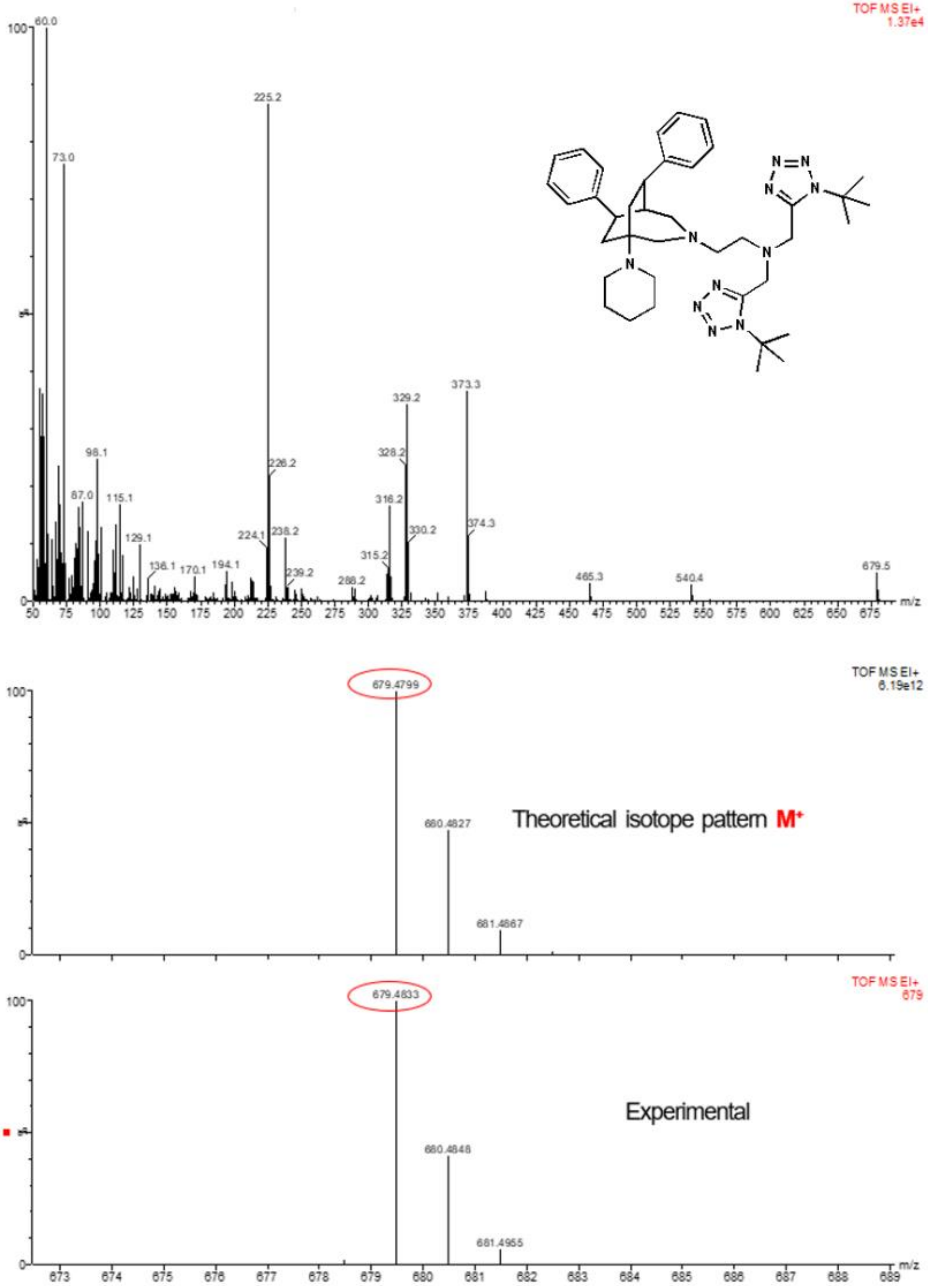

Figure S14. ^{1}H NMR at 400 MHz, ^{13}C NMR at 100 MHz spectra, and MS spectra for compound **18**

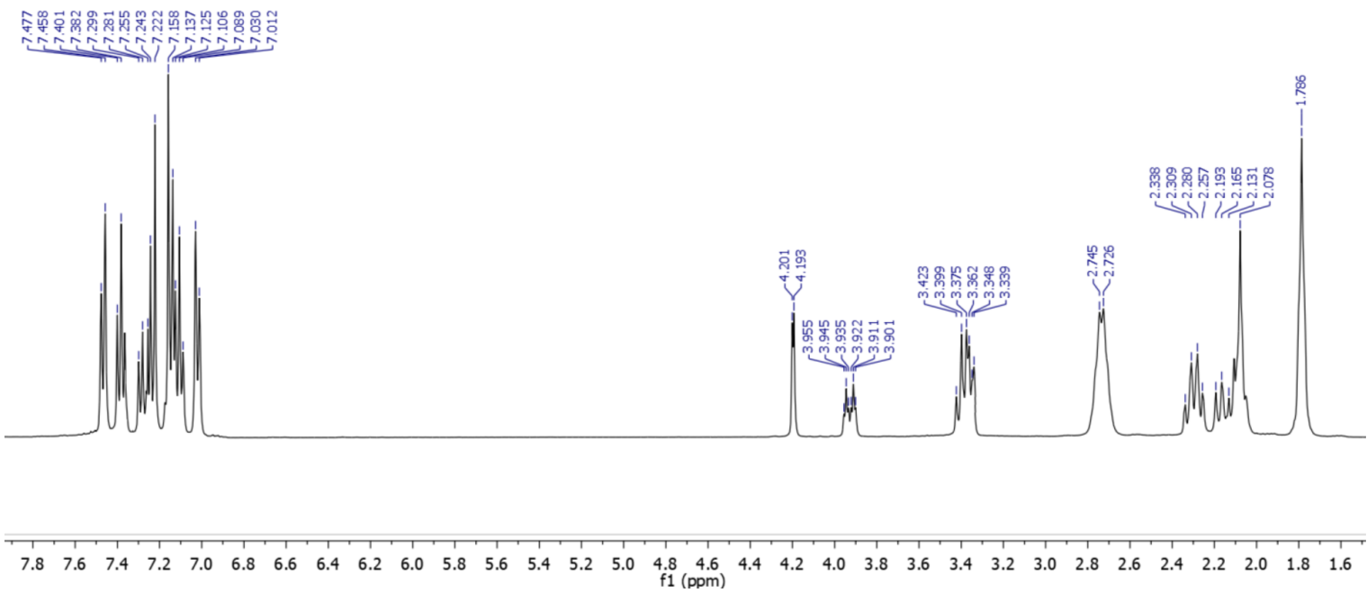

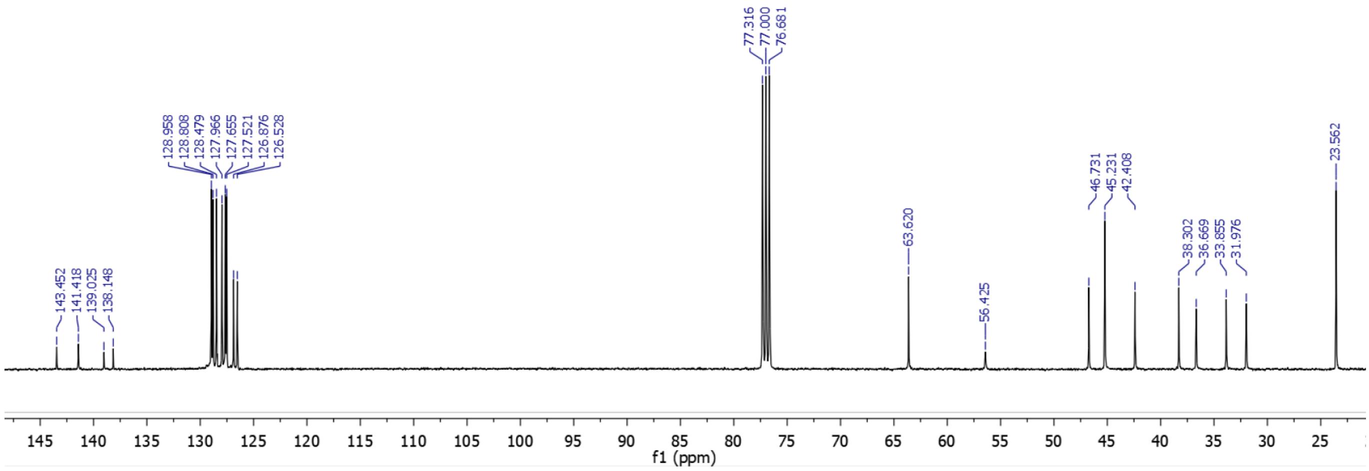

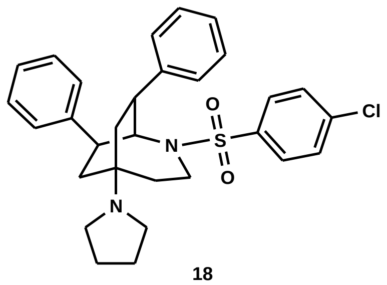

18

Ionisation: **DI-EI**

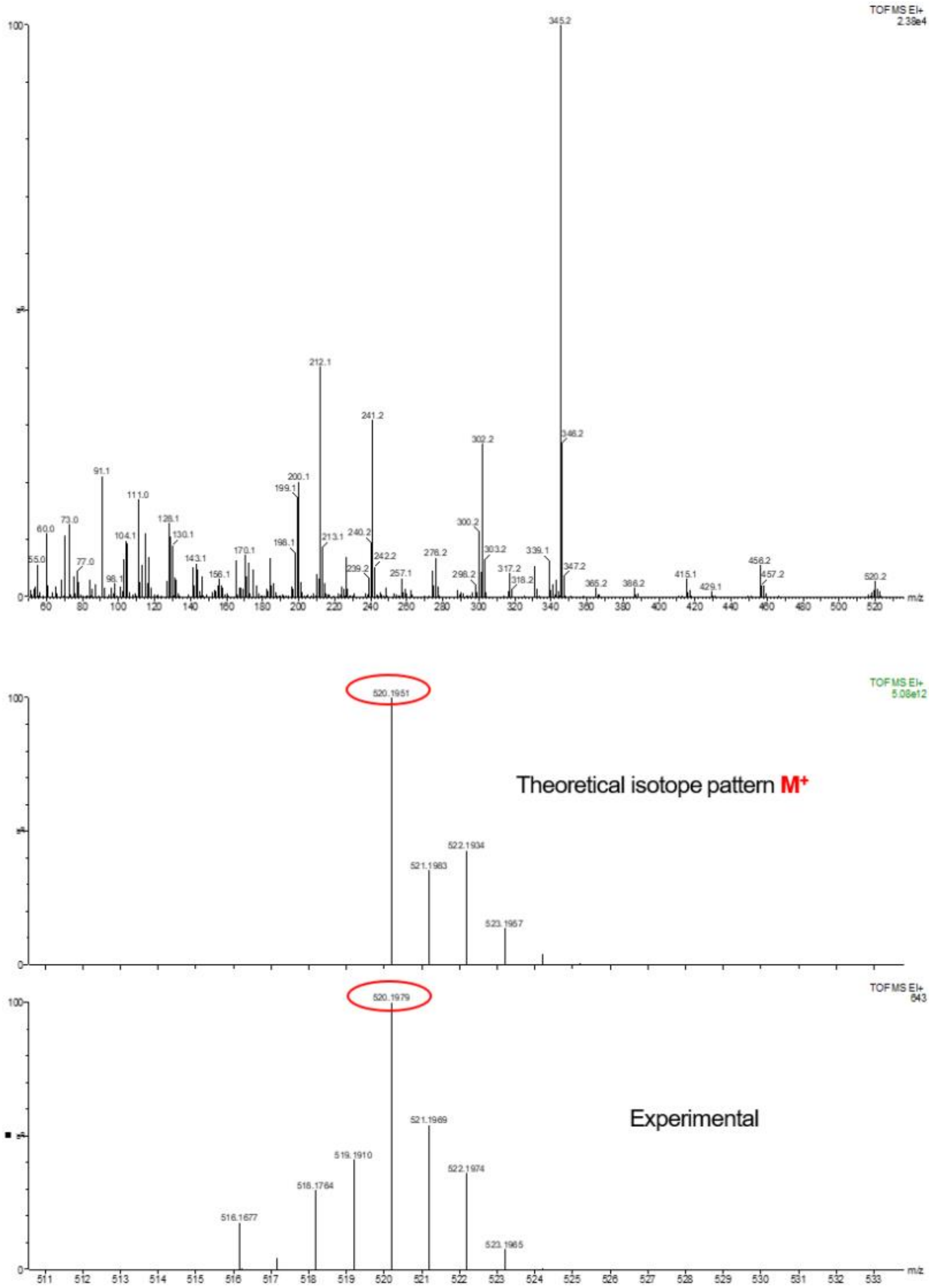

Figure S15. ^{1}H NMR at 400 MHz, ^{13}C NMR at 100 MHz spectra, and MS spectra for compound **19**

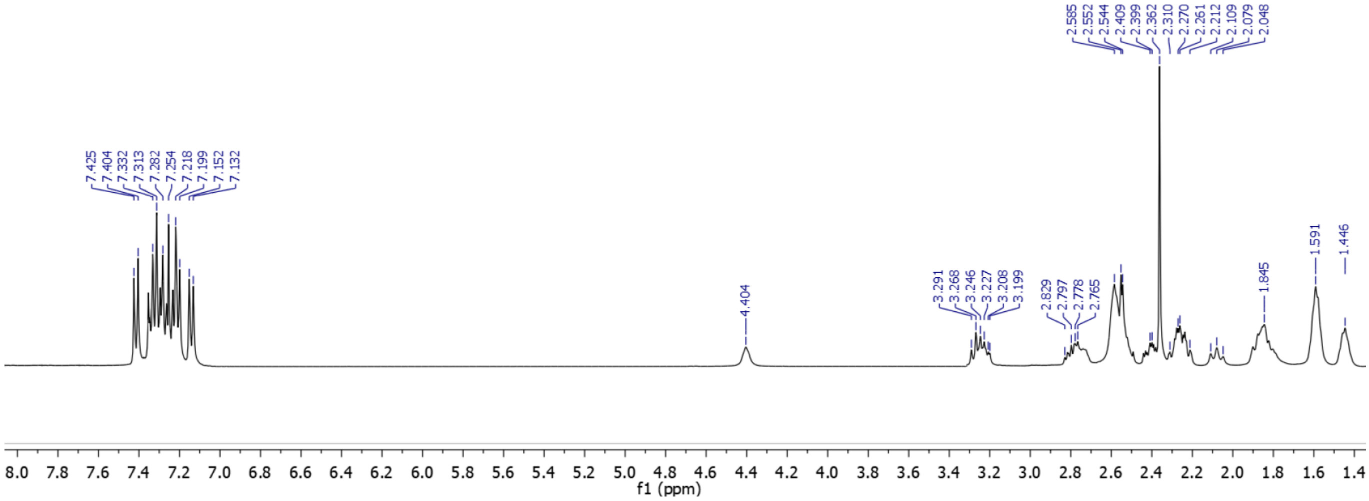

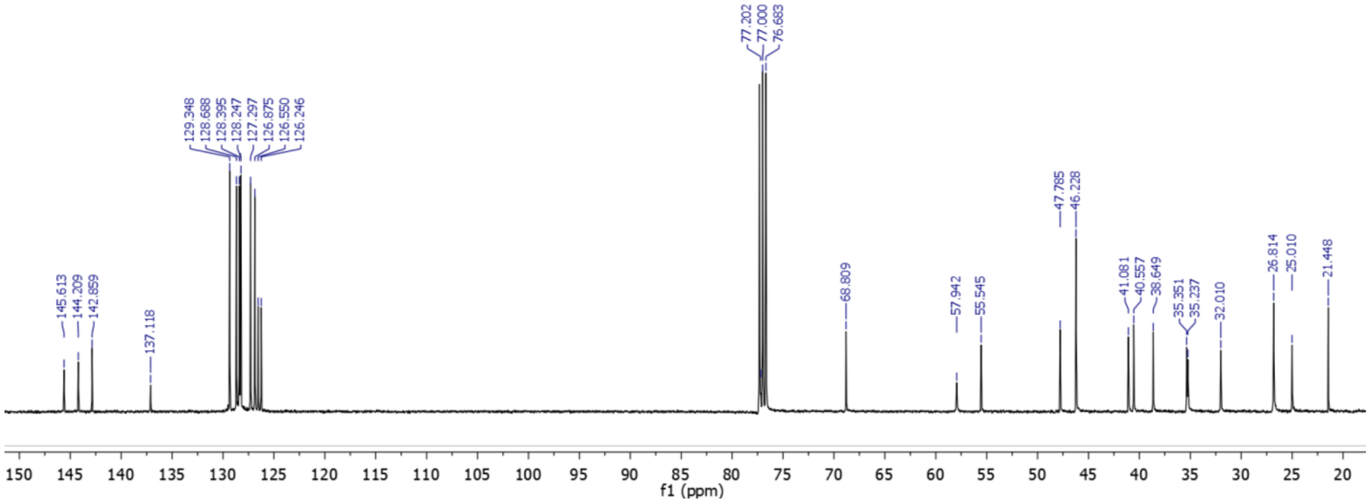

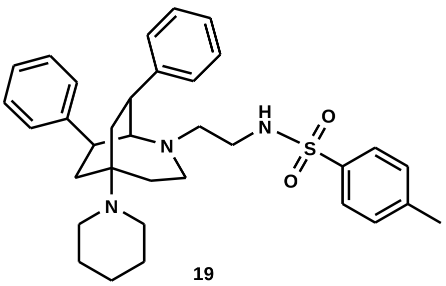

19

Ionisation: **DI-EI**

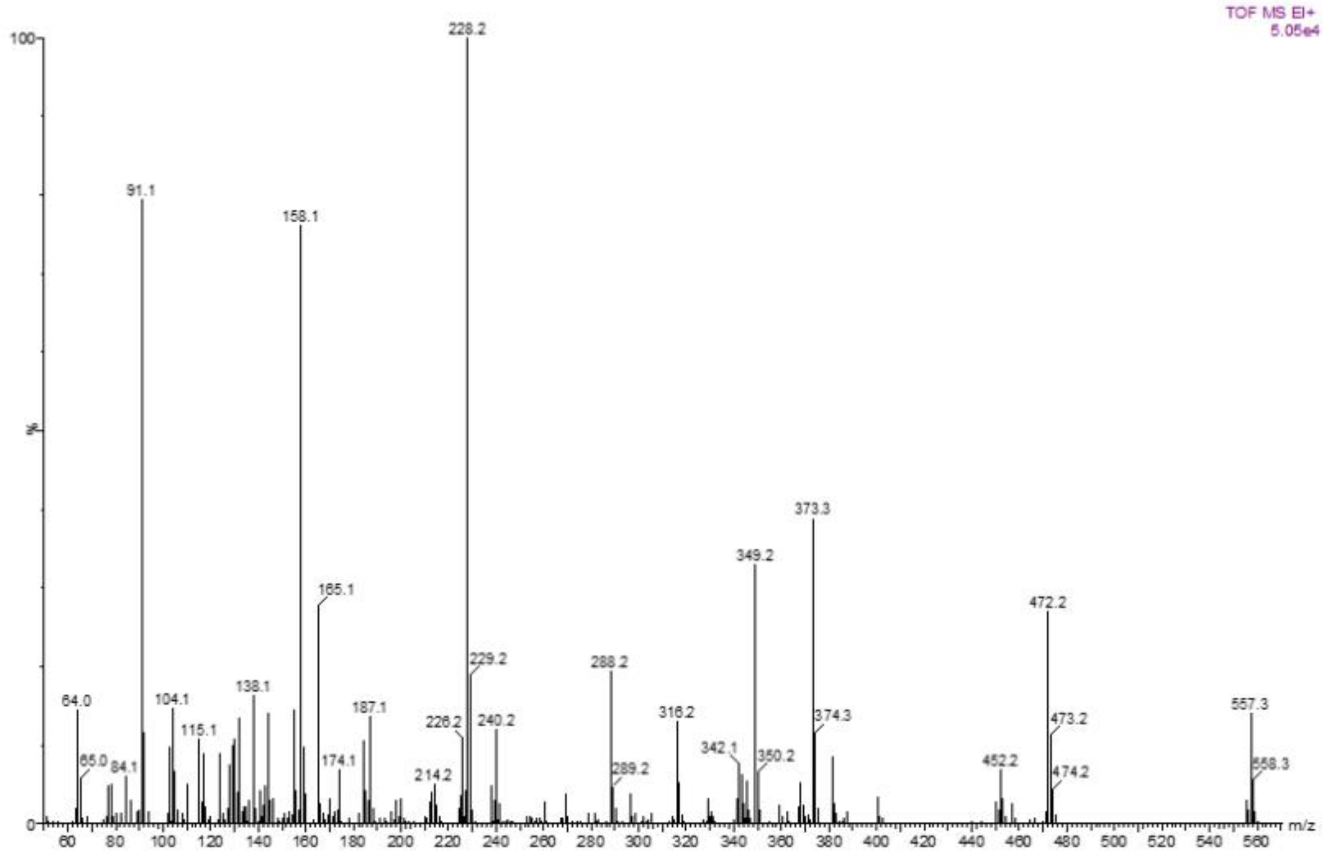

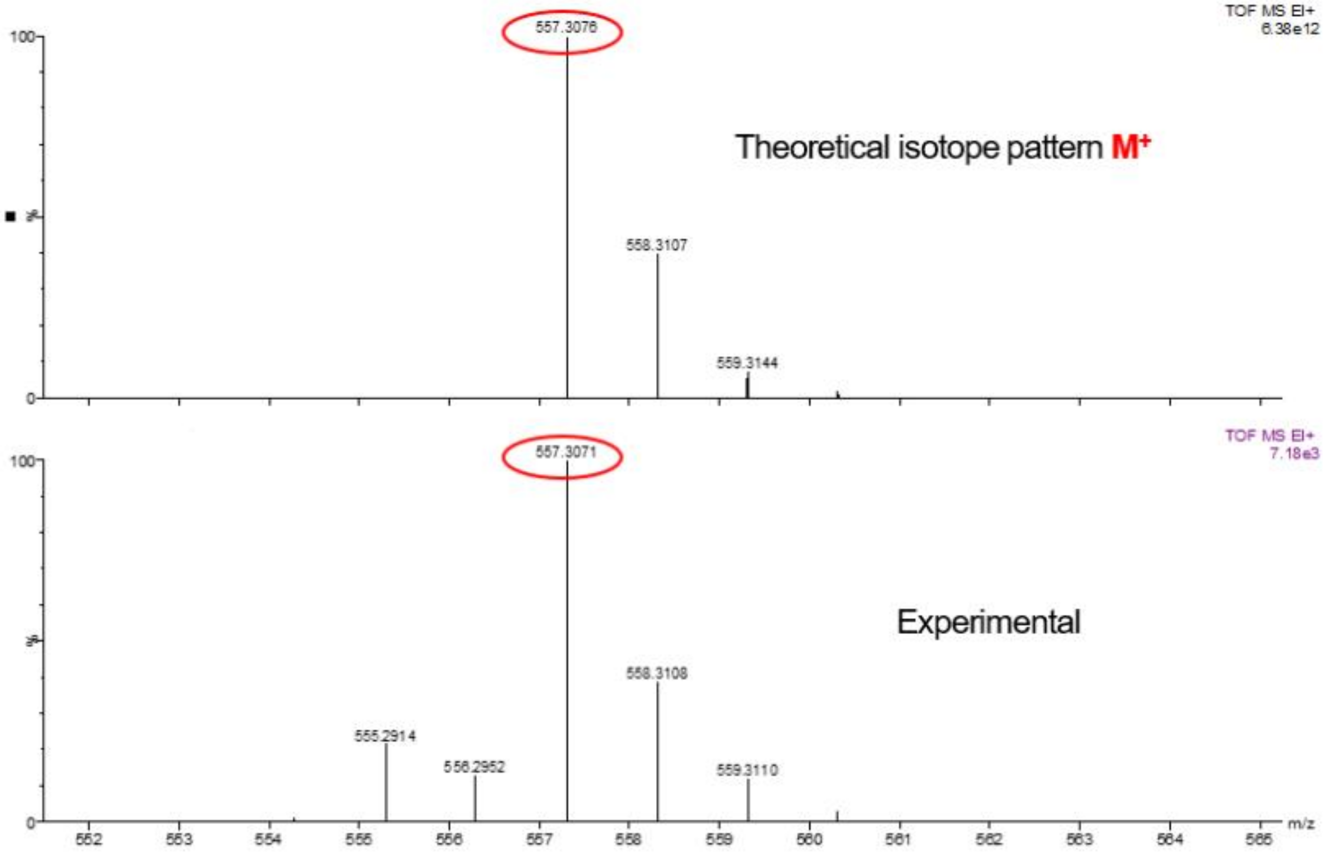

Figure S16. ^{1}H NMR at 400 MHz, ^{13}C NMR at 100 MHz spectra, and MS spectra for compound **20**

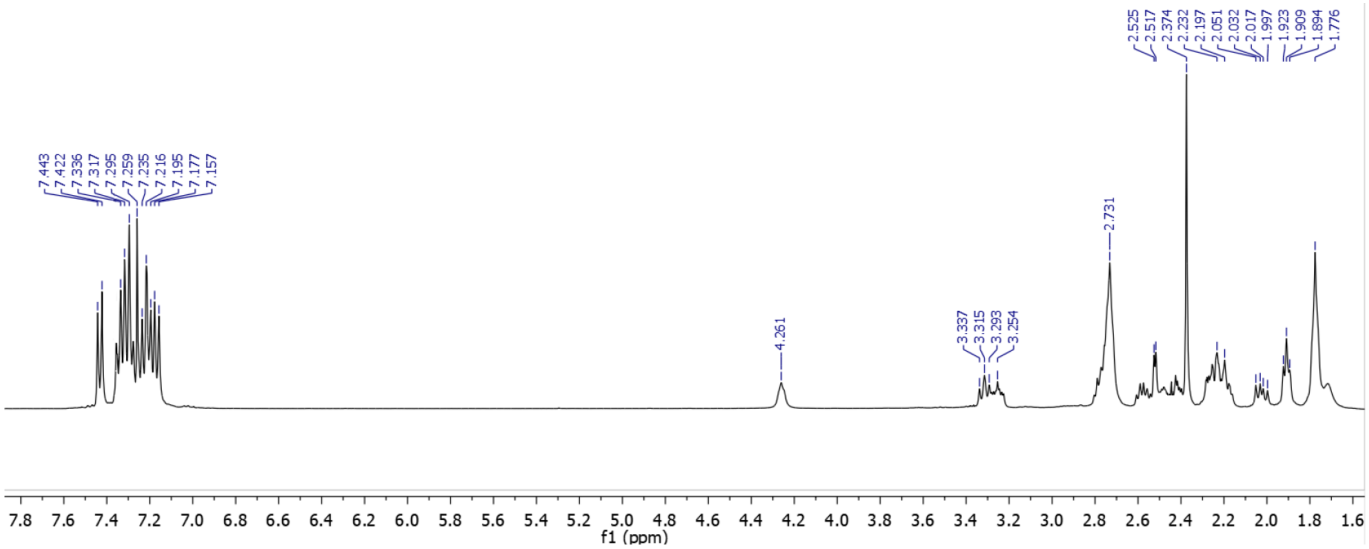

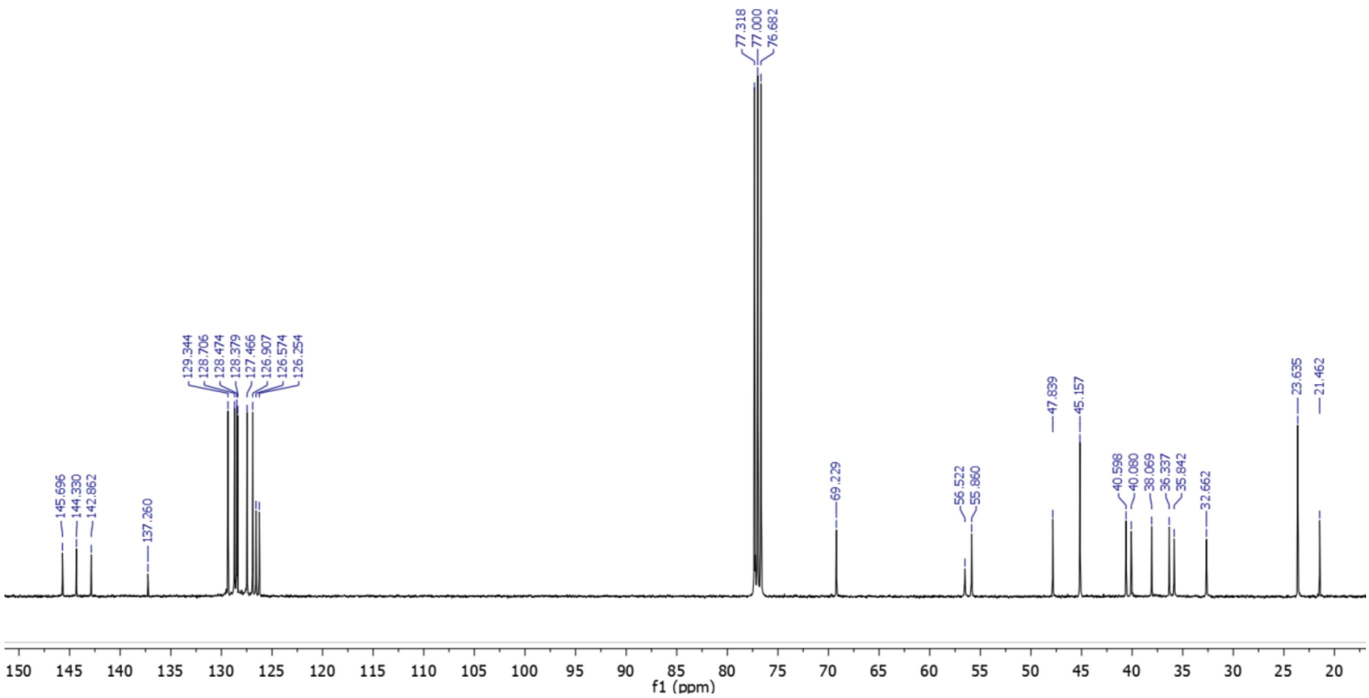

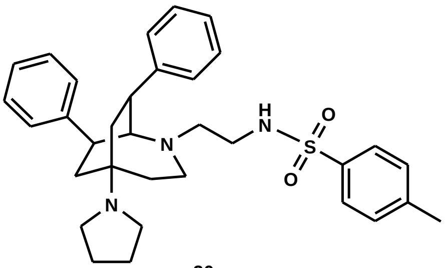

20

Ionisation: **DI-EI**

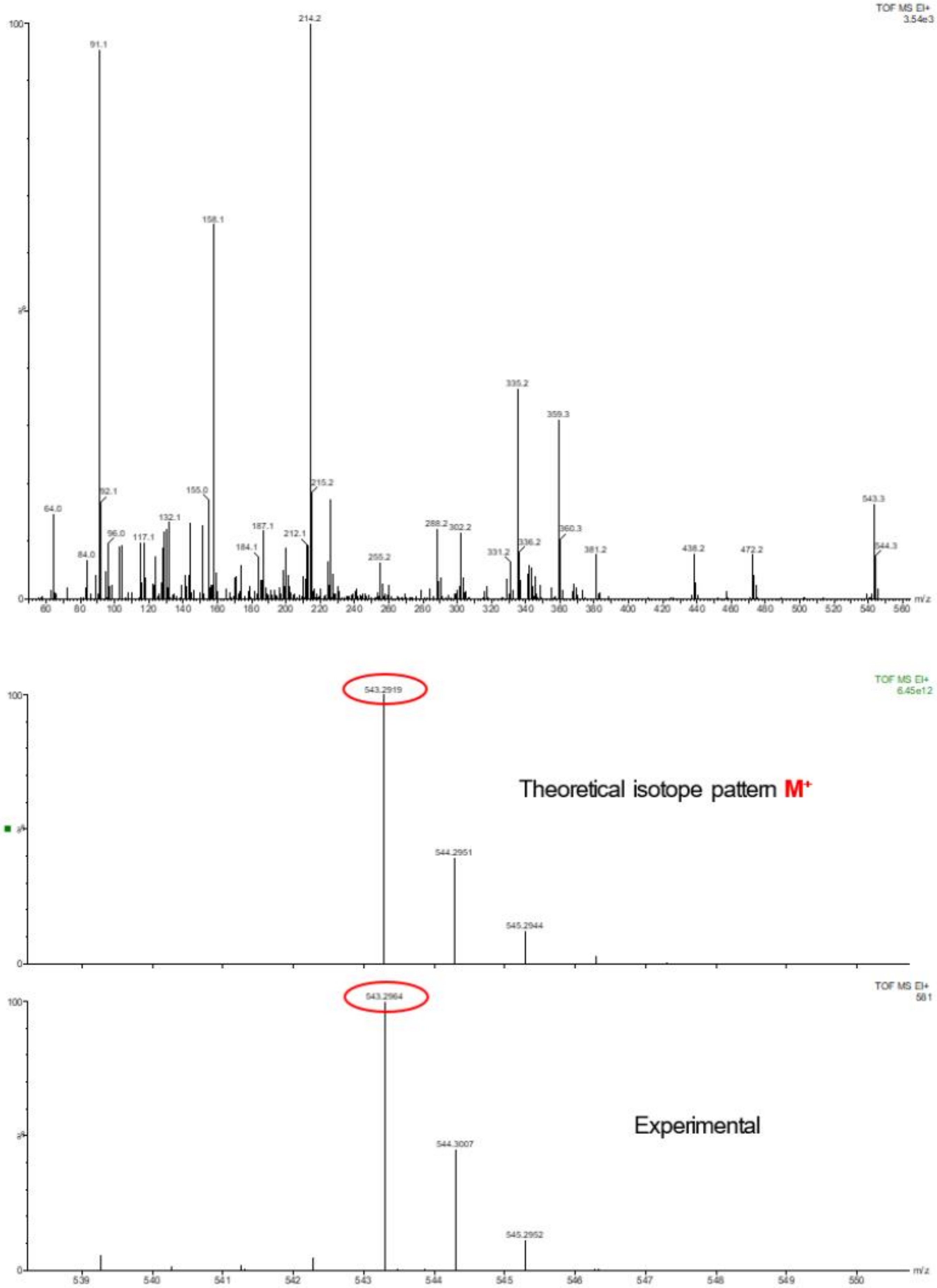

Table S1. Crystal data and structure refinement for **12B**.

Crystal data	
Identification code	ELEI3
Empirical formula	$C_{39}H_{51}N_7$
Formula weight	617.86
Crystal description	needle, colourless
Crystal size	0.18 x 0.06 x 0.04mm
Crystal system, space group	monoclinic, C 2
Unit cell dimensions:	a 28.755(2)Å
b	6.5924(5)Å
c	22.8112(16)Å
β	128.182(8)°
Volume	3399.0(5)$Å^3$
Z	4
Calculated density	1.207Mg/m^3
F(000)	1336
Linear absorption coefficient μ	0.073mm^{-1}
Absorption correction	semi-empirical from equivalents
Max. and min. transmission	0.745 and 0.631
Unit cell determination	3.04° < Θ < 27.15°
	9784 reflections used at 100K
Data collection	
Temperature	100K
Diffractometer	Bruker APEX-II CCD
Radiation source	Incoatec microfocus sealed tube
Radiation and wavelength	MoK_{α}, 0.71073Å
Monochromator	multilayer monochromator
Scan type	ϕ and ω scans
Θ range for data collection	1.80 to 27.00°
Reflections collected / unique	26894 / 7256
Significant unique reflections	5244 with I > 2σ(I)
R(int), R(sigma)	0.0772, 0.1109
Completeness to Θ = 27.0°	99.6%
Refinement	
Refinement method	Full-matrix least-squares on F^2
Data / parameters / restraints	7256 / 443 / 1
Goodness-of-fit on F^2	1.007
Final R indices [I > 2σ(I)]	R1 = 0.0513, wR2 = 0.0902
R indices (all data)	R1 = 0.0878, wR2 = 0.1010
Extinction expression	none
Weighting scheme	$w = 1/[\sigma^2(F_o^2)+(aP)^2+bP]$ where $P = (F_o^2+2F_c^2)/3$
Weighting scheme parameters a, b	0.0194, 0.0000
Largest Δ/σ in last cycle	0.000
Largest difference peak and hole	0.215 and -0.223e/$Å^3$
Structure Solution Program	SHELXS-97 (Sheldrick, 2008)
Structure Refinement Program	SHELXL-2014/6 (Sheldrick, 2015)

Table S2. Hydrogen bond for **12B** [Å, °].

D-H···A	d(D-H)	d(H···A)	d(D···A)	<(DHA)
N30-H30···N44	0.95(3)	2.45(3)	2.841(4)	104(2)

Table S3. Atomic coordinates and equivalent isotropic displacement parameters (Å^2) for **12B**. U_{eq} is defined as one third of the trace of the orthogonalized U_{ij} tensor.

	x	y	z	U_{eq}
C1	0.48894(12)	0.3016(5)	0.83153(15)	0.0133(6)
C2	0.45904(12)	0.4692(5)	0.84614(15)	0.0156(7)
N3	0.39426(10)	0.4886(4)	0.78884(12)	0.0136(5)
C4	0.37402(13)	0.5452(5)	0.71356(15)	0.0159(7)
C5	0.40504(12)	0.4369(5)	0.68687(15)	0.0151(7)
C6	0.46866(12)	0.5220(5)	0.72692(14)	0.0140(7)
C7	0.51444(13)	0.3904(5)	0.79504(15)	0.0159(7)
C8	0.44356(12)	0.1401(5)	0.77771(14)	0.0134(7)
C9	0.40741(12)	0.2041(5)	0.69472(15)	0.0148(7)
N11	0.53870(10)	0.1999(4)	0.90264(12)	0.0151(6)
C12	0.51885(13)	0.0617(5)	0.93358(15)	0.0186(7)
C13	0.57042(14)	-0.0611(5)	0.99788(16)	0.0247(8)
C14	0.61930(14)	0.0755(6)	1.05939(16)	0.0283(9)
C15	0.63637(13)	0.2308(5)	1.02668(16)	0.0226(8)
C16	0.58197(13)	0.3427(5)	0.96159(15)	0.0198(7)
N30	0.28884(10)	0.4727(4)	0.79336(13)	0.0161(6)
C31	0.30768(13)	0.6484(5)	0.77364(16)	0.0174(7)
C32	0.37387(12)	0.6420(5)	0.81529(16)	0.0169(7)
C40	0.22724(12)	0.4874(5)	0.76608(15)	0.0153(7)
N41	0.19388(11)	0.2878(4)	0.83137(13)	0.0176(6)
N42	0.19458(11)	0.0905(4)	0.84785(14)	0.0242(6)
N43	0.21596(12)	-0.0109(4)	0.82043(14)	0.0247(7)
N44	0.22977(11)	0.1170(4)	0.78624(13)	0.0206(6)
C45	0.21614(13)	0.3013(5)	0.79354(15)	0.0159(7)
C41	0.17354(13)	0.4478(5)	0.85851(17)	0.0204(7)
C42	0.14202(16)	0.3410(6)	0.88446(19)	0.0323(9)
C43	0.22860(14)	0.5591(5)	0.92347(16)	0.0265(8)
C44	0.13028(14)	0.5898(5)	0.79444(17)	0.0259(8)
C51	0.18147(13)	0.5129(5)	0.68105(15)	0.0166(7)
C52	0.15791(13)	0.7023(5)	0.65127(16)	0.0196(7)
C53	0.12003(13)	0.7319(6)	0.57446(17)	0.0240(8)
C54	0.10582(14)	0.5710(6)	0.52739(17)	0.0258(8)
C55	0.12907(15)	0.3819(6)	0.55646(17)	0.0292(9)
C56	0.16686(15)	0.3524(5)	0.63319(17)	0.0247(8)
C61	0.48720(12)	0.5527(5)	0.67769(15)	0.0172(7)
C62	0.49644(14)	0.7465(5)	0.66379(18)	0.0248(8)
C63	0.51796(15)	0.7789(6)	0.62382(18)	0.0297(9)
C64	0.52917(14)	0.6173(6)	0.59612(17)	0.0271(8)
C65	0.51868(15)	0.4228(6)	0.60765(18)	0.0288(9)
C66	0.49792(14)	0.3912(6)	0.64817(17)	0.0245(8)
C91	0.34816(12)	0.0960(5)	0.64549(15)	0.0141(6)
C92	0.33288(13)	-0.0141(5)	0.58399(15)	0.0169(7)
C93	0.28030(14)	-0.1215(5)	0.53981(16)	0.0211(8)
C94	0.24148(14)	-0.1215(5)	0.55670(16)	0.0219(7)
C95	0.25595(13)	-0.0113(5)	0.61773(15)	0.0192(7)
C96	0.30859(12)	0.0959(5)	0.66177(16)	0.0178(7)

Table S4. Hydrogen coordinates and isotropic displacement parameters (Å^2) for **12B**.

	x	y	z	U_{iso}
H21	0.4678	0.4412	0.8947	0.018(6)
H22	0.4774	0.6012	0.8505	0.018(6)
H41	0.3796	0.6931	0.7129	0.015(6)
H42	0.3311	0.5173	0.6773	0.015(6)
H5	0.3819	0.4677	0.6326	0.017(8)
H6	0.4697	0.6588	0.7466	0.014(8)
H71	0.5274	0.2787	0.7792	0.018(6)
H72	0.5495	0.4740	0.8319	0.018(6)
H81	0.4160	0.1150	0.7892	0.015(5)
H82	0.4645	0.0116	0.7856	0.015(5)
H9	0.4305	0.1548	0.6782	0.015(8)
H121	0.4885	-0.0315	0.8940	0.019(6)
H122	0.5006	0.1411	0.9514	0.019(6)
H131	0.5561	-0.1502	1.0186	0.024(6)
H132	0.5866	-0.1485	0.9790	0.024(6)
H141	0.6055	0.1456	1.0844	0.031(7)
H142	0.6543	-0.0075	1.0971	0.031(7)
H151	0.6564	0.1618	1.0093	0.030(6)
H152	0.6644	0.3295	1.0657	0.030(6)
H161	0.5633	0.4185	0.9796	0.027(7)
H162	0.5941	0.4418	0.9408	0.027(7)
H30	0.2925(13)	0.345(5)	0.7772(15)	0.016(8)
H311	0.2971	0.7746	0.7864	0.025(6)
H312	0.2870	0.6484	0.7193	0.025(6)
H321	0.3869	0.7771	0.8117	0.018(6)
H322	0.3935	0.6161	0.8687	0.018(6)
H40	0.2243	0.6081	0.7902	0.016(8)
H421	0.1103	0.2554	0.8441	0.041(6)
H422	0.1252	0.4426	0.8979	0.041(6)
H423	0.1704	0.2566	0.9280	0.041(6)
H431	0.2556	0.4618	0.9629	0.038(6)
H432	0.2169	0.6619	0.9432	0.038(6)
H433	0.2485	0.6249	0.9059	0.038(6)
H441	0.1501	0.6572	0.7771	0.040(6)
H442	0.1163	0.6919	0.8116	0.040(6)
H443	0.0965	0.5117	0.7534	0.040(6)
H52	0.1677	0.8134	0.6836	0.032(4)
H53	0.1040	0.8626	0.5545	0.032(4)
H54	0.0800	0.5908	0.4749	0.032(4)
H55	0.1193	0.2712	0.5240	0.032(4)
H56	0.1828	0.2215	0.6530	0.032(4)
H62	0.4880	0.8596	0.6817	0.035(4)
H63	0.5249	0.9132	0.6158	0.035(4)
H64	0.5440	0.6394	0.5693	0.035(4)
H65	0.5256	0.3104	0.5879	0.035(4)
H66	0.4909	0.2566	0.6559	0.035(4)
H92	0.3591	-0.0158	0.5719	0.022(4)
H93	0.2708	-0.1953	0.4979	0.022(4)
H94	0.2055	-0.1960	0.5269	0.022(4)
H95	0.2295	-0.0092	0.6295	0.022(4)
H96	0.3179	0.1702	0.7035	0.022(4)

Table S5. Anisotropic displacement parameters (Å^2) for **12B**. The anisotropic displacement factor exponent takes the form: $-2\pi^2[h^2 a^{*2}U_{11} + ... + 2 h k a^* b^* U_{12}]$.

	U_{11}	U_{22}	U_{33}	U_{23}	U_{13}	U_{12}
C1	0.0095(15)	0.0172(17)	0.0110(14)	0.0002(12)	0.0052(13)	-0.0007(12)
C2	0.0132(16)	0.0181(17)	0.0154(15)	-0.0032(13)	0.0089(13)	-0.0040(13)
N3	0.0101(13)	0.0180(15)	0.0114(12)	-0.0023(11)	0.0059(11)	0.0002(11)
C4	0.0144(16)	0.0154(17)	0.0175(15)	0.0006(13)	0.0097(13)	0.0006(13)
C5	0.0143(16)	0.0199(18)	0.0105(14)	0.0005(12)	0.0074(13)	0.0005(13)
C6	0.0159(16)	0.0158(17)	0.0143(14)	-0.0028(12)	0.0113(13)	-0.0039(13)
C7	0.0136(16)	0.0205(17)	0.0167(15)	-0.0014(13)	0.0110(14)	-0.0005(13)
C8	0.0132(16)	0.0124(16)	0.0129(15)	0.0016(12)	0.0071(14)	0.0021(12)
C9	0.0141(16)	0.0194(18)	0.0136(15)	0.0000(13)	0.0099(14)	0.0026(13)
N11	0.0128(13)	0.0191(15)	0.0118(12)	0.0004(11)	0.0069(11)	-0.0001(11)
C12	0.0178(16)	0.0231(19)	0.0149(15)	0.0002(14)	0.0101(14)	-0.0021(14)
C13	0.0217(19)	0.031(2)	0.0190(17)	0.0066(15)	0.0115(16)	0.0028(15)
C14	0.0171(18)	0.044(2)	0.0156(16)	0.0057(16)	0.0062(14)	0.0012(16)
C15	0.0156(17)	0.034(2)	0.0157(16)	-0.0017(15)	0.0083(14)	-0.0025(15)
C16	0.0170(18)	0.0233(19)	0.0165(16)	-0.0037(14)	0.0091(15)	-0.0053(14)
N30	0.0118(13)	0.0166(15)	0.0189(13)	0.0020(12)	0.0090(11)	0.0014(11)
C31	0.0171(17)	0.0175(17)	0.0212(17)	0.0004(13)	0.0137(15)	-0.0002(13)
C32	0.0149(17)	0.0176(18)	0.0199(16)	-0.0013(13)	0.0116(14)	-0.0018(13)
C40	0.0115(15)	0.0196(17)	0.0166(15)	-0.0002(13)	0.0096(13)	0.0006(13)
N41	0.0179(14)	0.0173(15)	0.0213(13)	-0.0005(11)	0.0139(12)	-0.0007(11)
N42	0.0307(16)	0.0207(17)	0.0278(15)	-0.0007(13)	0.0214(14)	-0.0013(13)
N43	0.0305(17)	0.0221(17)	0.0293(15)	-0.0008(13)	0.0223(14)	-0.0020(13)
N44	0.0209(15)	0.0200(16)	0.0214(14)	0.0005(12)	0.0134(13)	-0.0022(12)
C45	0.0132(16)	0.0195(18)	0.0125(14)	-0.0005(13)	0.0067(13)	-0.0023(13)
C41	0.0226(18)	0.0231(19)	0.0259(17)	-0.0016(14)	0.0202(16)	0.0004(14)
C42	0.043(2)	0.030(2)	0.047(2)	-0.0006(18)	0.039(2)	-0.0018(18)
C43	0.0318(19)	0.026(2)	0.0251(17)	-0.0033(15)	0.0195(16)	0.0005(16)
C44	0.0196(18)	0.027(2)	0.0360(19)	0.0041(16)	0.0199(16)	0.0043(15)
C51	0.0119(16)	0.0230(19)	0.0172(15)	-0.0013(13)	0.0102(13)	-0.0017(13)
C52	0.0178(17)	0.0238(19)	0.0185(16)	-0.0004(14)	0.0119(15)	-0.0003(14)
C53	0.0183(18)	0.030(2)	0.0238(18)	0.0045(15)	0.0132(16)	0.0031(15)
C54	0.0207(18)	0.038(2)	0.0170(16)	0.0031(16)	0.0108(15)	0.0017(16)
C55	0.034(2)	0.034(2)	0.0176(17)	-0.0045(16)	0.0149(17)	0.0021(17)
C56	0.027(2)	0.024(2)	0.0237(18)	0.0031(15)	0.0163(16)	0.0064(15)
C61	0.0105(15)	0.025(2)	0.0145(15)	0.0003(14)	0.0072(13)	-0.0020(13)
C62	0.0256(19)	0.025(2)	0.0297(18)	0.0029(15)	0.0200(17)	0.0056(15)
C63	0.034(2)	0.030(2)	0.033(2)	0.0097(16)	0.0245(18)	0.0018(16)
C64	0.0222(19)	0.045(3)	0.0190(17)	0.0024(16)	0.0153(16)	-0.0024(17)
C65	0.034(2)	0.037(2)	0.0271(18)	-0.0084(16)	0.0243(18)	-0.0056(17)
C66	0.028(2)	0.026(2)	0.0286(18)	-0.0030(15)	0.0222(17)	-0.0061(16)
C91	0.0140(16)	0.0122(17)	0.0137(15)	0.0020(12)	0.0074(13)	0.0013(12)
C92	0.0206(17)	0.0178(17)	0.0150(15)	0.0032(13)	0.0123(14)	0.0030(14)
C93	0.0237(19)	0.0209(19)	0.0137(16)	-0.0040(14)	0.0091(15)	-0.0026(14)
C94	0.0161(18)	0.0197(18)	0.0195(16)	-0.0008(14)	0.0059(15)	-0.0026(14)
C95	0.0179(17)	0.0190(18)	0.0217(16)	0.0012(14)	0.0126(15)	0.0013(14)
C96	0.0178(17)	0.0194(18)	0.0166(15)	-0.0003(13)	0.0109(14)	0.0012(14)

Table S6. Full list of bond lengths [Å] and angles [°] for **12B**.

C1-N11	1.503(4)	N41-N42	1.350(4)
C1-C7	1.526(4)	N41-C45	1.359(4)
C1-C8	1.537(4)	N41-C41	1.512(4)
C1-C2	1.557(4)	N42-N43	1.301(4)
C2-N3	1.478(3)	N43-N44	1.364(4)
C2-H21	0.99	N44-C45	1.318(4)
C2-H22	0.99	C41-C44	1.520(4)
N3-C32	1.472(4)	C41-C42	1.527(4)
N3-C4	1.480(3)	C41-C43	1.530(4)
C4-C5	1.532(4)	C42-H421	0.98
C4-H41	0.99	C42-H422	0.98
C4-H42	0.99	C42-H423	0.98
C5-C9	1.542(4)	C43-H431	0.98
C5-C6	1.559(4)	C43-H432	0.98
C5-H5	1.00	C43-H433	0.98
C6-C61	1.525(4)	C44-H441	0.98
C6-C7	1.540(4)	C44-H442	0.98
C6-H6	1.00	C44-H443	0.98
C7-H71	0.99	C51-C52	1.383(4)
C7-H72	0.99	C51-C56	1.386(4)
C8-C9	1.552(4)	C52-C53	1.391(4)
C8-H81	0.99	C52-H52	0.95
C8-H82	0.99	C53-C54	1.379(5)
C9-C91	1.519(4)	C53-H53	0.95
C9-H9	1.00	C54-C55	1.376(5)
N11-C12	1.467(4)	C54-H54	0.95
N11-C16	1.475(4)	C55-C56	1.389(4)
C12-C13	1.522(4)	C55-H55	0.95
C12-H121	0.99	C56-H56	0.95
C12-H122	0.99	C61-C62	1.381(4)
C13-C14	1.522(5)	C61-C66	1.392(4)
C13-H131	0.99	C62-C63	1.399(4)
C13-H132	0.99	C62-H62	0.95
C14-C15	1.516(5)	C63-C64	1.375(5)
C14-H141	0.99	C63-H63	0.95
C14-H142	0.99	C64-C65	1.379(5)
C15-C16	1.525(4)	C64-H64	0.95
C15-H151	0.99	C65-C66	1.392(4)
C15-H152	0.99	C65-H65	0.95
C16-H161	0.99	C66-H66	0.95
C16-H162	0.99	C91-C92	1.388(4)
N30-C31	1.462(4)	C91-C96	1.398(4)
N30-C40	1.474(4)	C92-C93	1.385(4)
N30-H30	0.95(3)	C92-H92	0.95
C31-C32	1.514(4)	C93-C94	1.388(4)
C31-H311	0.99	C93-H93	0.95
C31-H312	0.99	C94-C95	1.387(4)
C32-H321	0.99	C94-H94	0.95
C32-H322	0.99	C95-C96	1.385(4)
C40-C45	1.499(4)	C95-H95	0.95
C40-C51	1.538(4)	C96-H96	0.95
C40-H40	1.00		

N11-C1-C7	107.6(2)	C16-N11-C1	113.8(2)
N11-C1-C8	108.9(2)	N11-C12-C13	110.9(2)
C7-C1-C8	106.7(2)	N11-C12-H121	109.5
N11-C1-C2	111.6(2)	C13-C12-H121	109.5
C7-C1-C2	111.1(3)	N11-C12-H122	109.5
C8-C1-C2	110.7(2)	C13-C12-H122	109.5
N3-C2-C1	115.7(2)	H121-C12-H122	108.0
N3-C2-H21	108.4	C14-C13-C12	111.6(3)
C1-C2-H21	108.4	C14-C13-H131	109.3
N3-C2-H22	108.4	C12-C13-H131	109.3
C1-C2-H22	108.4	C14-C13-H132	109.3
H21-C2-H22	107.4	C12-C13-H132	109.3
C32-N3-C2	107.5(2)	H131-C13-H132	108.0
C32-N3-C4	109.4(2)	C15-C14-C13	109.9(2)
C2-N3-C4	115.1(2)	C15-C14-H141	109.7
N3-C4-C5	115.8(2)	C13-C14-H141	109.7
N3-C4-H41	108.3	C15-C14-H142	109.7
C5-C4-H41	108.3	C13-C14-H142	109.7
N3-C4-H42	108.3	H141-C14-H142	108.2
C5-C4-H42	108.3	C14-C15-C16	110.9(3)
H41-C4-H42	107.4	C14-C15-H151	109.5
C4-C5-C9	114.4(2)	C16-C15-H151	109.5
C4-C5-C6	111.1(2)	C14-C15-H152	109.5
C9-C5-C6	109.8(2)	C16-C15-H152	109.5
C4-C5-H5	107.1	H151-C15-H152	108.0
C9-C5-H5	107.1	N11-C16-C15	111.0(3)
C6-C5-H5	107.1	N11-C16-H161	109.4
C61-C6-C7	110.3(2)	C15-C16-H161	109.4
C61-C6-C5	115.7(2)	N11-C16-H162	109.4
C7-C6-C5	110.5(2)	C15-C16-H162	109.4
C61-C6-H6	106.6	H161-C16-H162	108.0
C7-C6-H6	106.6	C31-N30-C40	113.3(2)
C5-C6-H6	106.6	C31-N30-H30	115.6(18)
C1-C7-C6	111.5(2)	C40-N30-H30	106.6(18)
C1-C7-H71	109.3	N30-C31-C32	110.2(3)
C6-C7-H71	109.3	N30-C31-H311	109.6
C1-C7-H72	109.3	C32-C31-H311	109.6
C6-C7-H72	109.3	N30-C31-H312	109.6
H71-C7-H72	108.0	C32-C31-H312	109.6
C1-C8-C9	112.6(2)	H311-C31-H312	108.1
C1-C8-H81	109.1	N3-C32-C31	115.3(2)
C9-C8-H81	109.1	N3-C32-H321	108.5
C1-C8-H82	109.1	C31-C32-H321	108.5
C9-C8-H82	109.1	N3-C32-H322	108.5
H81-C8-H82	107.8	C31-C32-H322	108.5
C91-C9-C5	115.6(2)	H321-C32-H322	107.5
C91-C9-C8	111.9(2)	N30-C40-C45	106.3(2)
C5-C9-C8	111.1(2)	N30-C40-C51	114.1(2)
C91-C9-H9	105.8	C45-C40-C51	112.3(2)
C5-C9-H9	105.8	N30-C40-H40	108.0
C8-C9-H9	105.8	C45-C40-H40	108.0
C12-N11-C16	108.5(2)	C51-C40-H40	108.0
C12-N11-C1	113.7(2)	N42-N41-C45	107.9(2)

N42-N41-C41	120.1(2)
C45-N41-C41	131.9(3)
N43-N42-N41	107.0(2)
N42-N43-N44	110.4(3)
C45-N44-N43	106.5(2)
N44-C45-N41	108.3(3)
N44-C45-C40	123.0(3)
N41-C45-C40	128.7(3)
N41-C41-C44	109.3(2)
N41-C41-C42	108.0(3)
C44-C41-C42	109.1(3)
N41-C41-C43	107.5(2)
C44-C41-C43	112.2(3)
C42-C41-C43	110.6(3)
C41-C42-H421	109.5
C41-C42-H422	109.5
H421-C42-H422	109.5
C41-C42-H423	109.5
H421-C42-H423	109.5
H422-C42-H423	109.5
C41-C43-H431	109.5
C41-C43-H432	109.5
H431-C43-H432	109.5
C41-C43-H433	109.5
H431-C43-H433	109.5
H432-C43-H433	109.5
C41-C44-H441	109.5
C41-C44-H442	109.5
H441-C44-H442	109.5
C41-C44-H443	109.5
H441-C44-H443	109.5
H442-C44-H443	109.5
C52-C51-C56	119.0(3)
C52-C51-C40	119.7(3)
C56-C51-C40	121.1(3)
C51-C52-C53	120.7(3)
C51-C52-H52	119.7
C53-C52-H52	119.7
C54-C53-C52	119.8(3)
C54-C53-H53	120.1
C52-C53-H53	120.1
C55-C54-C53	120.0(3)
C55-C54-H54	120.0
C53-C54-H54	120.0
C54-C55-C56	120.2(3)
C54-C55-H55	119.9
C56-C55-H55	119.9
C51-C56-C55	120.4(3)
C51-C56-H56	119.8
C55-C56-H56	119.8
C62-C61-C66	117.7(3)
C62-C61-C6	119.7(3)
C66-C61-C6	122.5(3)
C61-C62-C63	121.1(3)
C61-C62-H62	119.5
C63-C62-H62	119.5
C64-C63-C62	120.3(3)
C64-C63-H63	119.8
C62-C63-H63	119.8
C63-C64-C65	119.5(3)
C63-C64-H64	120.3
C65-C64-H64	120.3
C64-C65-C66	120.0(3)
C64-C65-H65	120.0
C66-C65-H65	120.0
C65-C66-C61	121.4(3)
C65-C66-H66	119.3
C61-C66-H66	119.3
C92-C91-C96	117.9(3)
C92-C91-C9	120.1(3)
C96-C91-C9	121.9(3)
C93-C92-C91	121.6(3)
C93-C92-H92	119.2
C91-C92-H92	119.2
C92-C93-C94	120.1(3)
C92-C93-H93	119.9
C94-C93-H93	119.9
C95-C94-C93	119.0(3)
C95-C94-H94	120.5
C93-C94-H94	120.5
C96-C95-C94	120.7(3)
C96-C95-H95	119.6
C94-C95-H95	119.6
C95-C96-C91	120.7(3)
C95-C96-H96	119.6
C91-C96-H96	119.6

N11-C1-C2-N3	-143.3(2)
C7-C1-C2-N3	96.6(3)
C8-C1-C2-N3	-21.7(3)
C1-C2-N3-C32	175.6(2)
C1-C2-N3-C4	-62.2(3)
C32-N3-C4-C5	163.8(2)
C2-N3-C4-C5	42.7(4)
N3-C4-C5-C9	50.4(3)
N3-C4-C5-C6	-74.6(3)
C4-C5-C6-C61	-138.4(3)
C9-C5-C6-C61	94.1(3)
C4-C5-C6-C7	95.2(3)
C9-C5-C6-C7	-32.3(3)
N11-C1-C7-C6	-172.6(2)
C8-C1-C7-C6	70.6(3)
C2-C1-C7-C6	-50.1(3)
C61-C6-C7-C1	-162.0(2)
C5-C6-C7-C1	-32.7(3)
N11-C1-C8-C9	-154.1(2)
C7-C1-C8-C9	-38.3(3)
C2-C1-C8-C9	82.7(3)
C4-C5-C9-C91	66.4(3)
C6-C5-C9-C91	-167.9(2)
C4-C5-C9-C8	-62.6(3)
C6-C5-C9-C8	63.1(3)
C1-C8-C9-C91	-156.0(2)
C1-C8-C9-C5	-25.1(3)
C7-C1-N11-C12	-164.4(2)
C8-C1-N11-C12	-49.1(3)
C2-C1-N11-C12	73.5(3)
C7-C1-N11-C16	70.7(3)
C8-C1-N11-C16	-174.0(2)
C2-C1-N11-C16	-51.4(3)
C16-N11-C12-C13	-61.5(3)
C1-N11-C12-C13	170.8(2)
N11-C12-C13-C14	57.4(3)
C12-C13-C14-C15	-51.8(4)
C13-C14-C15-C16	52.0(4)
C12-N11-C16-C15	62.2(3)
C1-N11-C16-C15	-170.1(2)
C14-C15-C16-N11	-58.3(3)
C40-N30-C31-C32	169.3(2)
C2-N3-C32-C31	-166.2(2)
C4-N3-C32-C31	68.2(3)
N30-C31-C32-N3	73.0(3)
C31-N30-C40-C45	-177.7(2)
C31-N30-C40-C51	58.0(3)
C45-N41-N42-N43	-0.5(3)
C41-N41-N42-N43	-177.5(2)
N41-N42-N43-N44	0.3(3)
N42-N43-N44-C45	0.0(3)
N43-N44-C45-N41	-0.3(3)
N43-N44-C45-C40	177.3(3)

N42-N41-C45-N44	0.5(3)
C41-N41-C45-N44	177.0(3)
N42-N41-C45-C40	-176.9(3)
C41-N41-C45-C40	-0.4(5)
N30-C40-C45-N44	-48.5(4)
C51-C40-C45-N44	76.9(3)
N30-C40-C45-N41	128.6(3)
C51-C40-C45-N41	-106.0(3)
N42-N41-C41-C44	-133.1(3)
C45-N41-C41-C44	50.8(4)
N42-N41-C41-C42	-14.5(4)
C45-N41-C41-C42	169.4(3)
N42-N41-C41-C43	105.0(3)
C45-N41-C41-C43	-71.2(4)
N30-C40-C51-C52	-101.7(3)
C45-C40-C51-C52	137.3(3)
N30-C40-C51-C56	73.0(4)
C45-C40-C51-C56	-48.0(4)
C56-C51-C52-C53	0.1(5)
C40-C51-C52-C53	174.9(3)
C51-C52-C53-C54	-0.1(5)
C52-C53-C54-C55	0.1(5)
C53-C54-C55-C56	0.0(5)
C52-C51-C56-C55	0.0(5)
C40-C51-C56-C55	-174.8(3)
C54-C55-C56-C51	0.0(5)
C7-C6-C61-C62	-120.6(3)
C5-C6-C61-C62	113.0(3)
C7-C6-C61-C66	56.5(4)
C5-C6-C61-C66	-69.9(4)
C66-C61-C62-C63	-2.4(4)
C6-C61-C62-C63	174.9(3)
C61-C62-C63-C64	1.4(5)
C62-C63-C64-C65	0.4(5)
C63-C64-C65-C66	-1.1(5)
C64-C65-C66-C61	0.1(5)
C62-C61-C66-C65	1.6(4)
C6-C61-C66-C65	-175.6(3)
C5-C9-C91-C92	106.6(3)
C8-C9-C91-C92	-124.8(3)
C5-C9-C91-C96	-76.2(4)
C8-C9-C91-C96	52.4(4)
C96-C91-C92-C93	-0.1(4)
C9-C91-C92-C93	177.2(3)
C91-C92-C93-C94	-0.2(5)
C92-C93-C94-C95	0.6(5)
C93-C94-C95-C96	-0.6(5)
C94-C95-C96-C91	0.3(5)
C92-C91-C96-C95	0.1(4)
C9-C91-C96-C95	-177.2(3)
